# A Residence-Time Approach for Determining Position-Dependent Diffusivities from Biased Molecular Simulations

**DOI:** 10.1021/acs.jctc.6c00645

**Published:** 2026-08-28

**Authors:** Rinto Thomas, Praveen Ranganath Prabhakar, Michael von Domaros

**Affiliations:** † Fachbereich Chemie, Philipps-Universität Marburg, 35032 Marburg, Germany; ‡ Department of Chemistry, University of California, Irvine, Irvine, California 92697, United States

## Abstract

Position-dependent diffusivities are central parameters in reduced stochastic descriptions of molecular transport in heterogeneous environments, but their reliable estimation from molecular dynamics simulations remains challenging. We present a residence-time approach (RTA) that extracts local diffusivities from first-exit statistics measured in biased simulations after compensation of the mean free-energy gradient. We apply the method to oxygen diffusion across a hexadecane/water slab, water permeation across a POPC lipid bilayer, and transport of water and volatile organic compounds through a model skin-barrier membrane. In the slab system, RTA diffusivities agree with independently determined bulk reference values. In the membrane systems, propagator predictions based on RTA-derived diffusivities reproduce unbiased molecular dynamics propagators over substantial lag-time ranges, while also revealing that, in some cases, no single lag-time-independent diffusivity profile captures the dynamics across all time scales. These results support residence-time statistics as a practical route for determining effective position-dependent diffusivities from biased molecular simulations.

## Graphical abstract

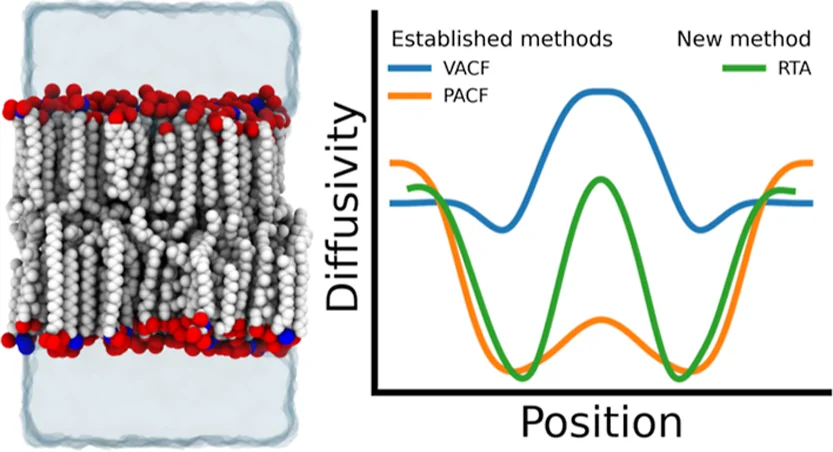

## 1 Introduction

Diffusivities provide a compact and physically interpretable description of complex many-body dynamics, whether generated by molecular dynamics (MD) simulations or observed in natural systems, in terms of effective transport coefficients.^1,2^ By averaging over microscopic degrees of freedom, a diffusivity captures how interactions and solvent-induced fluctuations determine transport on mesoscopic scales. In homogeneous environments, this coarse-grained description reduces to a single scalar diffusivity. In heterogeneous systems such as membranes, nanopores, and porous materials, however, transport is more naturally described by a position-dependent diffusivity *D*(*z*) along a chosen transport coordinate *z*.^3^

In a continuum description, position-dependent diffusivities arise naturally within stochastic transport models.^4^ Within this framework, the probability density *p*(*z*, *t*) evolves according to the Smoluchowski equation

1 $$\partial_{t}p\left(z,t\right)=\partial_{z}\left[D\left(z\right)e^{-\beta F\left(z\right)}\partial_{z}\left(e^{\beta F\left(z\right)}p\left(z,t\right)\right)\right]$$

where *p*(*z*, *t*) is the probability density and *F*(*z*) is the potential of mean force (PMF) along *z*, defined relative to the bulk phase such that *F*(*z*_bulk_) = 0. In this effective one-dimensional description, *F*(*z*) and *D*(*z*) encode the thermodynamic and kinetic consequences of projecting high-dimensional dynamics onto a reduced coordinate. Equation 1 represents a diffusive, memoryless (Markovian) description of the projected dynamics along *z*.

A particularly important application is membrane permeation, where the steady-state solution of eq 1 yields the inhomogeneous solubility–diffusion (ISD) model^5,6^

2 $$P^{-1}=\int_{z_{1}}^{z_{2}}\frac{e^{\beta F\left(z\right)}}{D\left(z\right)}dz$$

which relates the permeability *P* to the PMF *F*(*z*) and diffusivity *D*(*z*) between boundaries *z*_1_ and *z*_2_ that delimit the membrane region. This relation provides a direct quantitative bridge between microscopic simulations and experimentally measurable permeabilities, provided that both *F*(*z*) and *D*(*z*) are determined with sufficient accuracy.

Accurate determination of *D*(*z*) is therefore essential for connecting molecular-scale dynamics to macroscopic transport properties. Extracting reliable position-dependent diffusivities from MD trajectories, however, remains challenging, and a wide range of approaches has been developed.^6–9^ Broadly, these methods include equilibrium fluctuation-based estimators based on restrained trajectories and time-correlation functions,^3,10–12^ likelihood-based inference of Smoluchowski dynamics from short-time propagators,^3,13–15^ local-statistics approaches that estimate diffusivities from drift-free trajectory segments or local mean-squared displacements within finite observation domains,^16,17^ discrete-state kinetic reconstructions such as Markov state models and milestoning,^18,19^ and short-lag moment or Kramers–Moyal estimators.^20^ Among these, fluctuation-based estimators obtained from harmonically restrained simulations are by far the most widely used in practice.

Many of these estimators are sensitive to analysis choices and model assumptions. Recent studies suggest that external confinement can modify the effective friction and memory kernel experienced by a solute, implying that diffusivities extracted from harmonically restrained dynamics need not coincide with those obtained from freely diffusing trajectories.^21,22^ Consequently, discrepancies between different estimators may reflect differences in the effective dynamics induced by the underlying sampling protocol rather than solely methodological shortcomings. Correlation-function approaches furthermore require numerical integration or extrapolation of noisy time-correlation data and may depend strongly on restraint strength and on the chosen integration or extrapolation scheme.^3,10,11^ Inference-based schemes can exhibit lag-time dependence and may be affected by discretization, regularization, or parametrization bias.^3,14^ Local-statistics approaches require the definition of a finite observation domain and appropriate treatment of finite-window effects,^16,17^ whereas discrete-state approaches require suitable state decompositions and adequate transition statistics, and short-lag moment estimators rely on a well-resolved regime in which the projected dynamics are approximately Markovian.^18–20^

More fundamentally, the projection of high-dimensional molecular motion onto a single reduced coordinate can introduce residual memory effects and incomplete separation of time scales, especially in heterogeneous environments such as membranes.^23–25^ As a result, diffusivity estimates may depend on lag time or on the operational definition of the reduced dynamics, reflecting deviations from an idealized memoryless Smoluchowski description. In many applications, however, the primary goal is not to resolve non-Markovian effects explicitly, but to construct an effective diffusive description that reproduces the transport observables of interest. Such a description may remain accurate for these observables even if the Markovian approximation breaks down in parts of the system.

Here, we introduce a residence-time approach (RTA) for estimating position-dependent diffusivities from biased MD simulations. The central idea is to determine *D*(*z*) from mean first-exit times out of predefined spatial intervals along a transport collective variable (CV) *z*. Similar to previous approaches based on Bayesian inference and local displacement statistics,^15–17^ the present method is formulated for trajectory segments in which the effective drift along the CV is negligible. In principle, such a drift-free regime can be realized using any enhanced-sampling method that provides an estimate of the underlying free-energy profile or mean force, allowing a compensating bias to flatten the effective free-energy landscape. In the present work, we employ adaptive biasing force (ABF) simulations, for which this condition arises naturally as the estimated mean force converges, providing a convenient transition from free-energy to diffusivity estimation.

Our estimator is designed to avoid several practical limitations of established fluctuation-based approaches. It does not require harmonic confinement or dedicated restrained simulations, and it avoids numerical integration or extrapolation of noisy time-correlation functions.^3,10,11^ Instead, it determines local diffusivities directly from residence-time statistics in an approximately drift-free landscape, making the method straightforward to implement and naturally compatible with blocking analysis for uncertainty estimation.

We assess the method in two complementary ways. First, where bulk reference diffusivities are accessible, we compare the RTA estimates directly against independently determined bulk values. Second, and more generally, we perform propagator-level validation in heterogeneous environments: diffusivity profiles inferred from the RTA are combined with the PMF to compute model propagators, i.e., time-dependent conditional probability distributions, which are then compared with the same quantity obtained from unbiased MD simulations. Comparison between model and simulation propagators over a range of lag times provides a direct test of how well the inferred Smoluchowski model captures the projected dynamics.

The remainder of this article is organized as follows. We first develop the theoretical basis of the RTA and its connection to the drift-free limit of biased dynamics, then describe its implementation for ABF trajectories and apply it to systems ranging from simple liquid–liquid diffusion to increasingly heterogeneous membrane environments.

## 2 Theory

### 2.1 Drift-Free Limit of Biased Dynamics

As introduced above, the projected dynamics along a transport coordinate *z* are modeled here by the Smoluchowski equation, eq 1, with position-dependent diffusivity *D*(*z*) and PMF *F*(*z*). The residence-time approach developed below applies to trajectory segments in which the effective drift along *z* is negligible. This condition can be realized by applying a bias potential *V*_b_(*z*) that compensates the underlying free-energy gradient, so that the effective free-energy profile

3 $$F_{eff}\left(z\right)=F\left(z\right)+V_{b}\left(z\right)$$

is approximately constant over the region analyzed.

In the present work, this regime is realized using adaptive biasing force (ABF) sampling.^26,27^ ABF estimates the mean force along a CV during the simulation and applies a history-dependent biasing force that progressively compensates this mean force. In one dimension, the biasing force approaches

4 $$-\frac{\partial V_{b}\left(z,t\right)}{\partial z}\approx \frac{dF\left(z\right)}{dz}$$

so that, as the estimate converges, *F*_eff_(*z*) becomes approximately flat. The residual effective drift then becomes small on the spatial scale over which the bias has converged.

In this drift-free limit, the Smoluchowski equation reduces locally to

5 $$\partial_{t}p\left(z,t\right)=\partial_{z}\left[D\left(z\right)\partial_{z}p\left(z,t\right)\right]$$

which describes diffusion in the absence of an effective free-energy gradient. This is the central condition underlying the residence-time approach. In practice, the analysis must therefore be restricted to regions and trajectory intervals for which the ABF bias has converged sufficiently that residual drift is negligible on the scale of the residence-time intervals.

### 2.2 Residence-Time Approach

#### 2.2.1 First-Exit Times in an Interval

Consider an interval Ω = [*a*,*b*) along *z* with width *L* = *b* – *a*. For the derivation, we assume that the diffusivity is constant within the interval, *D*(*z*) = *D* for *z* ∈ Ω. The first-exit time *T* is the random time at which a trajectory leaves Ω for the first time

6 T=inf{t≥0:z(t)∉Ω}

For an initial condition *z*(0) = *z*_0_ ∈ (*a*, *b*), the mean first-exit time (MFET)

$$\tau \left(z_{0}\right)\equiv \left\langle T|z\left(0\right)=z_{0}\right\rangle$$ 7

is the average time required to leave Ω when starting at *z*_0_.

For drift-free Markovian diffusion with constant diffusivity in the interval, τ­(*z*_0_) satisfies a standard backward equation.^28,29^ This result can be obtained by a one-step argument. Writing

8 $$\tau \left(z_{0}\right)=\delta t+\left\langle \tau \left(z_{0}+\delta z\right)\right\rangle$$

with δ*z* = *z*(δ*t*) – *z*(0), and using ⟨δ*z*⟩ = 0 and ⟨(δ*z*)^2^⟩ = 2*D* δ*t* for drift-free diffusion, a second-order expansion in δ*z* yields

9 $$D\tau^{\prime \prime }\left(z_{0}\right)=-1,,\tau \left(a\right)=0,,\tau \left(b\right)=0$$

The absorbing boundary conditions express that the first-exit time vanishes when the trajectory starts at either boundary. Solving eq 9 gives

10 $$\tau \left(z_{0}\right)=\frac{\left(z_{0}-a\right)\left(b-z_{0}\right)}{2D}$$

#### 2.2.2 Residence-Time Identity

We define the residence time τ_*r*_ as the MFET averaged over initial positions *z*_0_ within Ω

11 $$\tau_{r}={\left\langle T\right\rangle }_{\Omega }=\int_{a}^{b}\rho_{\Omega }\left(z_{0}\right)\tau \left(z_{0}\right)dz_{0}$$

where ρ_Ω_(*z*_0_) denotes the equilibrium distribution of positions conditioned on the particle being inside the interval. As in eq 7, *z*_0_ denotes the initial position for a first-exit problem, not necessarily the position at which a trajectory enters the interval. In the trajectory analysis, each sampled frame for which the particle is inside Ω defines such a possible initial condition, and the corresponding residence time is the remaining first-exit time from that position. Re-entry into the interval is therefore accounted for through later sampled frames, rather than by extending a previous residence event.

For drift-free diffusion in a flat effective PMF, the conditional equilibrium distribution is uniform within the interval, so that ρ_Ω_(*z*_0_) = 1/*L*. Inserting eq 10 into eq 11 then gives

12 $$\tau_{r}=\frac{L^{2}}{12D}$$

This relation is exact for one-dimensional drift-free diffusion with constant diffusivity inside the interval.

An equivalent derivation follows from solving the diffusion equation on Ω with absorbing boundaries and then integrating the corresponding survival probability over time to obtain the mean residence time.^30,31^

#### 2.2.3 Local Estimator for Position-Dependent Diffusivities

To estimate a position-dependent diffusivity, we discretize the transport coordinate into intervals Ω_*i*_ = [*z*_*i*_,*z*_*i*+1_) of width *L*_*i*_ = *z*_*i*+1_ – *z*_*i*_. Within each interval, the diffusivity is approximated as locally constant, *D*(*z*) ≈ *D*_*i*_ for *z* ∈ Ω_*i*_, such that spatial variations contribute only at higher order in *L*_*i*_.

Let τ_*r*,*i*_ denote the theoretical mean residence time in interval Ω_*i*_. By applying the residence-time identity, eq 12, locally to each interval, we obtain the estimator

13 $${\mathrm{D̂}}_{i}=\frac{L_{i}^{2}}{12{\mathrm{\tau ̂}}_{r,i}}$$

where ${\mathrm{\tau ̂}}_{r,i}$ is the corresponding interval-averaged residence time estimated from the trajectory.

In practice, the residence-time identity is applied to biased MD trajectories only after the ABF bias has converged sufficiently that the effective drift along the CV is negligible over the interval considered. The procedure used to estimate ${\mathrm{\tau ̂}}_{r,i}$ from sampled trajectories is described in the Computational Details section.

The resulting estimator rests on three central approximations. First, the diffusivity is treated as locally constant within each interval. Second, the effective drift is assumed to be negligible within the analyzed region, so that the reduced dynamics are well approximated by eq 5. Third, the projected dynamics are assumed to be sufficiently close to Markovian on the spatial and temporal scales probed by the residence-time analysis. Accordingly, the quantity ${\mathrm{D̂}}_{i}$ should be interpreted as an effective first-passage diffusivity associated with the chosen residence interval rather than as a unique local transport coefficient. The quality of these approximations is assessed below through comparison to independent bulk diffusivities and, more rigorously, through propagator-level validation in heterogeneous membrane systems. Additional local MSD and survival-probability analyses are provided in Section S5.1, where this interpretation is further examined.

## 3 Computational Details

### 3.1 Model Systems

We evaluated the residence-time approach (RTA) for three systems of increasing complexity (Figure 1): oxygen diffusion across a hexadecane/water slab, water permeation across a POPC lipid bilayer, and permeation of water, acetone, and 6-methyl-5-hepten-2-one (6-MHO) through a multicomponent lipid membrane representing the stratum corneum (SC) skin barrier. System compositions and simulation box dimensions are summarized in Table 1.

**Figure 1:**
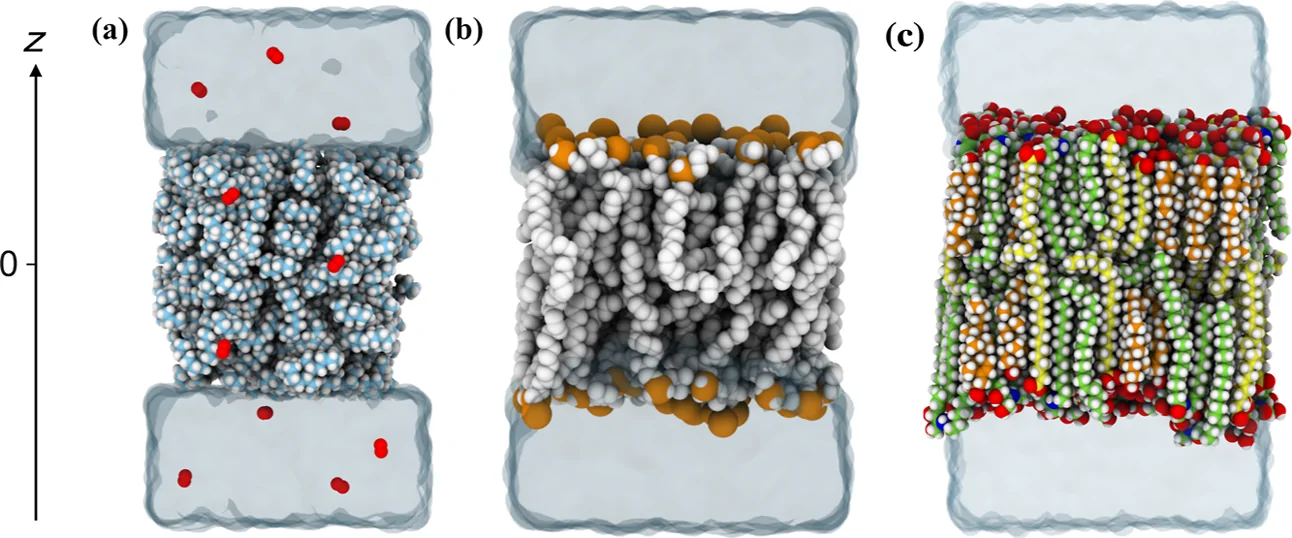
Representative snapshots of the model systems investigated in this work. (a) Hexadecane/water interface, with the diffusing oxygen atoms highlighted as red spheres. (b) POPC lipid bilayer. (c) Stratum corneum (SC) lipid matrix composed of ceramide NS (green), cholesterol (orange), and lignoceric acid (yellow). The transport coordinate *z* and the reference position *z* = 0, common to all three systems, are indicated on the left.

**Table 1:** Compositions of the Simulated Systems and Corresponding Simulation Box Dimensions

system	composition	*L*_*x*_ (Å)	*L*_*y*_ (Å)	*L*_*z*_ (Å)
hexadecane/water	252 hexadecane, 4318 water, 10 O_2_	50.00	50.00	105.00
POPC/water	72 POPC, 2967 water	48.43	48.43	76.34
SC/water	200 SC lipids, 4800 water	57.14	57.14	95.14
SC/acetone	200 SC lipids, 4800 water, 1 acetone	57.14	57.14	95.35
SC/6-MHO	200 SC lipids, 4800 water, 1 6-MHO	57.14	57.14	95.14

SC lipids comprise an approximately equimolar mixture of cholesterol, lignoceric acid, and ceramide NS (24:0).^32^ For the POPC/water and SC/water systems, one water molecule was designated as the permeating solute for analysis.

The hexadecane/water system serves as a simple liquid–liquid reference with low free-energy barriers and well-defined bulk regions, enabling direct comparison with diffusivities obtained from the Einstein relation. Our primary slab system is approximately twice as large as that of Ghysels et al.,^13^ yielding more extended bulk regions; a smaller comparison system more closely matching their setup is described in Section S1.4.

Water permeation across a POPC bilayer is a widely used benchmark for transport in fluid-phase lipid membranes and has been investigated extensively in molecular dynamics (MD) simulations.^6,8,33–36^

The SC membrane represents a more complex and application-relevant system. Here we consider permeation of water, acetone, and 6-MHO through a multicomponent SC lipid matrix, building on our previous work on the transport of skin-oil oxidation products through the SC barrier.^32,37^

All simulations employed the CHARMM36 lipid force field together with the CHARMM General Force Field (CGenFF) for small molecules and the TIP3P water model.^38–40^ The oxygen model was adopted from ref 13.

Initial coordinates for the hexadecane/water system were generated using Packmol,^41^ whereas initial configurations for the POPC bilayer and SC membrane systems were taken from our previous studies.^32,37^

### 3.2 Simulation Protocol

All simulations were performed using NAMD 3.0.1.^42,43^ A multiple time-step integration scheme based on the r-RESPA algorithm^44^ was employed with a base time step of 2 fs. Short-range nonbonded interactions were evaluated every 2 fs, while long-range electrostatic interactions were evaluated every 4 fs.

Water molecules were maintained as rigid bodies using the SETTLE algorithm,^45^ and all other bonds involving hydrogen atoms were constrained using the SHAKE algorithm.^46^

Periodic boundary conditions were applied in all three spatial directions. Short-range nonbonded interactions were switched between 10 Å and 12 Å. Long-range electrostatic interactions were computed using the smooth particle mesh Ewald (SPME) method^47^ with a real-space cutoff of 12 Å, a grid spacing of 1 Å, and sixth-order spline interpolation.

All simulations were carried out at 310 K, except for the SC membrane system, which was simulated at the physiological skin temperature of 305.15 K. Temperature was controlled using the stochastic velocity-rescaling thermostat of Bussi et al.^48^ with a time constant of 1 ps.

The POPC and SC membrane systems were initialized from equilibrated configurations obtained in previous studies, where the preparation and equilibration procedures are described in detail.^32,37^

For the hexadecane/water system, energy minimization was performed for 100000 steps, followed by 0.5 ns of NVT equilibration. This was followed by 40 ns of equilibration at 1 bar with the lateral box dimensions fixed (constant area), using a Langevin piston barostat^49,50^ with a relaxation time of 50 fs and an oscillation period of 100 fs. A final 40 ns equilibration phase was then performed in the NVT ensemble.

All production simulations were carried out in the NVT ensemble using simulation boxes with dimensions summarized in Table 1.

### 3.3 Adaptive Biasing Force Simulations

ABF simulations were performed with the Colvars library in NAMD 3.0.1.^27,51^ The CV used for biased sampling and subsequent residence-time analysis was defined as the position of the solute center of mass along the membrane normal (*z*), measured relative to the center of the membrane or slab, as illustrated in Figure 1. The membrane or slab remained continuous along *z*, which is the only coordinate used in the one-dimensional transport analysis.

Mean forces were accumulated in 0.1 Å-wide bins along the CV. Biasing forces were introduced gradually using a linear ramp from 0 to 1 until 1000 force samples had been collected in each bin. Harmonic boundary potentials with a force constant of 5 kcal mol^–1^ Å^–2^ were applied at the boundaries of the sampled CV interval. These boundaries were placed at the ends of the bulk aqueous regions, so that the solute remained within the primary simulation cell along *z* and periodic-boundary crossings did not introduce ambiguities in the center-of-mass coordinate.

For the hexadecane/water and POPC/water systems, the ABF bias was accumulated in a single continuous window spanning the full CV interval. For the SC membrane systems, the CV was stratified into seven overlapping ABF windows to enable parallel sampling across the membrane, following the scheme previously used by Schow et al.,^52^ with adjacent windows overlapping by 5 Å. Details of the window definitions are provided in Section S3.1.

The sufficient convergence of the ABF bias required for the RTA was assessed operationally from the time evolution of the PMF. Once the PMF became stationary and symmetric within statistical uncertainty, the residual drift was taken to be negligible on the spatial scale of the residence-time intervals used in the analysis.

### 3.4 Harmonically Restrained Simulations

For comparison with established equilibrium fluctuation-based diffusivity estimators, we report position-dependent diffusivities obtained from our previous study for the POPC and SC membrane systems.^37^ In that work, the solute was harmonically restrained at fixed positions along the CV to generate trajectories suitable for fluctuation-based analysis.

Position-dependent diffusivities were determined using two commonly employed estimators based on the velocity and position autocorrelation functions (VACF and PACF).^3,10^

In the VACF-based approach of Woolf and Roux,^10^ the velocity autocorrelation function

14 $$C_{v}\left(t\right)=\left\langle ż\left(t\right)ż\left(0\right)\right\rangle$$

is used to construct a Laplace-frequency-dependent diffusivity

15 $$D\left(s\right)=\frac{-{Ĉ}_{v}\left(s\right)\left\langle \delta z^{2}\right\rangle \left\langle {ż}^{2}\right\rangle }{{Ĉ}_{v}\left(s\right)\left[s\left\langle \delta z^{2}\right\rangle +s^{-1}\left\langle {ż}^{2}\right\rangle \right]-\left\langle \delta z^{2}\right\rangle \left\langle {ż}^{2}\right\rangle }$$

which is extrapolated to zero frequency

16 $$D\left(z=\left\langle z\right\rangle \right)=\lim_{s\to 0}D\left(s\right)$$

In the PACF-based approach introduced by Hummer,^3^ the normalized position autocorrelation function

17 $$C_{\delta z}\left(t\right)=\frac{\left\langle \delta z\left(t\right)\delta z\left(0\right)\right\rangle }{\left\langle \delta z^{2}\right\rangle }$$

yields

18 $$D\left(z=\left\langle z\right\rangle \right)=\frac{\left\langle \delta z^{2}\right\rangle }{\int_{0}^{\infty }C_{\delta z}\left(t\right)dt}$$

Full simulation and analysis details for the restrained trajectories are provided in ref 37.

### 3.5 Residence-Time Analysis

Mean residence times were estimated from the trajectory time series *z*(*t*) by identifying contiguous trajectory segments that remained within a given interval Ω_*i*_ until first exit.

For a segment containing *m* consecutive frames inside interval Ω_*i*_, each frame was treated as a possible starting point and assigned the remaining time until first exit. The associated exit times are therefore

mΔt,(m−1)Δt,...,Δt

where Δ*t* is the trajectory sampling interval.

The interval-averaged residence time was estimated as

19 $${\mathrm{\tau ̂}}_{r,i}=\frac{1}{N_{i}}\sum_{k=1}^{N_{i}}T_{i,k}$$

where *T*_*i*,*k*_ is the first-exit time associated with the *k*-th starting frame in interval Ω_*i*_, and *N*_*i*_ is the total number of starting frames assigned to that interval. Local diffusivities were then obtained from the residence-time estimator derived in Theory, eq 13.

An interval width *L* = 7.5 Å was used for the residence-time analysis in all systems. This choice reflects the inherent coarse-graining of the RTA: *L* must be sufficiently large for the first-passage statistics within an interval to be well described by an effective diffusive model, yet sufficiently small to resolve spatial variations in the transport properties. To assess the sensitivity of the results to this choice, we analyze the dependence of the estimated diffusivities on *L* for representative regions of the SC/water system in Section S5.3. The observed plateau over a finite range of *L* indicates that the reported diffusivities are robust with respect to the precise choice of domain width within this regime. The broader implications of the choice of *L* are discussed further in the Conclusions and Outlook section.

### 3.6 Uncertainty Estimation

Because the residence times extracted from a continuous MD trajectory are temporally correlated, statistical uncertainties cannot be obtained by assuming independent samples. We therefore estimated uncertainties using the blocking transformation method of Flyvbjerg and Petersen,^53^ which determines the asymptotic variance without assuming an explicit correlation model.

Specifically, we employed the automated blocking procedure of Jonsson,^54^ which identifies the asymptotic variance regime and estimates the effective number of independent samples. Uncertainties in ${\mathrm{\tau ̂}}_{r,i}$ were propagated to ${\mathrm{D̂}}_{i}$ by standard error propagation. All reported uncertainty intervals correspond to 95% confidence intervals.

As an additional consistency check, we compared the automated blocking procedure with a variance-normalization approach for correlated data.^55^ Both methods yield closely similar estimates of the asymptotic variance for the residence-time data considered here; details are provided in Section S5.2.

### 3.7 Propagator Analysis

To assess the predictive accuracy of the reduced transport models, we compared propagators predicted from the Smoluchowski equation with propagators obtained directly from unbiased MD simulations.

Given a PMF *F*(*z*) and a position-dependent diffusivity *D*(*z*), the time evolution of the probability density *p*(*z*, *t*) is governed by eq 1. For a delta-function initial condition *p*(*z*, *t*_0_) = δ­(*z* – *z*_0_), the solution yields the propagator *p*(*z*, *t* |*z*_0_, *t*_0_).

Reference propagators obtained directly from unbiased MD simulations for the POPC and SC systems were previously reported in ref 37. In that work, initial configurations were selected from umbrella-sampling trajectories for which the solute position satisfied |*z* – *z*_0_| < 0.05 Å, providing a narrow initial distribution that approximates the delta-function condition. From each selected configuration, the umbrella restraint was then removed and the system was propagated without bias. Histograms of the solute position were accumulated to estimate conditional probability distributions *p*(*z*, *t* |*z*_0_) at different lag times.

Model propagators were obtained by numerically solving the Smoluchowski equation using a forward-in-time, centered-in-space finite-difference scheme as described in ref 37. Because the diffusivity estimators yield discrete values at sampled positions, the corresponding *D*(*z*) profiles were represented by smoothing splines before solving the Smoluchowski equation. The spline interpolation employs zero first- and second-derivative boundary conditions at the ends of the sampled interval, providing a smooth representation of the discrete diffusivity profile for the numerical solution.

### 3.8 Permeability Calculations

Permeability coefficients for the POPC and SC membrane systems were computed from the PMF and diffusivity profiles using the inhomogeneous solubility–diffusion model, eq 2. For each system, the integration bounds *z*_1_ and *z*_2_ were determined from the corresponding density profiles. The specific boundary definitions and numerical permeability coefficients are reported in Section S4.

## 4 Results and Discussion

### 4.1 Oxygen Diffusion in a Hexadecane/Water Slab

We first consider oxygen diffusion across a hexadecane/water slab, which serves as a simple heterogeneous reference system with well-defined bulk regions (Section S1.2). This system provides the most direct test of the residence-time approach because independently determined bulk diffusivities are available in both phases.

Applying the RTA requires trajectory segments for which the effective PMF is approximately flat. Convergence of the ABF bias was assessed from the time evolution of the PMF (Figure 2). From 800 ns onward, the PMF remains stationary and symmetric within statistical uncertainty, indicating that the ABF bias has converged sufficiently for residence-time analysis. All RTA results reported below were therefore obtained from this production interval.

**Figure 2:**
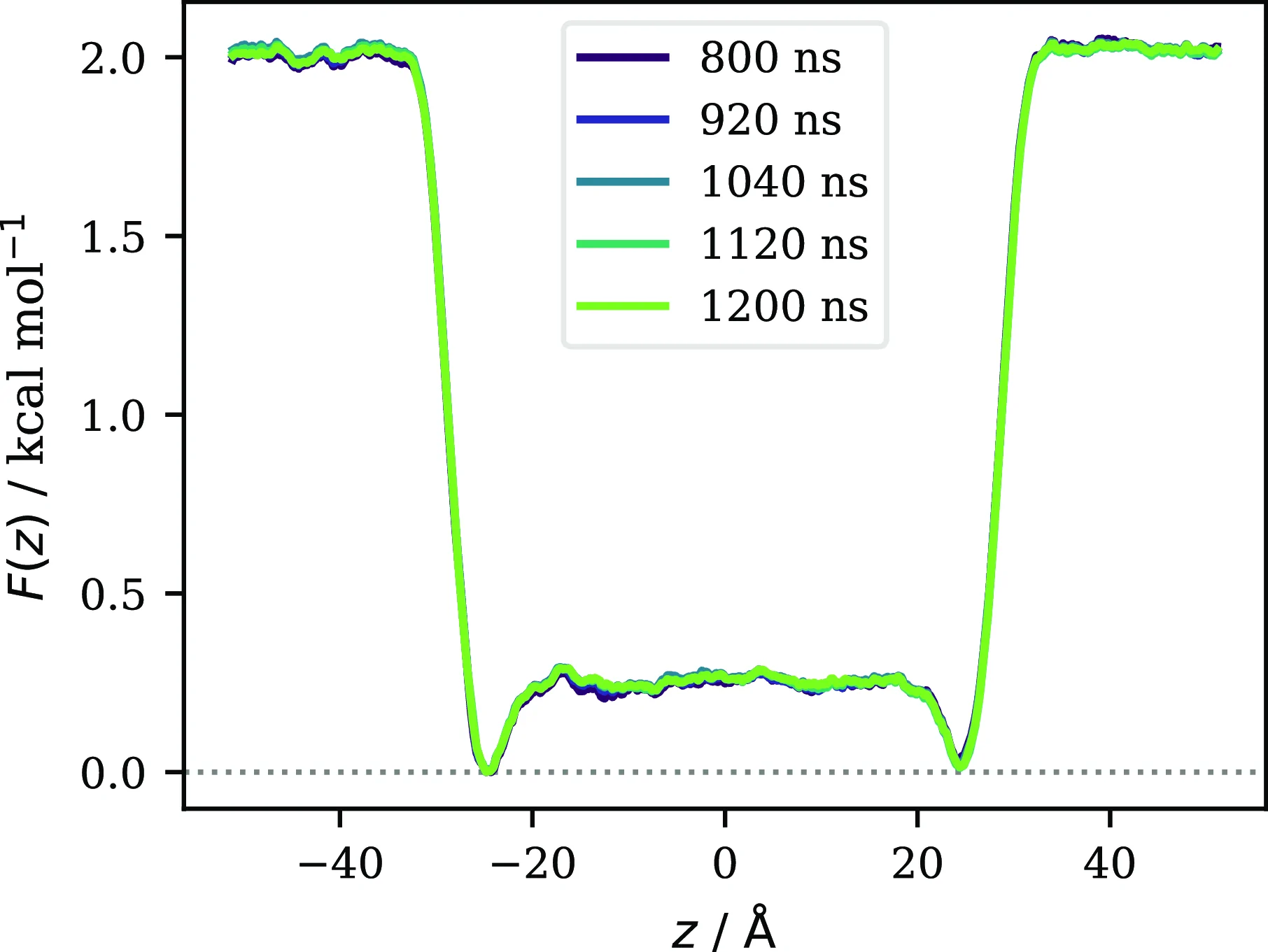
Convergence of PMF profiles from ABF simulations of oxygen diffusion across the hexadecane/water slab.

The free energy difference between the bulk water and hexadecane phases is consistent with the value reported by Ghysels et al.,^13^ indicating that the thermodynamic driving force for partitioning is well reproduced.

The diffusivity profile obtained from the RTA is shown in Figure 3, together with bulk reference values for water and hexadecane obtained from mean-squared-displacement (MSD) analysis (Section S1.3). Clear plateau regions are observed in both the aqueous and hydrophobic phases, consistent with approximately constant diffusivities in the bulk regions.

**Figure 3:**
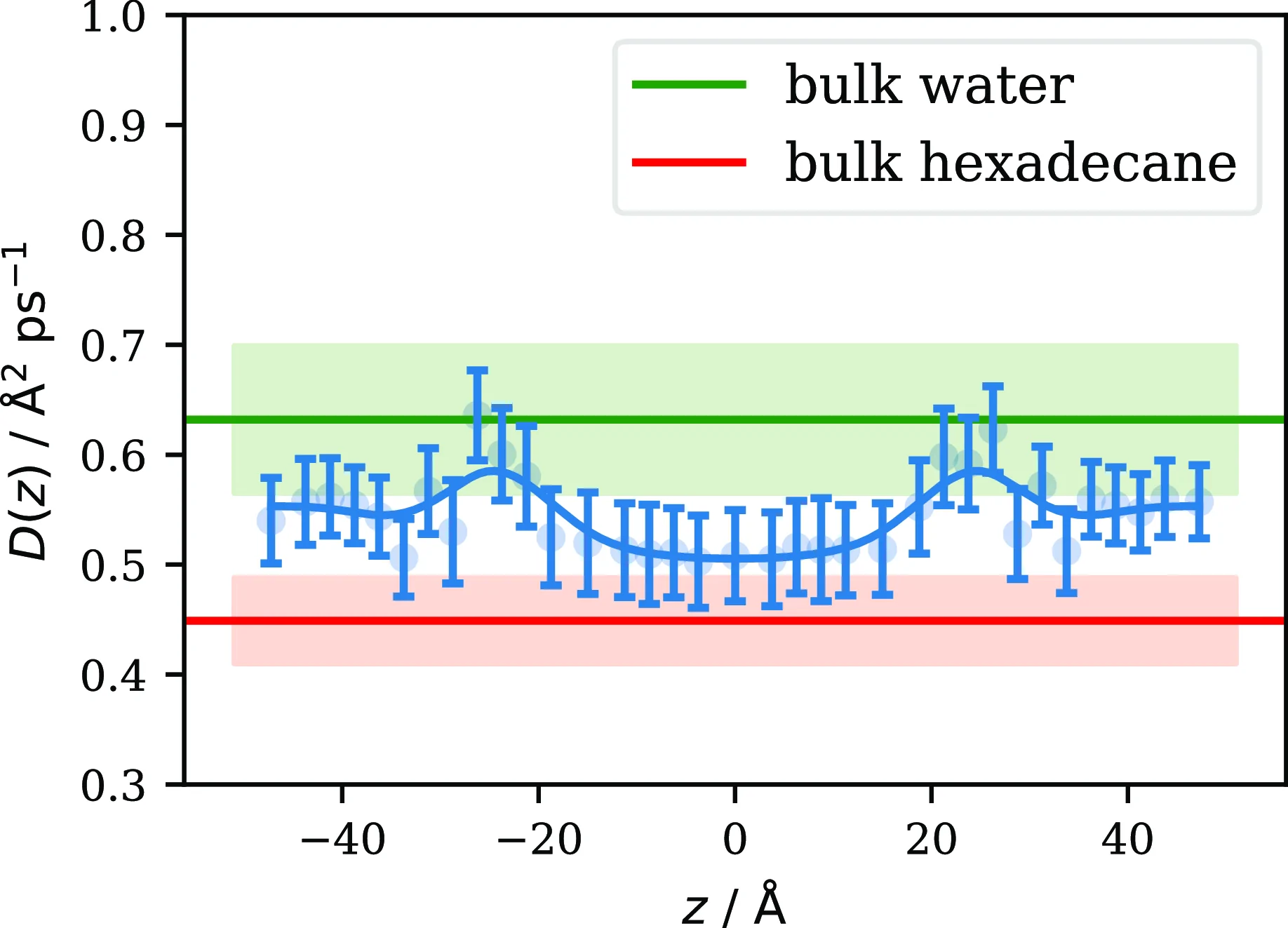
Diffusivity profiles for oxygen permeation across the hexadecane/water slab. Symbols denote computed diffusivities; lines are guides to the eye. The plotted diffusivities were obtained by averaging over 10 individually tracked oxygen molecules. Details of the bulk diffusivity calculations and individual profile data are provided in Sections S1.3 and S1.6.

The RTA-derived bulk diffusivities are statistically compatible with the MSD reference values. Small systematic deviations are observed, but the corresponding uncertainty intervals overlap, and the absolute differences are below 0.1 Å^2^ ps^–1^. These discrepancies are small on the scale of the diffusivities considered here and are most likely attributable to the finite interval width used in the residence-time analysis. The agreement with independently determined bulk diffusivities therefore provides a direct benchmark for the RTA in a system where reliable reference values are accessible.

For comparison, we also analyzed the smaller hexadecane/water slab system described in Section S1.4, which more closely matches the setup of Ghysels et al.^13^ In that case, the reduced spatial extent of the bulk regions leads to less well-defined plateau behavior in the diffusivity profile, consistent with the expectation that broader bulk domains improve the robustness of local diffusivity estimates.

Overall, these results show that the RTA recovers the expected bulk limiting behavior and yields diffusivity profiles consistent with independently determined diffusion coefficients in both phases. The hexadecane/water slab therefore establishes a useful baseline before turning to membrane systems, in which validation relies on comparison with independent dynamical observables rather than on direct bulk references.

### 4.2 Water Permeation Across a POPC Lipid Bilayer

We next consider water permeation across a POPC lipid bilayer, a standard benchmark for transport in fluid-phase membranes. In contrast to the slab system, direct bulk reference diffusivities no longer provide a meaningful test of the full position-dependent diffusivity profile through the membrane interior. We therefore compare the RTA with established fluctuation-based estimators and subsequently assess the resulting diffusivity profiles through propagator-level comparison with unbiased MD simulations.

The validity of the RTA again requires trajectory segments for which the effective PMF is approximately flat. Convergence of the ABF bias was assessed from the time evolution of the PMF (Figure 4). From 520 ns onward, the PMF remains stationary and symmetric within statistical uncertainty, indicating that the bias has converged sufficiently for residence-time analysis. The final ABF-derived PMF is also in good agreement with the independent US estimate of the same underlying free-energy profile reported in ref 32.

**Figure 4:**
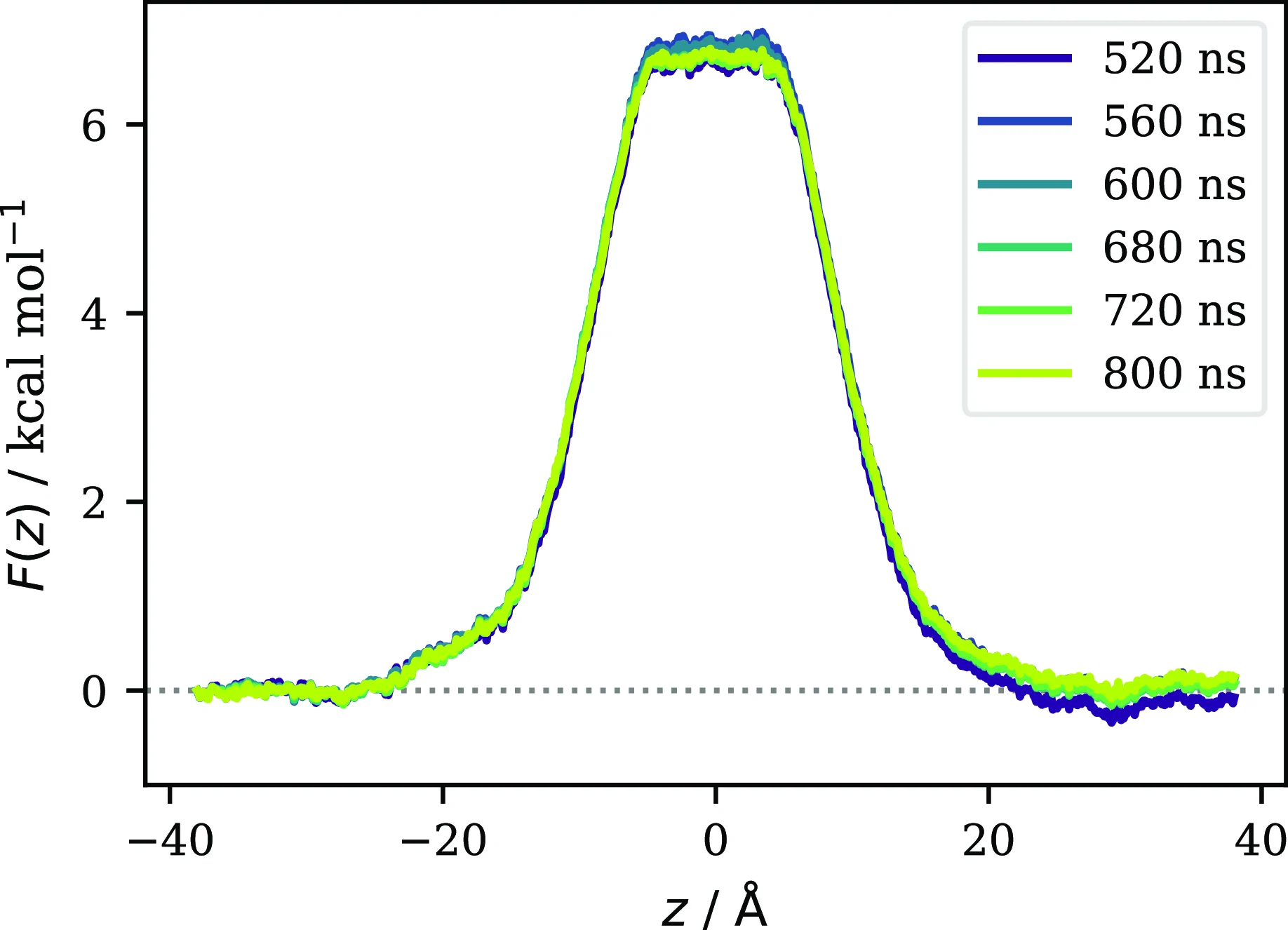
Convergence of PMF profiles from ABF simulations of water permeation across a POPC bilayer.

The diffusivity profile obtained from the RTA is shown in Figure 5, together with profiles obtained using the VACF and PACF approaches.^37^ All three methods yield similar diffusivities in the bulk water, whereas larger differences emerge in the bilayer interior. In particular, the RTA-derived diffusivity at the bilayer center lies between the corresponding VACF and PACF estimates.

**Figure 5:**
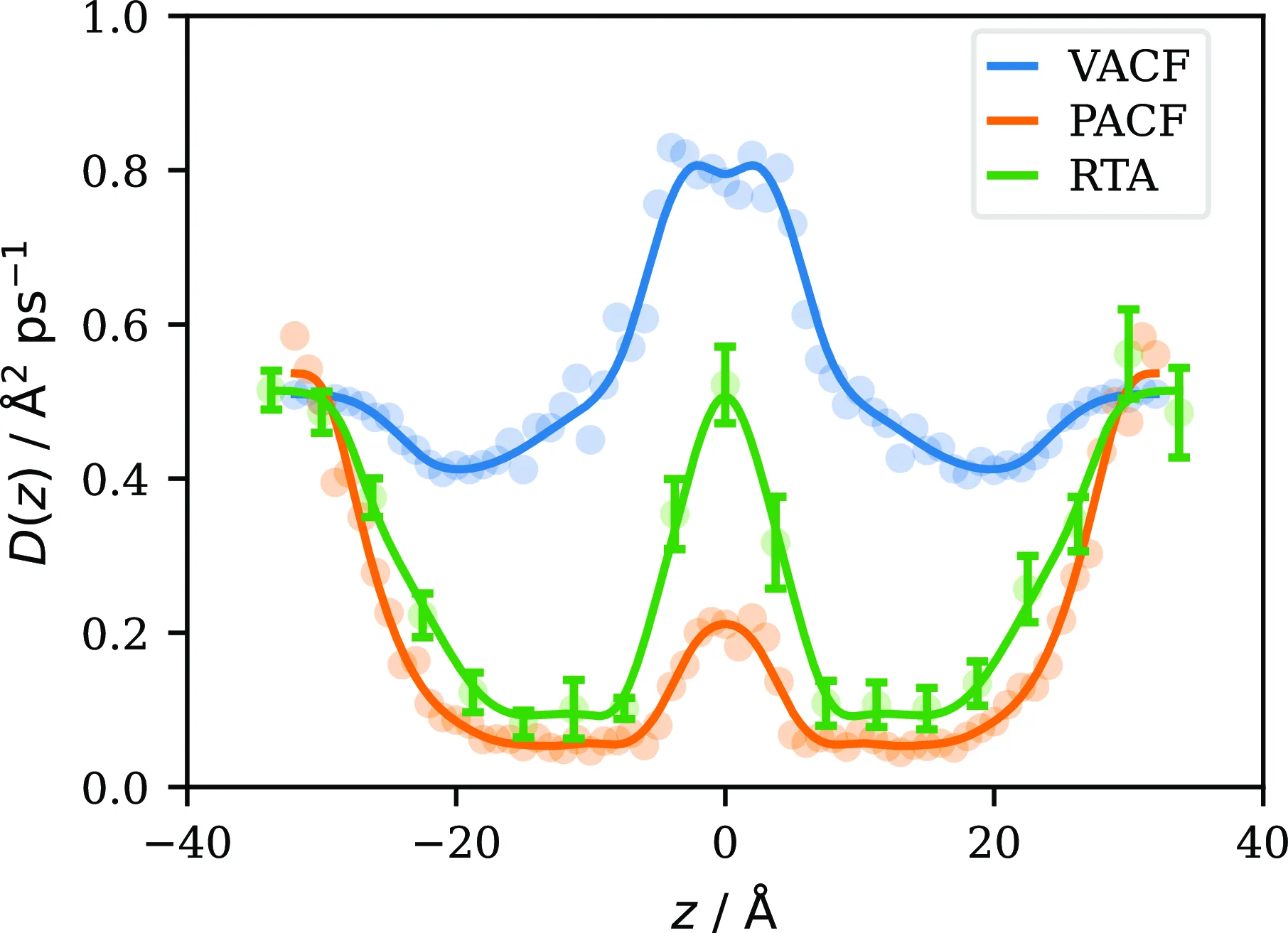
Diffusivity profiles across the POPC lipid bilayer obtained from the VACF, PACF, and residence-time approaches (RTA). Symbols denote the computed diffusivities, and solid lines show the smoothing-spline representations used for the propagator calculations.

The substantial differences between the VACF- and PACF-derived diffusivities in the membrane interior have previously been reported for water permeation across lipid bilayers.^8,37^ Recent work has demonstrated that external confinement and constraints can modify the effective friction and memory kernel experienced by a solute, leading to diffusivities that differ systematically from those obtained for freely diffusing particles.^21,22^ Motivated by these observations, we conjecture that the discrepancies between the VACF and PACF estimators may arise from confinement-induced memory effects, spatial variations of the local diffusivity within the biased region, or coupling to slow solvent and membrane relaxation processes. While this interpretation remains speculative, it suggests that agreement between different equilibrium estimators cannot by itself establish the validity of a reduced diffusive description.

To assess the resulting reduced descriptions, we compared model propagators obtained by solving the Smoluchowski eq 1 with propagators derived directly from unbiased MD simulations.^37^ This propagator-level comparison is particularly sensitive to possible errors arising from confinement-induced changes in effective friction or memory, which may affect different diffusivity estimators in different ways.^21,22^

We focus on propagators originating from *z*_0_ = 0, corresponding to the membrane center, where the largest differences between the diffusivity profiles are observed (Figure 5). Figure 6 compares predictions obtained with each diffusivity profile against the MD-derived propagators. Results are shown for representative lag times of 75 and 200 ps, which lie well within the diffusive regime. Repeating the comparison with the US-derived PMF estimate does not change the relative performance of the three diffusivity profiles (Section S2.3).

**Figure 6:**
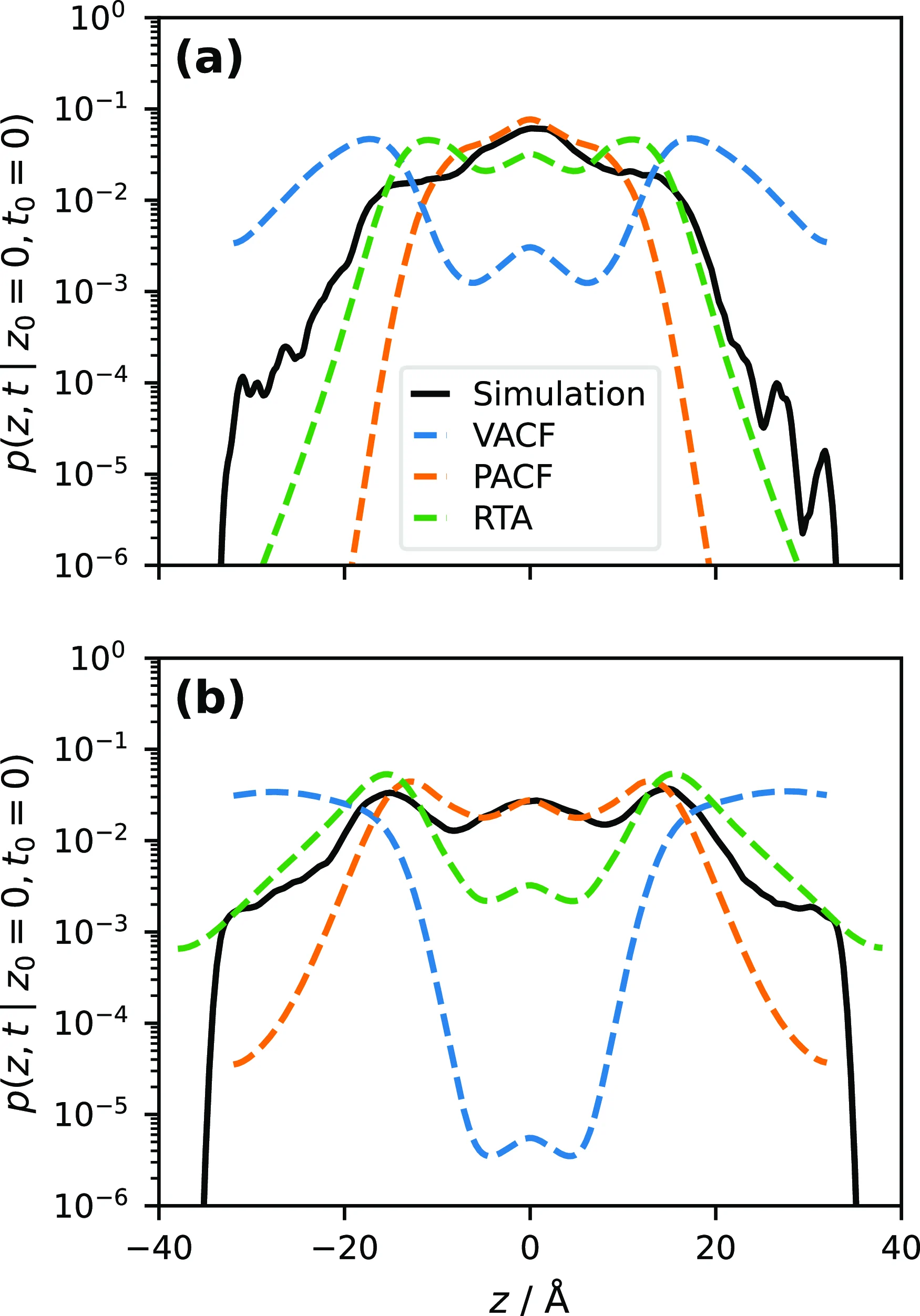
Comparison of MD-derived propagators with model predictions obtained using diffusivity profiles from the VACF, PACF, and residence-time approaches (RTA) for water permeation across the POPC bilayer. Propagators originate from *z*_0_ = 0, corresponding to the membrane center. Results are shown for lag times of (a) 75 ps and (b) 200 ps.

All three diffusivity profiles reproduce the overall broadening of the distributions with increasing lag time. At very short lag times up to about 10 ps, the VACF-derived diffusivity yields the closest agreement with the MD-derived propagators. However, this regime is not expected to satisfy the diffusive, Markovian assumptions underlying a Smoluchowski description. At the representative lag times shown here, the RTA-based diffusivity yields the closest agreement with the MD-derived propagators in the membrane center, whereas the VACF- and PACF-based diffusivities show more pronounced deviations.

Over an extended range of lag times (Section S2.3), the VACF-derived diffusivity systematically overestimates the diffusive spread. Neither the PACF- nor the RTA-derived diffusivity reproduces the MD propagators over the entire lag-time range considered, indicating that no single lag-time-independent diffusivity profile fully captures the projected dynamics in POPC. Nevertheless, the model based on the RTA-derived diffusivity remains in close agreement with the unbiased MD propagators over a substantial range of lag times extending to approximately 200 ps. Notably, this time scale considerably exceeds the mean residence time within the interval used to estimate the diffusivity, demonstrating that the effective diffusivity inferred from local first-exit statistics remains predictive well beyond the temporal scale from which it is derived. At the largest lag times considered, the PACF-derived diffusivity provides somewhat better agreement with the MD propagators. The observed crossover further suggests that no single Markovian diffusion model captures the projected dynamics across all time scales, consistent with previously reported subdiffusive behavior for small-molecule permeation across POPC bilayers.^23^

More generally, the agreement between the RTA-derived propagators and unbiased MD supports the spatial coarse-graining implied by the chosen interval width *L* = 7.5 Å, indicating that the dynamics are well described by diffusion with locally constant drift and diffusivity on this length scale. As discussed further below and in Section S5.3, the estimated diffusivities exhibit a plateau over a finite range of *L*, indicating that the reported results are not strongly affected by the precise choice of interval width within this regime.

### 4.3 Water and VOC Permeation Across the SC Lipid Membrane

The SC membrane provides a substantially more demanding test of the RTA than POPC because of its highly ordered, multicomponent lipid organization. Unlike fluid-phase phospholipid bilayers, the SC lipid matrix consists of a heterogeneous mixture of ceramides, free fatty acids, and cholesterol that exhibits reduced molecular mobility and slow structural relaxation.^32^ These characteristics give rise to heterogeneous transport pathways, thereby challenging reduced one-dimensional diffusion models and rendering free-energy calculations exceptionally difficult to converge.^32,37^

To account for these slow relaxation processes, we employed a windowed ABF sampling scheme along the CV. The convergence behavior in each window is analyzed in detail in Section S3.2, and the RTA was applied only to trajectory segments from the converged portions of the individual windows. Comparison with independent US-derived PMF estimates (Section S3.3) shows close agreement for water, whereas noticeable differences remain for the more complex organic solutes.^32^ Because permeability weights the barrier regions exponentially, these residual differences can produce substantial differences in quantitative permeability estimates, as examined below. In the propagator analysis, we hold the ABF-derived PMF fixed across the three diffusivity profiles and assess the resulting reduced Smoluchowski models against unbiased MD simulations. Corresponding calculations using the US-derived PMF estimate are reported in Section S3.4.

The diffusivity profiles obtained from the RTA are shown in Figure 7. For water, the RTA-derived diffusivity follows the same qualitative trend observed in POPC, lying between the VACF and PACF estimates in the membrane center. For the organic solutes, acetone and 6-MHO, the RTA and PACF profiles are in close agreement throughout both the membrane and bulk water regions, whereas the VACF approach yields systematically different profiles, consistent with the trends already observed in POPC.

**Figure 7:**
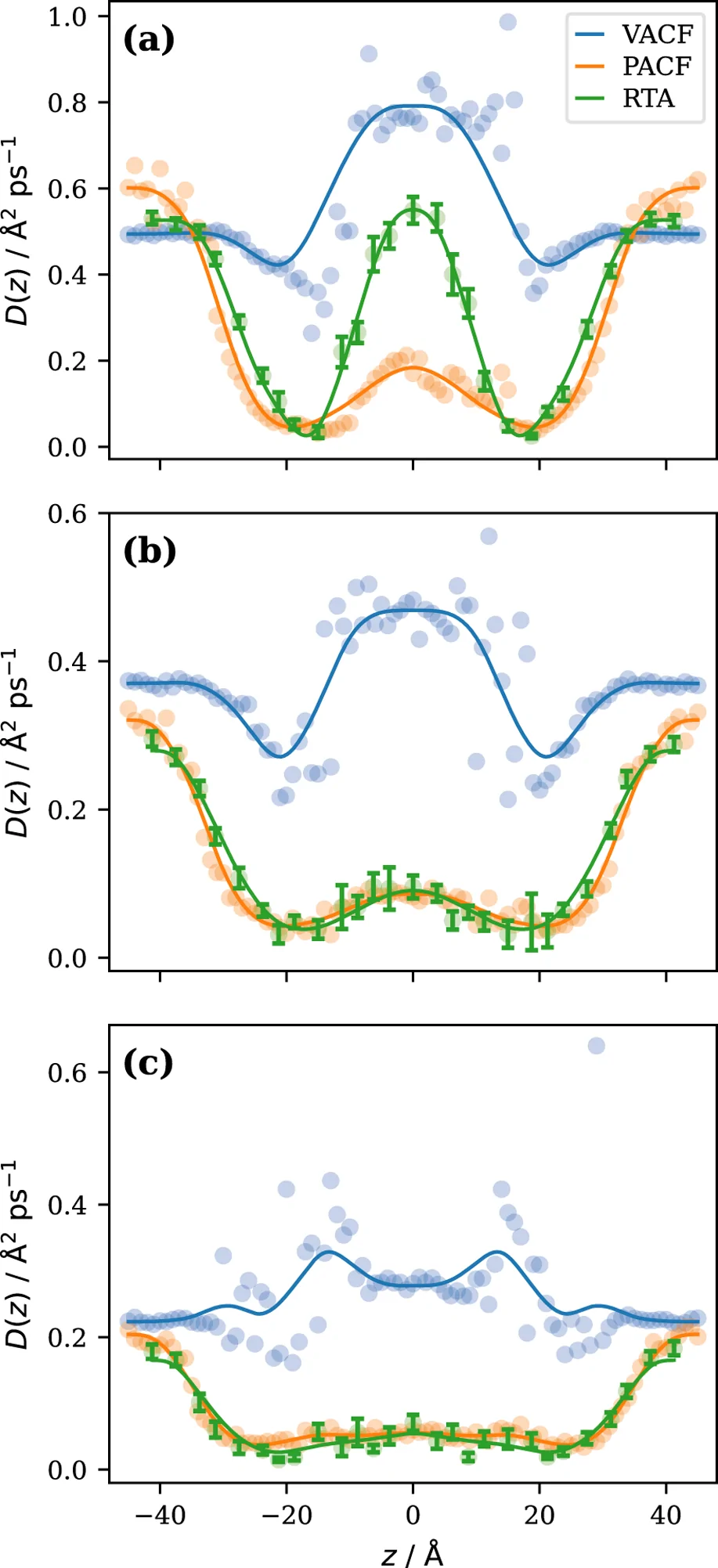
Position-dependent diffusivity profiles across the SC membrane for (a) water, (b) acetone, and (c) 6-MHO. Symbols denote the computed diffusivities, and solid lines show the smoothing-spline representations used for the propagator calculations.

We applied the same propagator-level validation to the SC membrane. Figure 8 shows representative results for water at lag times of 75 and 200 ps. In the membrane center, the RTA-derived diffusivity yields the closest agreement with the MD-derived propagators. In contrast to POPC, this agreement persists over the full range of lag times considered, up to 700 ps (Section S3.4). The relative performance of the three diffusivity profiles is unchanged when the US-derived PMF estimate is used instead of the ABF-derived estimate.

**Figure 8:**
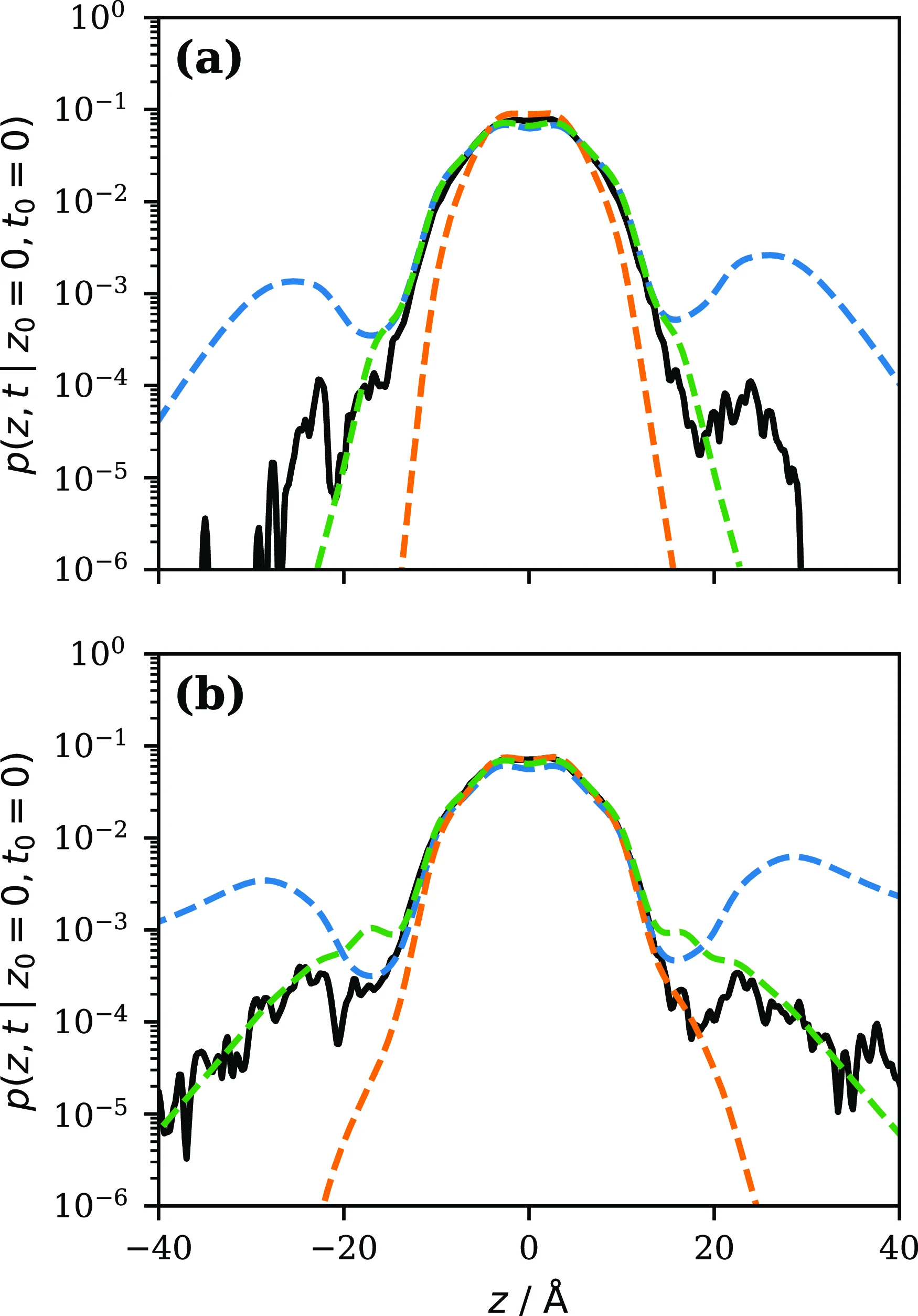
Comparison of MD-derived propagators with model predictions for water permeation across the SC membrane, obtained using diffusivity profiles from the VACF, PACF, and residence-time approaches (RTA). The common ABF-derived PMF was used for all model predictions. Propagators originate from *z*_0_ = 0, corresponding to the membrane center. Results are shown for lag times of (a) 75 ps and (b) 200 ps.

A similar pattern is observed for the organic solutes. For acetone and 6-MHO, the RTA and PACF approaches perform comparably, consistent with the near-identical diffusivity profiles obtained from these two methods, whereas VACF-based propagators again predict systematically broader spreading.

Taken together, these results indicate that the RTA can provide an effective reduced description of projected transport in the SC membrane despite its structural heterogeneity and slow relaxation processes. In this system, the residence-time estimator performs well for all solutes examined and, for water, yields the closest propagator-level agreement among the methods compared here.

### 4.4 Permeabilities

To separate the effects of the PMF and diffusivity profile, we calculated permeabilities using every combination of the ABF- and US-derived PMF estimates with the RTA-, PACF-, and VACF-derived diffusivities. The results are shown in Figure 9, with numerical values reported in Section S4.2.

**Figure 9:**
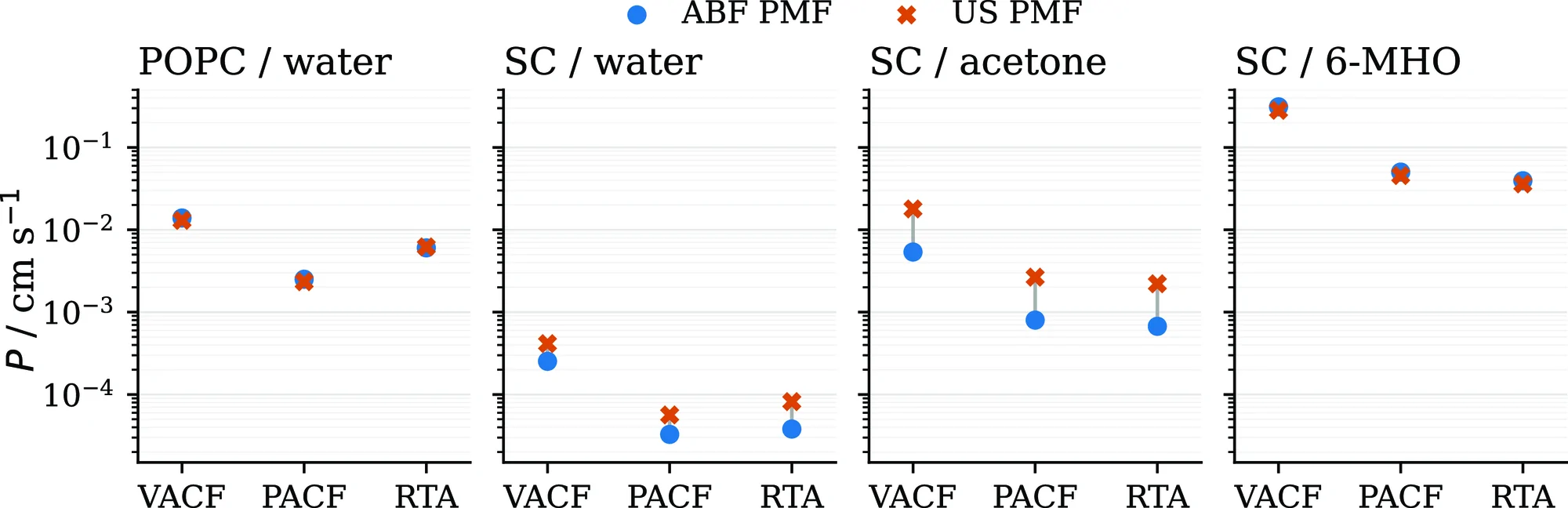
Sensitivity of the calculated permeability coefficients to the PMF estimate and diffusivity profile used in the inhomogeneous solubility–diffusion model. For each system and diffusivity estimator, permeabilities were calculated using both the ABF- and US-derived PMF estimates. Points connected by a vertical line use the same diffusivity profile and differ only in the PMF estimate; the connecting lines indicate PMF sensitivity and do not represent statistical uncertainty.

The absolute permeabilities depend appreciably on the PMF estimate, particularly for the SC systems, where the ABF- and US-derived estimates differ in the barrier regions (Section S3.3). Nevertheless, the relative ordering of the three diffusivity estimators is unchanged by the choice of PMF estimate: the VACF-based estimates are consistently the largest, whereas the ordering of the RTA- and PACF-based estimates is system dependent. Thus, the qualitative comparison between diffusivity estimators is robust to the PMF estimate used, while accurate quantitative permeability prediction requires well-converged PMF and diffusivity profiles.

## 5 Conclusions and Outlook

We have introduced a residence-time approach (RTA) for determining position-dependent diffusivities from biased molecular simulations in the drift-free limit. The method uses trajectory segments for which the effective free-energy gradient along the transport coordinate is negligible and relates mean first-exit times from finite spatial intervals directly to effective local diffusivities. In the present work, this regime was realized using converged ABF simulations, but the formulation is not specific to ABF and can in principle be combined with other biasing strategies that generate an approximately flat effective free-energy landscape.

The RTA provides a practical alternative to fluctuation-based estimators based on harmonically restrained simulations. By extracting diffusivities from residence-time statistics in an approximately drift-free landscape, it avoids inferring transport coefficients from equilibrium fluctuations around an imposed restraint, a setting in which external confinement may alter the effective friction and memory kernel experienced by the solute.^21,22^ It also bypasses the correlation-function integrations required by conventional VACF- and PACF-based estimators.^3,10,11^

We evaluated the method from a simple liquid–liquid reference system with independently accessible bulk diffusivities to increasingly heterogeneous membrane systems where validation must rely on dynamical consistency. In the hexadecane/water system, where independent bulk reference diffusivities are available, the RTA reproduces the bulk diffusion coefficients in both phases within statistical uncertainty, providing a direct benchmark in a simple heterogeneous reference system.

For the membrane systems, direct bulk reference diffusivities are not available across the full transport coordinate. We therefore assessed the inferred diffusivity profiles by propagator-level validation against unbiased MD simulations. The primary comparison holds the ABF-derived PMF fixed across all diffusivity profiles, while calculations using the US-derived PMF estimate show that the relative ranking of the diffusivity profiles is unchanged.

In the POPC bilayer, the different diffusivity estimators provide the closest agreement with MD-derived propagators over different lag-time regimes. VACF-derived diffusivities perform best at very short lag times, although this regime lies outside the diffusive, Markovian regime that motivates a Smoluchowski description. At intermediate lag times, the RTA gives the closest agreement with the MD-derived propagators, whereas PACF-based diffusivities perform better at the longest lag times considered here. Taken together, these results indicate that the projected POPC dynamics cannot be represented over the full lag-time range by a single lag-time-independent one-dimensional Smoluchowski model. Instead, the apparent optimal diffusivity depends on the dynamical regime being probed, consistent with residual non-Markovianity, imperfect separation of slow membrane and solvent relaxation from solute motion, and limitations inherent to projecting the full dynamics onto a single membrane-normal coordinate.

In the more ordered and heterogeneous SC membrane, the RTA performs well across all solutes examined. For acetone and 6-MHO, the RTA and PACF diffusivities are very similar throughout the membrane, whereas VACF-based estimates again predict systematically broader propagators. For water, the RTA provides the most accurate propagator-level description among the methods compared here.

Permeabilities computed from all combinations of the two PMF estimates and three diffusivity profiles separate the kinetic contribution from the sensitivity to the thermodynamic input. The relative ordering associated with the diffusivity estimators is unchanged upon substituting the US-derived PMF estimate for the ABF-derived estimate, but the absolute permeability coefficients can change appreciably, particularly for the SC systems. The membrane results therefore reinforce two main conclusions: first, that the RTA yields diffusivities comparable to those from established estimators and, in several cases, gives closer agreement with MD-derived propagators among the methods compared here; and second, that quantitative permeability prediction is highly sensitive to convergence of both the kinetic and thermodynamic inputs.

Taken together, these results support residence-time statistics as a practical route for extracting position-dependent diffusivities from biased MD simulations. At the same time, the remaining differences between model and simulation propagators indicate that the inferred diffusivities should not be interpreted as uniquely exact local transport coefficients. Rather, they should be viewed as components of an effective reduced stochastic model, whose validity is best assessed by its ability to reproduce independent dynamical observables such as propagators.

An important conclusion of the present work concerns the role of the interval width *L*. Rather than being a purely numerical parameter, *L* should be regarded as the intrinsic spatial coarse-graining length of the RTA. In this respect, it plays a role analogous to the lag time used when extracting diffusion coefficients from mean-squared displacement analyses: no universal optimal choice can be expected, and instead one seeks an intermediate regime in which the assumptions underlying diffusive, Markovian dynamics are satisfied while preserving sufficient spatial resolution. For the systems considered here, the analysis presented in Section S5.3 reveals a clear plateau in the estimated diffusivities over a finite range of *L*, encompassing the value used throughout this work. Together with the propagator analysis, this supports the chosen coarse-graining as a robust reduced description.

The dependence of the estimated diffusivity on *L* reflects that residence-time statistics are evaluated over finite spatial intervals. This is analogous in spirit to the finite-window effects encountered in local displacement-based estimators, for which form-factor correction schemes have recently been proposed.^16,17^ Although the present work does not derive an analogous correction for first-exit statistics, this connection suggests a useful direction for future developments of the RTA. A systematic investigation of the dependence on *L* across a broader range of systems, including those exhibiting pronounced non-Markovian or anomalous dynamics,^23^ could establish best-practice guidelines for selecting *L* and may ultimately enable mechanistically motivated correction schemes for finite interval widths.

A second open question concerns the contrast between the POPC and SC results. Although POPC is structurally more disordered than SC, it appears to be more difficult to describe using a single lag-time-independent diffusivity profile. Determining whether this difference reflects stronger memory effects, different coupling of the chosen coordinate to orthogonal degrees of freedom, or differences in local structural relaxation will help define the range of validity of reduced one-dimensional diffusion models for membrane transport.

A third question concerns the distinct behavior of water. In both membrane systems, water shows larger differences between RTA- and PACF-derived diffusivities than the organic solutes, whereas for acetone and 6-MHO the two approaches yield closely similar results. This suggests that water is particularly sensitive to dynamical effects that are captured differently by diffusivity estimators, possibly because of its small size, hydrogen-bonding capability, and coupling to local membrane fluctuations. A broader comparison across solute classes may therefore help identify when different estimators converge to the same effective description and when they do not.

## Supplementary Material



## Data Availability

Initial structures, simulation input files, and the software library implementing the residence-time approach are available on GitHub at https://github.com/mvondomaros-lab/rta-paper-data and https://github.com/mvondomaros-lab/pdda, respectively. The pdda library includes the implementation of the residence-time diffusivity estimator, the associated uncertainty quantification procedures, the smoothing-spline interpolation of discrete diffusivity profiles, and the numerical propagator analysis described in this work. Additional data supporting the findings of this study are available from the corresponding author upon reasonable request.

## Notes

Additional analyses of the hexadecane/water slab systems, including system definitions, orientational distributions, bulk reference diffusion coefficients, residence-time analysis of the smaller slab system, and supplementary PMF and diffusivity profiles; supplementary POPC analyses, including PMF evolution before bias convergence, comparison of ABF- and umbrella-sampling-derived PMF estimates, and propagator sensitivity to the PMF estimate; supplementary stratum corneum analyses, including ABF window definitions, window-wise PMF convergence, comparison of PMF estimates from different sampling methods, and propagator sensitivity to the PMF estimate; supplementary permeability analyses, including the definition of integration bounds and the full set of permeability coefficients; and methodological validation of the residence-time approach, including local dynamical statistics, uncertainty quantification for correlated residence-time data, and dependence on the residence interval width (PDF)

## References

[1] Einstein A. (1905). Über
die von der molekularkinetischen Theorie der Wärme geforderte
Bewegung von in ruhenden Flüssigkeiten suspendierten Teilchen. Ann. Phys..

[2] Kubo R. (1957). Statistical-mechanical
theory of irreversible processes. I. General theory and simple applications
to magnetic and conduction problems. J. Phys.
Soc. Jpn..

[3] Hummer G. (2005). Position-dependent
diffusion coefficients and free energies from Bayesian analysis of
equilibrium and replica molecular dynamics simulations. New J. Phys..

[4] Risken, H. The Fokker-Planck Equation: Methods of Solution and Applications; Springer, 1996.

[5] Diamond J.
M., Katz Y. (1974). Interpretation
of nonelectrolyte partition coefficients between dimyristoyl
lecithin and water. J. Membr. Biol..

[6] Venable R. M., Krämer A., Pastor R. W. (2019). Molecular dynamics simulations of
membrane permeability. Chem. Rev..

[7] Lee C. T., Comer J., Herndon C., Leung N., Pavlova A., Swift R. V., Tung C., Rowley C. N., Amaro R. E., Chipot C., Wang Y., Gumbart J. C. (2016). Simulation-based
approaches for determining membrane permeability of small compounds. J. Chem. Inf. Model..

[8] Awoonor-Williams E., Rowley C. N. (2016). Molecular simulation
of nonfacilitated membrane permeation. Biochim.
Biophys. Acta, Biomembr..

[9] Shinoda W. (2016). Permeability
across lipid membranes. Biochim. Biophys. Acta,
Biomembr..

[10] Woolf T. B., Roux B. (1994). Conformational flexibility
of O-phosphorylcholine and O-phosphorylethanolamine:
A molecular dynamics study of solvation effects. J. Am. Chem. Soc..

[11] Gaalswyk K., Awoonor-Williams E., Rowley C. N. (2016). Generalized Langevin methods for
calculating transmembrane diffusivity. J. Chem.
Theory Comput..

[12] Straub J. E., Borkovec M., Berne B. J. (1987). Calculation
of dynamic friction on
intramolecular degrees of freedom. J. Phys.
Chem..

[13] Ghysels A., Venable R. M., Pastor R. W., Hummer G. (2017). Position-dependent
diffusion tensors in anisotropic media from simulation: Oxygen transport
in and through membranes. J. Chem. Theory Comput..

[14] Krämer A., Ghysels A., Wang E., Venable R. M., Klauda J. B., Brooks B. R., Pastor R. W. (2020). Membrane permeability
of small molecules
from unbiased molecular dynamics simulations. J. Chem. Phys..

[15] Comer J., Chipot C., González-Nilo F. D. (2013). Calculating
position-dependent
diffusivity in biased molecular dynamics simulations. J. Chem. Theory Comput..

[16] Nagai T., Tsurumaki S., Urano R., Fujimoto K., Shinoda W., Okazaki S. (2020). Position-Dependent
Diffusion Constant of Molecules
in Heterogeneous Systems as Evaluated by the Local Mean Squared Displacement. J. Chem. Theory Comput..

[17] Kikkawa N., Jinnouchi R., Nagai T., Okazaki S., Kimura M. (2026). Local Diffusion
Analysis Using Square Displacement Averaged in Subspace. J. Chem. Phys..

[18] Rosta E., Hummer G. (2015). Free energies from
dynamic weighted histogram analysis
using unbiased Markov state model. J. Chem.
Theory Comput..

[19] Stelzl L. S., Kells A., Rosta E., Hummer G. (2017). Dynamic histogram analysis
to determine free energies and rates from biased simulations. J. Chem. Theory Comput..

[20] Sicard F., Koskin V., Annibale A., Rosta E. (2021). Position-dependent
diffusion from biased simulations and Markov state model analysis. J. Chem. Theory Comput..

[21] Daldrop J. O., Kowalik B. G., Netz R. R. (2017). External Potential Modifies Friction
of Molecular Solutes in Water. Phys. Rev. X.

[22] Yamaguchi T. (2021). Decoupling
between Solvent Viscosity and Diffusion of a Small Solute Induced
by Self-Motion. J. Phys. Chem. Lett..

[23] Chipot C., Comer J. (2016). Subdiffusion in membrane
permeation of small molecules. Sci. Rep..

[24] Ghysels A., Roet S., Davoudi S., Van Erp T. S. (2021). Exact non-Markovian
permeability from rare event simulations. Phys.
Rev. Res..

[25] Vervust W., Zhang D. T., Van Erp T. S., Ghysels A. (2023). Path sampling with
memory reduction and replica exchange to reach long permeation timescales. Biophys. J..

[26] Darve E., Pohorille A. (2001). Calculating
free energies using average force. J. Chem.
Phys..

[27] Comer J., Gumbart J. C., Hénin J., Lelièvre T., Pohorille A., Chipot C. (2015). The adaptive biasing
force method:
Everything you always wanted to know but were afraid to ask. J. Phys. Chem. B.

[28] Redner, S. A Guide to First-Passage Processes; Cambridge University Press, 2001.

[29] Gardiner, C. W. Stochastic Methods: A Handbook for the Natural and Social Sciences, 4 ed.; Springer, 2009; . th ed..

[30] Mercier
Franco L. F., Castier M., Economou I. G. (2016). Diffusion in homogeneous
and in inhomogeneous media: A new unified approach. J. Chem. Theory Comput..

[31] H”ollring K., Baer A., Vučemilović-Alagić N., Smith D. M., Smith A.-S. (2023). Anisotropic molecular diffusion in
confinement I: Transport of small particles in potential and density
gradients. J. Colloid Interface Sci..

[32] Thomas R., Prabhakar P. R., Tobias D. J., von Domaros M. (2025). Insights into
dermal permeation of skin oil oxidation products from enhanced sampling
molecular dynamics simulation. J. Phys. Chem.
B.

[33] Mathai J. C., Tristram-Nagle S., Nagle J. F., Zeidel M. L. (2008). Structural determinants
of water permeability through the lipid membrane. J. Gen. Physiol..

[34] Nagle J. F., Mathai J. C., Zeidel M. L., Tristram-Nagle S. (2008). Theory of
passive permeability through lipid bilayers. J. Gen. Physiol..

[35] Orsi M., Essex J. W. (2010). Permeability of
drugs and hormones through a lipid
bilayer: Insights from dual-resolution molecular dynamics. Soft Matter.

[36] Comer J., Schulten K., Chipot C. (2014). Diffusive
models of membrane permeation
with explicit orientational freedom. J. Chem.
Theory Comput..

[37] Thomas R., Prabhakar P. R., Tobias D. J., von Domaros M. (2026). Modeling diffusion
and permeation across the stratum corneum lipid barrier. ACS ES&T Air.

[38] Klauda J. B., Venable R. M., Freites J. A., O’Connor J. W., Tobias D. J., Mondragon-Ramirez C., Vorobyov I., MacKerell A. D., Pastor R. W. (2010). Update of the CHARMM
all-atom additive force field
for lipids: Validation on six lipid types. J.
Phys. Chem. B.

[39] Vanommeslaeghe K., Hatcher E., Acharya C., Kundu S., Zhong S., Shim J., Darian E., Guvench O., Lopes P., Vorobyov I., MacKerell A. D. (2010). CHARMM
general force field: A force
field for drug-like molecules compatible with the CHARMM all-atom
additive biological force fields. J. Comput.
Chem..

[40] Jorgensen W. L., Chandrasekhar J., Madura J. D., Impey R. W., Klein M. L. (1983). Comparison
of simple potential functions for simulating liquid water. J. Chem. Phys..

[41] Martínez L., Andrade R., Birgin E. G., Martínez J. M. (2009). PACKMOL:
A package for building initial configurations for molecular dynamics
simulations. J. Comput. Chem..

[42] Phillips J. C., Braun R., Wang W., Gumbart J., Tajkhorshid E., Villa E., Chipot C., Skeel R. D., Kalé L., Schulten K. (2005). Scalable molecular dynamics with NAMD. J. Comput. Chem..

[43] Phillips J.
C., Hardy D. J., Maia J. D. C., Stone J. E., Ribeiro J. V., Bernardi R. C., Buch R., Fiorin G., Hénin J., Jiang W., McGreevy R., Melo M. C. R., Radak B. K., Skeel R. D., Singharoy A., Wang Y., Roux B., Aksimentiev A., Luthey-Schulten Z., Kalé L. V., Schulten K., Chipot C., Tajkhorshid E. (2020). Scalable molecular
dynamics on CPU and GPU architectures with NAMD. J. Chem. Phys..

[44] Humphreys D. D., Friesner R. A., Berne B. J. (1994). A multiple-time-step
molecular dynamics
algorithm for macromolecules. J. Phys. Chem..

[45] Miyamoto S., Kollman P. A. (1992). SETTLE: An analytical version of
the SHAKE and RATTLE
algorithm for rigid water models. J. Comput.
Chem..

[46] Ryckaert J.-P., Ciccotti G., Berendsen H. J. C. (1977). Numerical
integration of the cartesian
equations of motion of a system with constraints: Molecular dynamics
of *n*-alkanes. J. Comput. Phys..

[47] Essmann U., Perera L., Berkowitz M. L., Darden T., Lee H., Pedersen L. G. (1995). A smooth particle
mesh Ewald method. J. Chem. Phys..

[48] Bussi G., Donadio D., Parrinello M. (2007). Canonical sampling through velocity
rescaling. J. Chem. Phys..

[49] Martyna G.
J., Tobias D. J., Klein M. L. (1994). Constant pressure molecular dynamics
algorithms. J. Chem. Phys..

[50] Feller S. E., Zhang Y., Pastor R. W., Brooks B. R. (1995). Constant pressure
molecular dynamics simulation: The Langevin piston method. J. Chem. Phys..

[51] Fiorin G., Klein M. L., Hénin J. (2013). Using Collective
Variables to Drive
Molecular Dynamics Simulations. Mol. Phys..

[52] Schow E. V., Freites J. A., Myint P. C., Bernsel A., von Heijne G., White S. H., Tobias D. J. (2011). Arginine
in membranes: The connection
between molecular dynamics simulations and translocon-mediated insertion
experiments. J. Membr. Biol..

[53] Flyvbjerg H., Petersen H. G. (1989). Error estimates on averages of correlated
data. J. Chem. Phys..

[54] Jonsson M. (2018). Standard error
estimation by an automated blocking method. Phys. Rev. E.

[55] McCluskey A. R., Coles S. W., Morgan B. J. (2025). Accurate
Estimation of Diffusion
Coefficients and Their Uncertainties from Computer Simulation. J. Chem. Theory Comput..

