# Supplementary material for: A Residence-Time Approach for Determining Position-Dependent Diffusivities from Biased Molecular Simulations
Source: J Chem Theory Comput. 2026 Aug 28;22(18):9235–46. doi: 10.1021/acs.jctc.6c00645 (PMC13602916; doi:10.1021/acs.jctc.6c00645)
Supplement: Supplementary file 1 [file ct6c00645_si_001.pdf]

Supporting Information: A Residence-Time Approach for Determining Position-Dependent Diffusivities from Biased Molecular Simulations

Rinto Thomas[†,¶], Praveen Ranganath Prabhakar[‡,¶], and Michael von Domaros[†,*]

[†] Fachbereich Chemie, Philipps-Universität Marburg, 35032 Marburg, Germany
[‡] Department of Chemistry, University of California, Irvine, Irvine, California 92697, United States
[¶]R.T. and P.R.P. contributed equally to this work.

* mvondomaros@uni-marburg.de

S1 O_2 in the Hexadecane/Water Slab

S1.1 Hexadecane/Water Slab Systems

In addition to the hexadecane/water slab system analyzed in the main text, hereafter denoted HS-II, we investigated a smaller system, HS-I, with approximately half the box length in the z direction. HS-I corresponds to the smaller slab system studied previously by Ghysels *et al.*[1]

The system compositions and box dimensions are listed in Table S1. Density profiles of the two slab systems are shown in Figure S1.

Table S1: Compositions of the hexadecane/water slab systems and the corresponding simulation box dimensions.

System	Hexadecane	Water	O_2	L_x (Å)	L_y (Å)	L_z (Å)
HS-I	126	2159	10	50.00	50.00	53.20
HS-II	252	4318	10	50.00	50.00	105.00

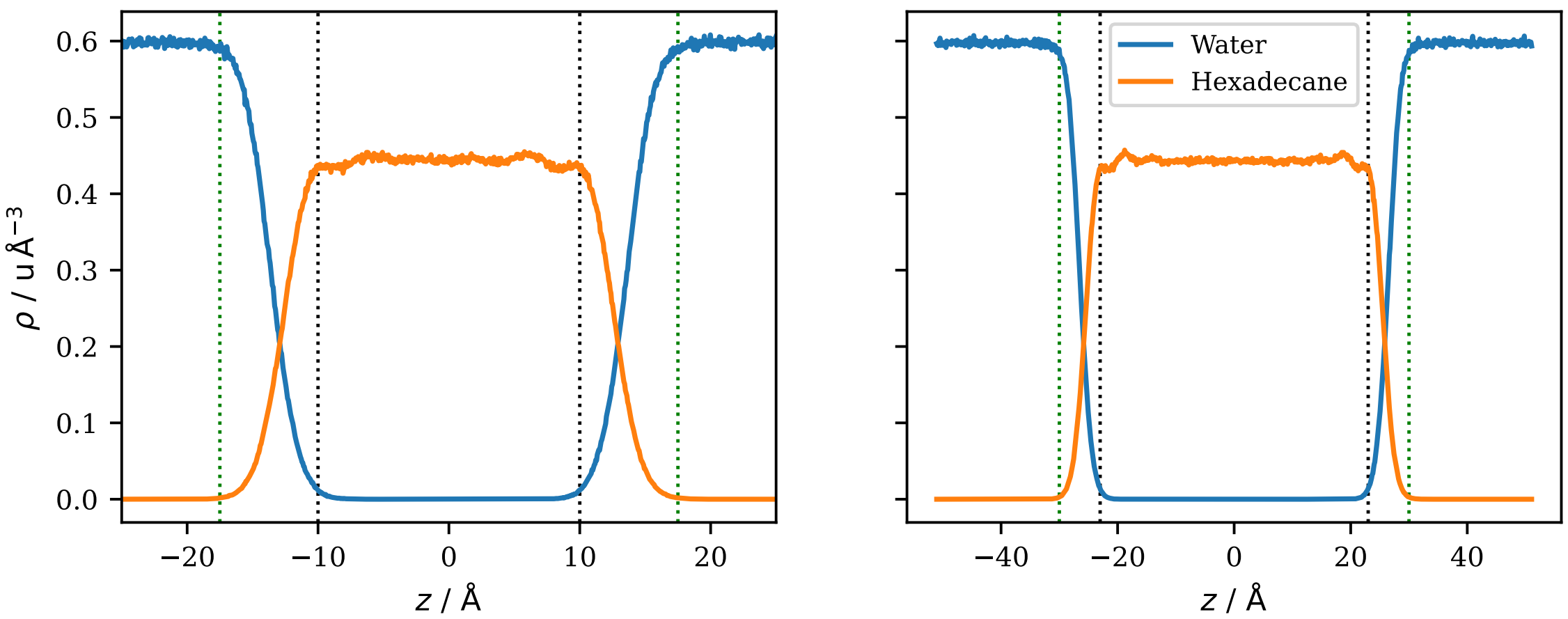

Figure S1: Density profiles of the two hexadecane/water slab systems considered in this work. The profiles were obtained from an unbiased 40 ns production run. The black and green vertical dashed lines indicate the boundaries of the bulk-like regions of hexadecane and water, respectively.

S1.2 Orientational Distributions

In this section, we characterize molecular orientations in the hexadecane/water slab relative to the z-axis to assess the extent of bulk-like behavior in HS-I and HS-II. The larger slab system, HS-II, was selected for the main-text analysis because, unlike HS-I, it exhibits extended regions consistent with bulk-like behavior in both phases.

For hexadecane, we defined the orientation angle, θ_{HEXD}, as the angle between the z-axis and the vector connecting the first and terminal carbon atoms of an aliphatic chain (Figure S2). For water, we defined the orientation angle, θ_{W}, as the angle between the z-axis and the molecular C_2 axis, defined here as the vector connecting the oxygen atom and the midpoint between the two hydrogen atoms (Figure S2). The orientational distributions are expressed in terms of $\cos\theta_{\mathrm{HEXD}}$ and $\cos\theta_{\mathrm{W}}$ as functions of position along the z-axis (Figures S3–S6). These distributions were computed from an unbiased 40 ns production run. The distributions were symmetrized with respect to the slab center. For hexadecane, we used $p(|\cos\theta_{\mathrm{HEXD}}|, z) = p(|\cos\theta_{\mathrm{HEXD}}|, -z)$, reflecting the apolar, axis-like character of the molecular orientation. For water, we used $p(\cos\theta_{\mathrm{W}}, z) = p(-\cos\theta_{\mathrm{W}}, -z)$, reflecting the inversion symmetry of the two interfaces.

In contrast to HS-I, HS-II exhibits extended regions with only weak variation in the orientational distributions in both the hexadecane phase (Figures S3 and S4) and the water phase (Figures S5 and S6), consistent with the presence of bulk-like domains.

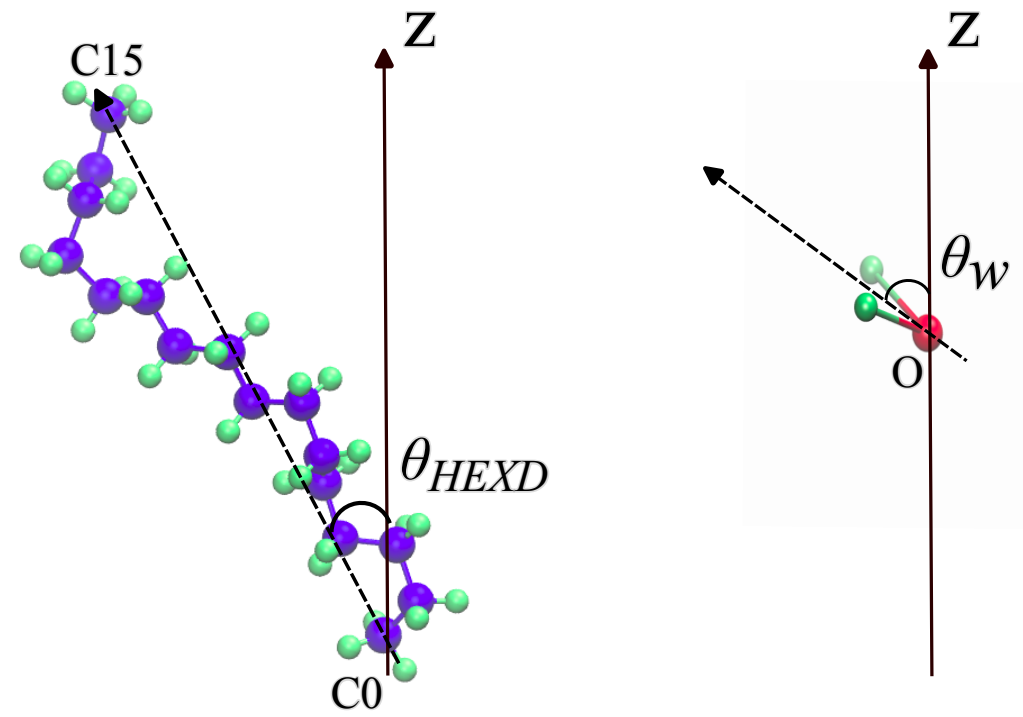

Figure S2: Schematic definition of the orientation angles used in the orientational analysis. Left panel: hexadecane; right panel: water. Carbon, hydrogen, and oxygen atoms are shown in blue, green, and red, respectively. The dotted line denotes the molecular orientation vector, and the vertical dashed line denotes the z-axis.

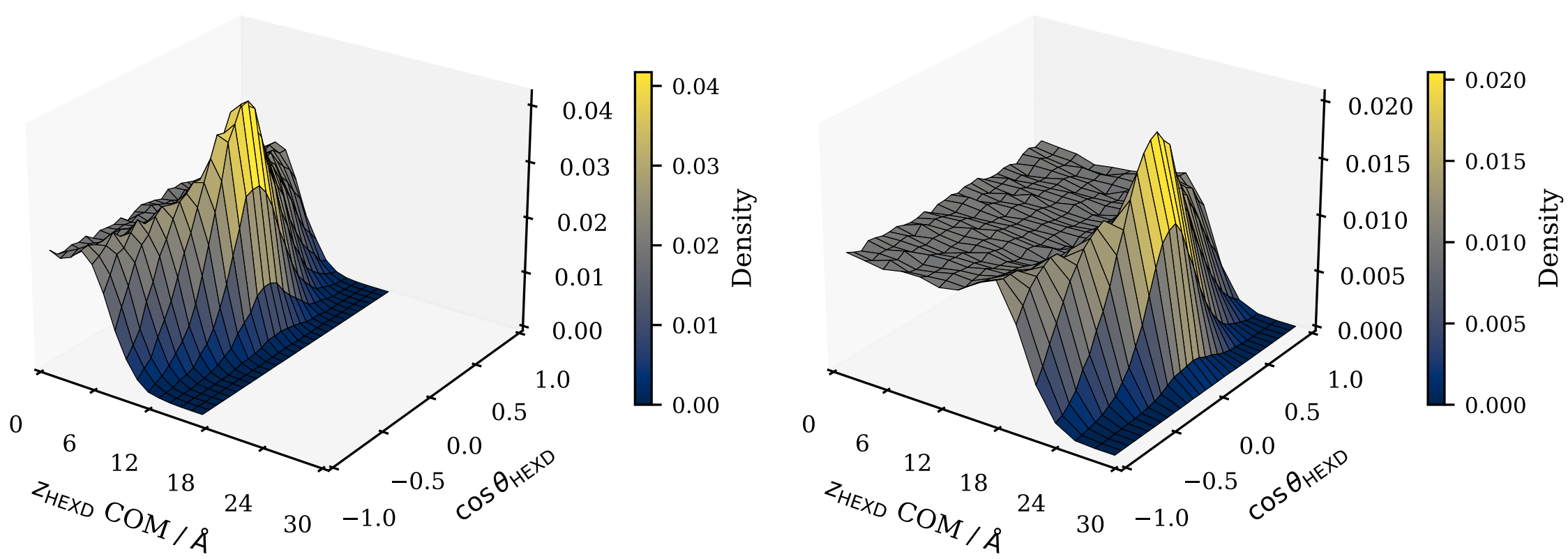

Figure S3: Three-dimensional distributions of $\cos\theta_{\mathrm{HEXD}}$ as a function of the hexadecane center-of-mass position, z_{HEXD}, for HS-I (left) and HS-II (right).

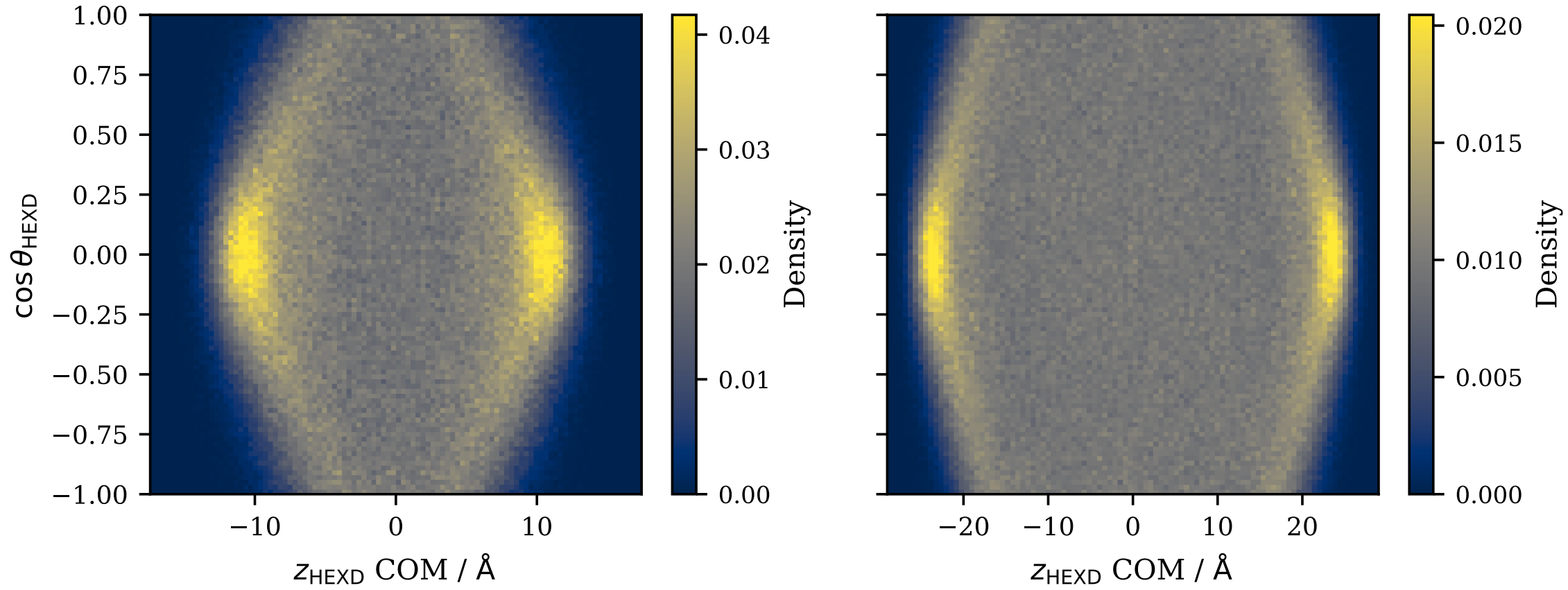

Figure S4: Two-dimensional distributions of $\cos\theta_{\mathrm{HEXD}}$ as a function of the hexadecane center-of-mass position, z_{HEXD}, for HS-I (left) and HS-II (right).

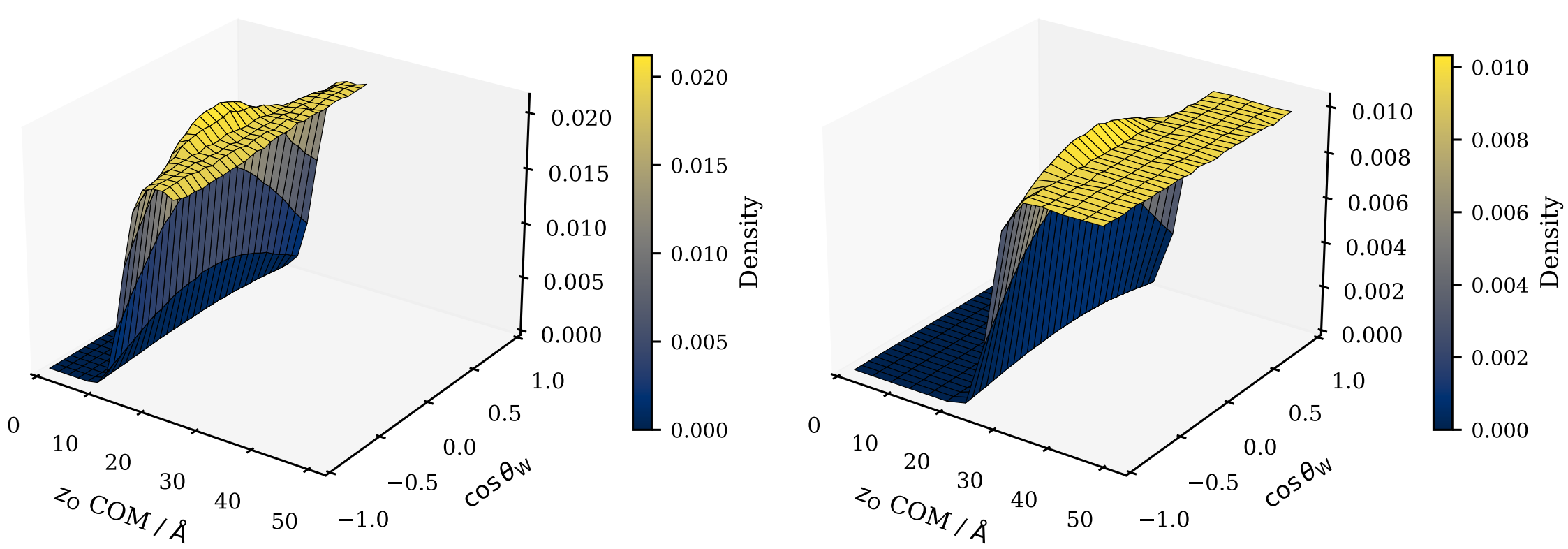

Figure S5: Three-dimensional distributions of $\cos\theta_{\mathrm{W}}$ as a function of the water oxygen position, z_{O}, for HS-I (left) and HS-II (right).

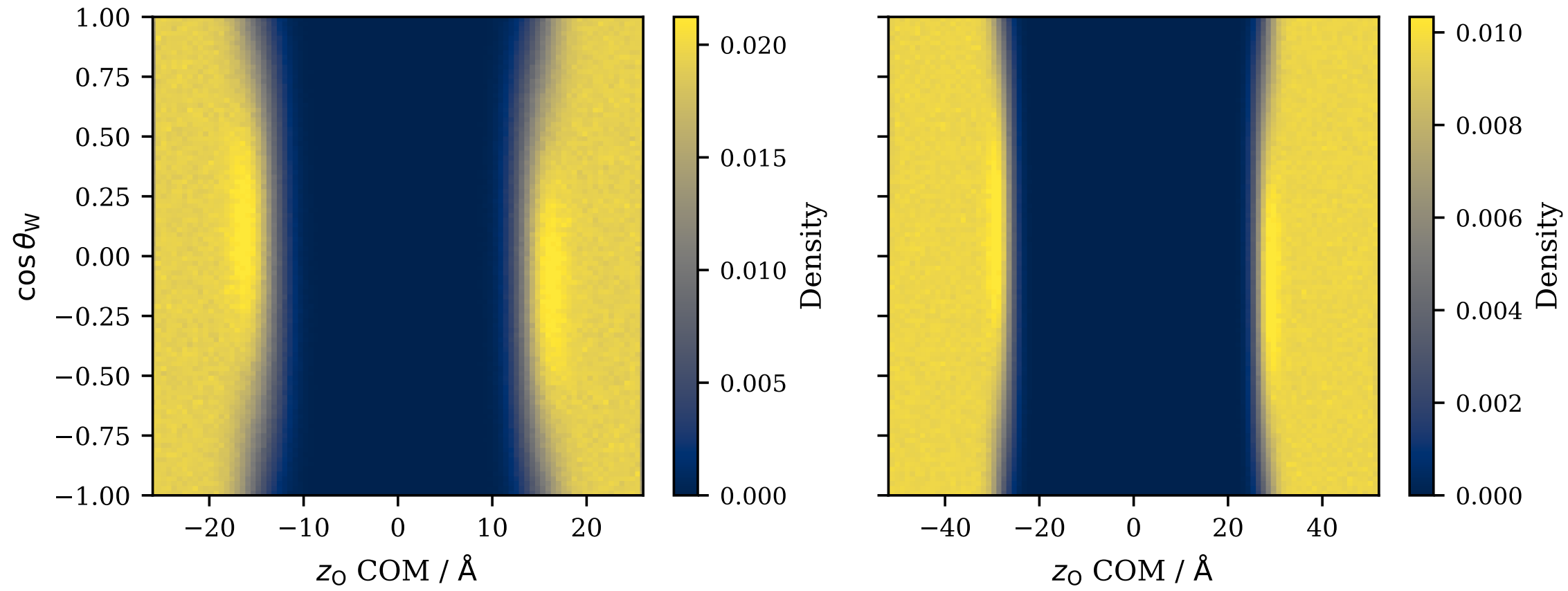

Figure S6: Two-dimensional distributions of $\cos\theta_W$ as a function of the water oxygen position, z_O, for HS-I (left) and HS-II (right).

S1.3 Bulk Reference Diffusion Coefficients

To determine bulk diffusion coefficients for O_2 in hexadecane and water, we constructed bulk reference systems with dimensions comparable to those of the bulk regions identified from the density profiles of the hexadecane/water slab systems (Figure S1). The bulk systems derived from HS-I are denoted Water-I and Hexadecane-I, whereas those derived from HS-II are denoted Water-II and Hexadecane-II. Their compositions and box dimensions are listed in Table S2.

Initial configurations for all bulk systems were generated with Packmol. Each system was minimized for 50 000 steps, followed by equilibration in the NVT ensemble for 4 ns, equilibration in the NPT ensemble for 10 ns, and a further NVT equilibration for 10 ns. Thermostat and barostat settings were identical to those used for the slab systems described in the main text.

Table S2: Compositions of the bulk reference systems used for the calculation of O_2 diffusion coefficients and the corresponding simulation box dimensions.

System	Hexadecane	Water	O_2	L_x (Å)	L_y (Å)	L_z (Å)
Water-I	0	1418	1	50.00	50.00	17.00
Hexadecane-I	99	0	1	50.00	50.00	20.00
Water-II	0	3420	1	50.00	50.00	41.00
Hexadecane-II	227	0	1	50.00	50.00	46.00

For each bulk system, 10 independent production simulations of 20 ns each were carried out in the NVT ensemble, and the center-of-mass position of O_2 was recorded every 20 fs. Each trajectory was divided into four 5 ns blocks, yielding 40 analysis segments in total. Because diffusion in these bulk systems is isotropic, we computed the three-dimensional

mean squared displacement (MSD) and estimated the diffusion coefficient from the slope of the Einstein relation, Equation S1, by linear regression over the interval from 10 ps to 1000 ps.

$$\lim_{t\to\infty} \left\langle |\vec{r}(t) - \vec{r}(0)|^2 \right\rangle = 6Dt, \tag{S1}$$

Figure S7 shows the MSD curves and corresponding linear fits for the four bulk reference systems. The resulting diffusion coefficients are listed in Table S3. The O_2 diffusion coefficient in Water-I agrees with the literature value reported by Ghysels *et al.*[1], whereas that in Hexadecane-I is lower than the corresponding literature value.

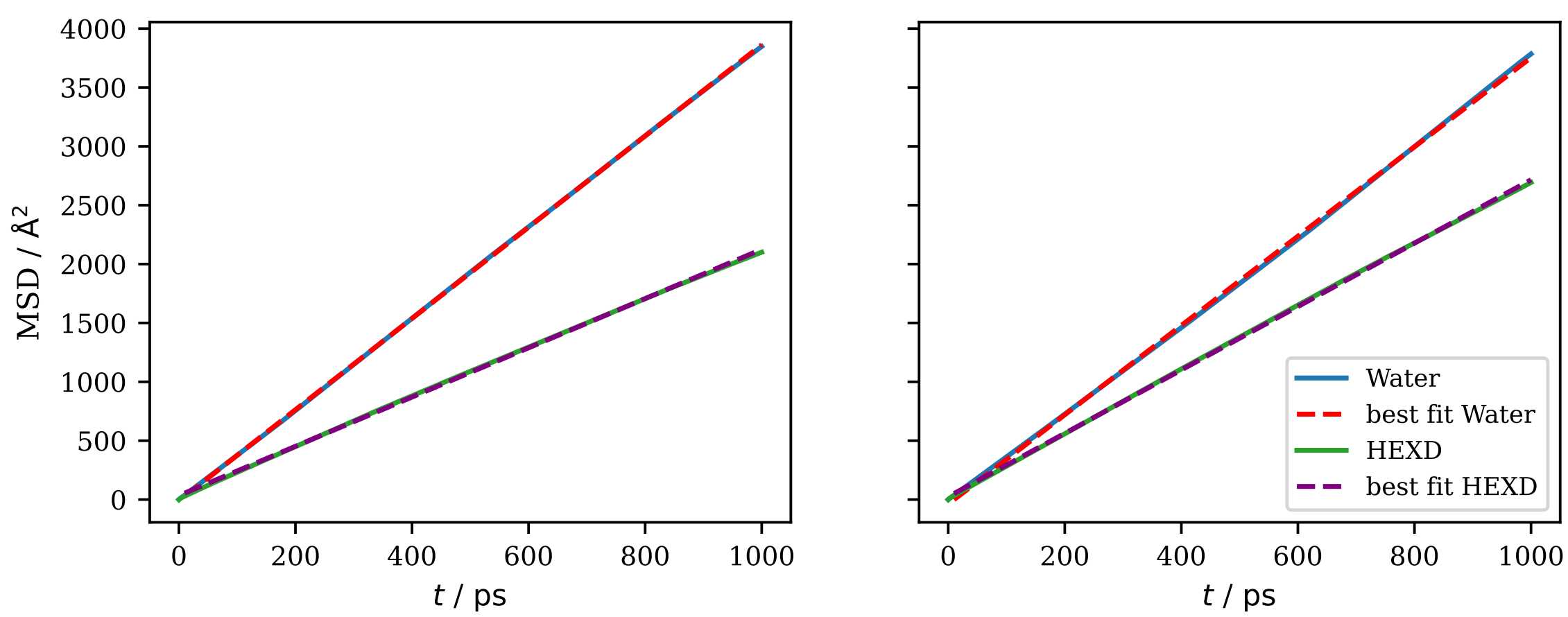

Figure S7: Three-dimensional mean squared displacements of O_2 in the four bulk reference systems considered in this study. Left panel: Water-I and Hexadecane-I; right panel: Water-II and Hexadecane-II. Diffusion coefficients obtained from linear fits to the MSDs are listed in Table S3.

Table S3: Bulk diffusion coefficients of O_2 in water and hexadecane, reported in $Å^2\,ps^{-1}$, obtained from the Einstein relation (Eq. S1). Uncertainties were estimated from the standard error of the mean and are reported as 95 % confidence intervals. Literature values are from Ghysels *et al.*[1]

System	Diffusion coefficient	Literature
Water-I	0.646 ± 0.055	0.60
Hexadecane-I	0.349 ± 0.052	0.44
Water-II	0.632 ± 0.067	—
Hexadecane-II	0.449 ± 0.039	—

S1.4 HS-I Residence-Time Analysis

We next applied the residence-time analysis to HS-I, the smaller hexadecane/water slab system corresponding to the system studied by Ghysels *et al.*[1] Figure S8 shows the convergence of the PMF obtained from the ABF simulations. The PMF becomes approximately stationary after 400 ns, and only data collected thereafter were used for the diffusivity analysis.

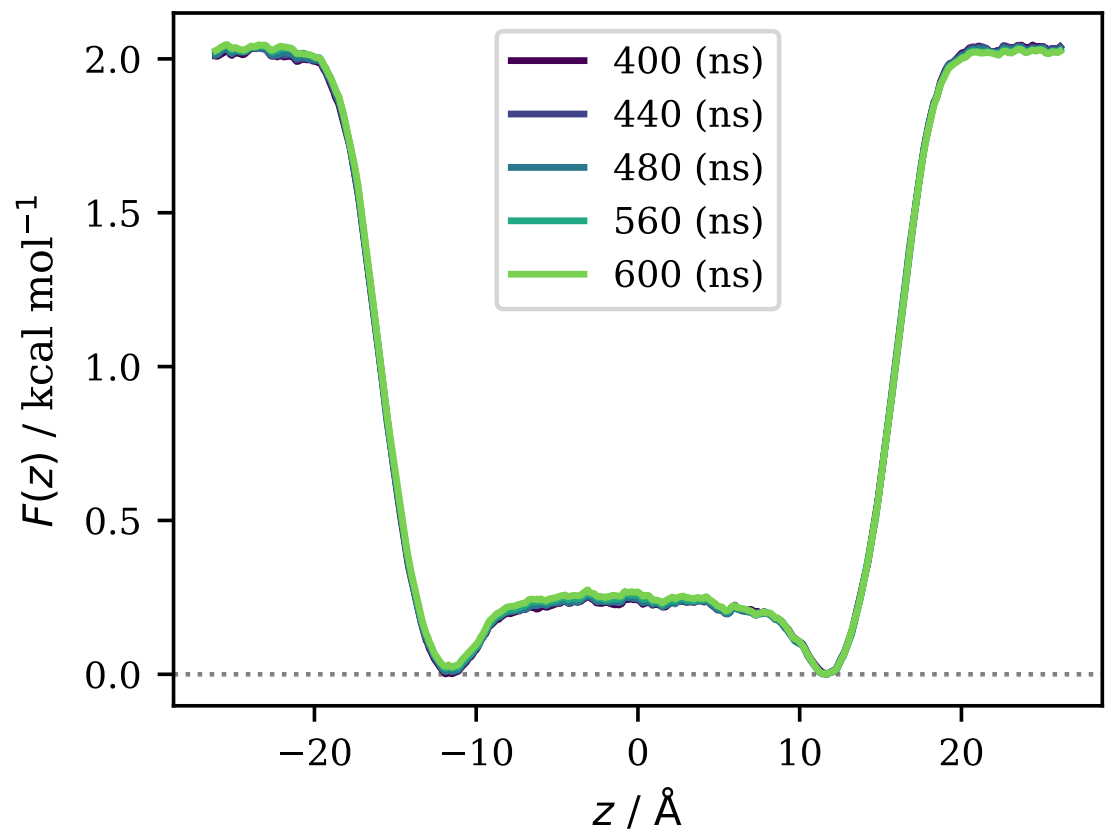

Figure S8: Convergence of the PMF from ABF simulations of O_2 permeation across the HS-I hexadecane/water slab.

The diffusivity profile obtained from the RTA (Figure S9) differs qualitatively from that reported by Ghysels *et al.*[1] In particular, the RTA-derived profile is comparatively flat and shows substantially less spatial structure.

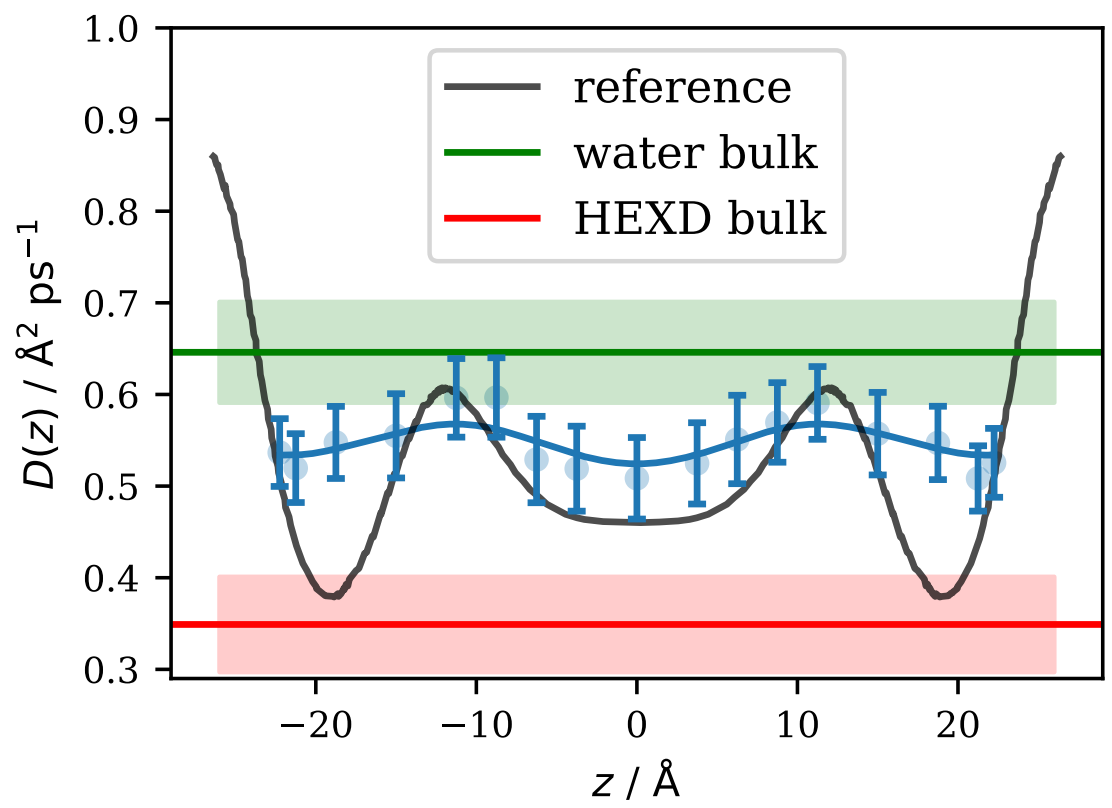

Figure S9: Diffusivity profile for O_2 permeation across the HS-I hexadecane/water slab. Symbols denote the computed diffusivities; solid lines show spline interpolations included for visual guidance only. Error bars represent uncertainties estimated from blocking analysis of 10 independent O_2 trajectories. The literature profile was digitized from Ghysels *et al.*[1]

We note that the RTA-derived profile reproduces the bulk-water diffusivity more closely than the bulk-hexadecane diffusivity. At the same time, our orientational analysis indicates that fully bulk-like behavior should not necessarily be expected in the interior of the HS-I hexadecane slab. We therefore consider two possible explanations for the qualitative differences between the present profile and that reported by Ghysels *et al.*[1] First, Ghysels *et al.* represented the diffusivity profile using a truncated Fourier cosine expansion. While this basis was introduced to smooth the profile and avoid overfitting, the inferred profile may nevertheless retain basis-dependent structure. Second, the RTA assumes that the diffusivity is approximately constant within each spatial interval. In a small system such as HS-I, where bulk-like regions are limited, this approximation may smooth or attenuate genuine spatial variation in the diffusivity profile.

At present, we do not attempt to distinguish definitively between these possibilities. However, the main conclusions of this work for the membrane systems are unaffected, because their consistency has been corroborated by propagator-level analysis in the main text.

S1.5 Additional HS-I Profiles

Additional PMF and diffusivity data for HS-I are shown in Figures S10–S12. These plots show the individual profiles obtained for each O_2 molecule and complement the averaged diffusivity profile shown in Figure S9.

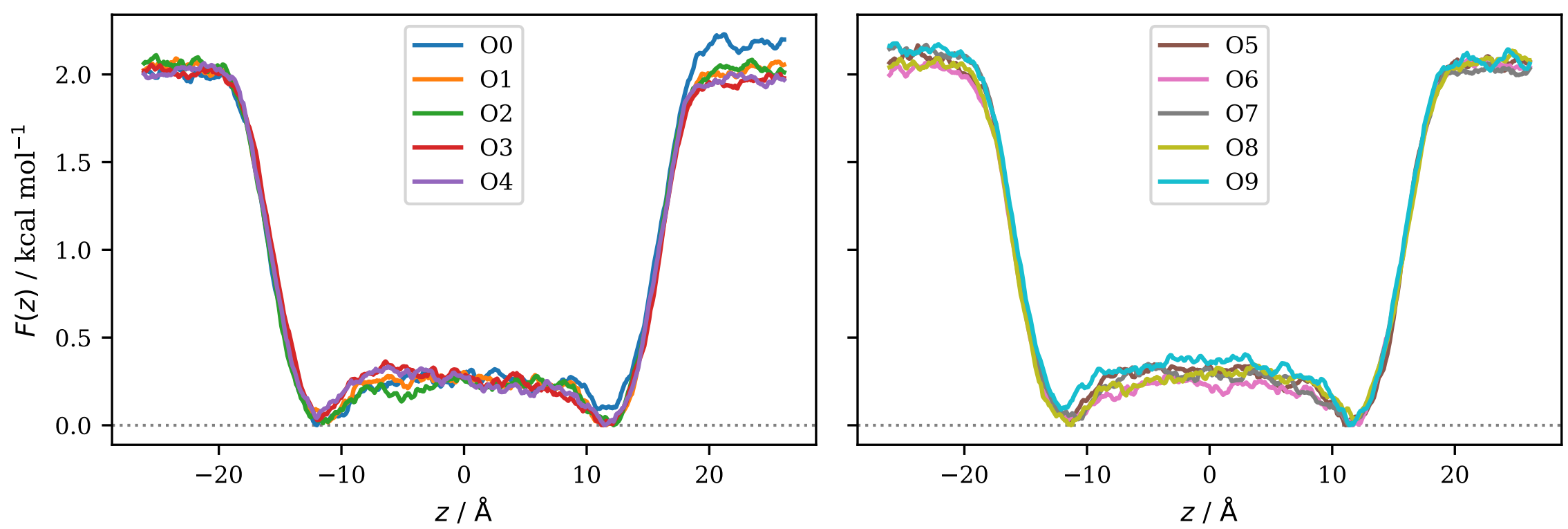

Figure S10: Individual raw PMF profiles for O_2 permeation across the HS-I hexadecane/water slab after 600 ns of ABF simulation.

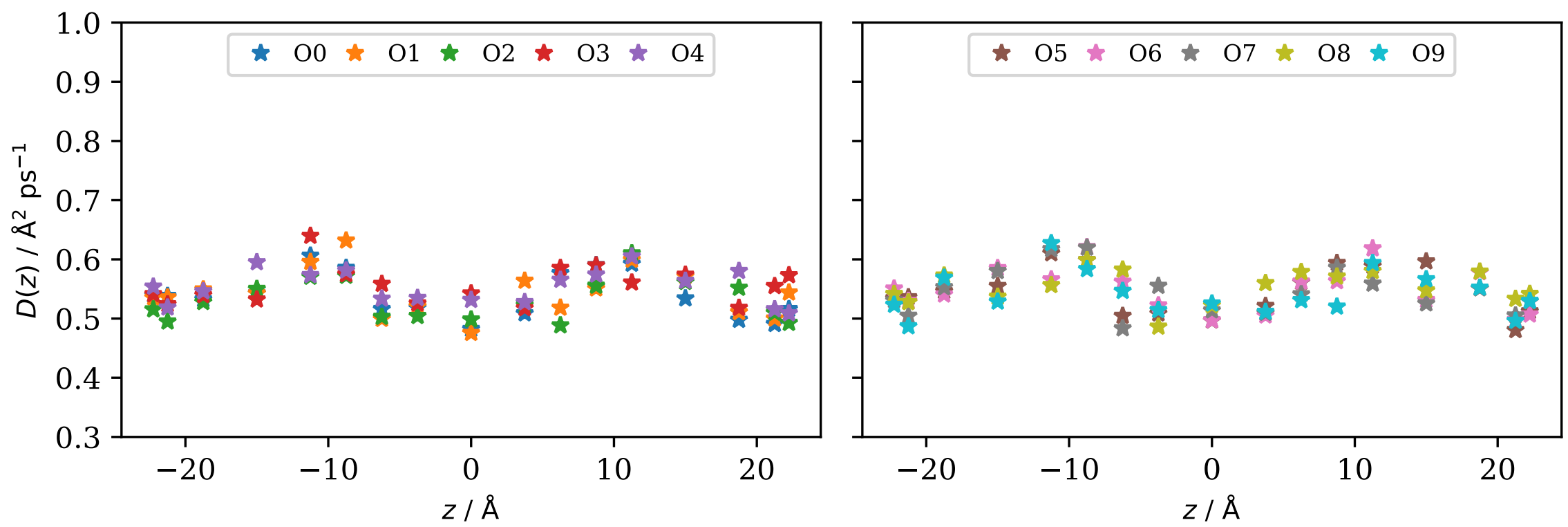

Figure S11: Individual raw diffusivity profiles for O_2 permeation across the HS-I hexadecane/water slab.

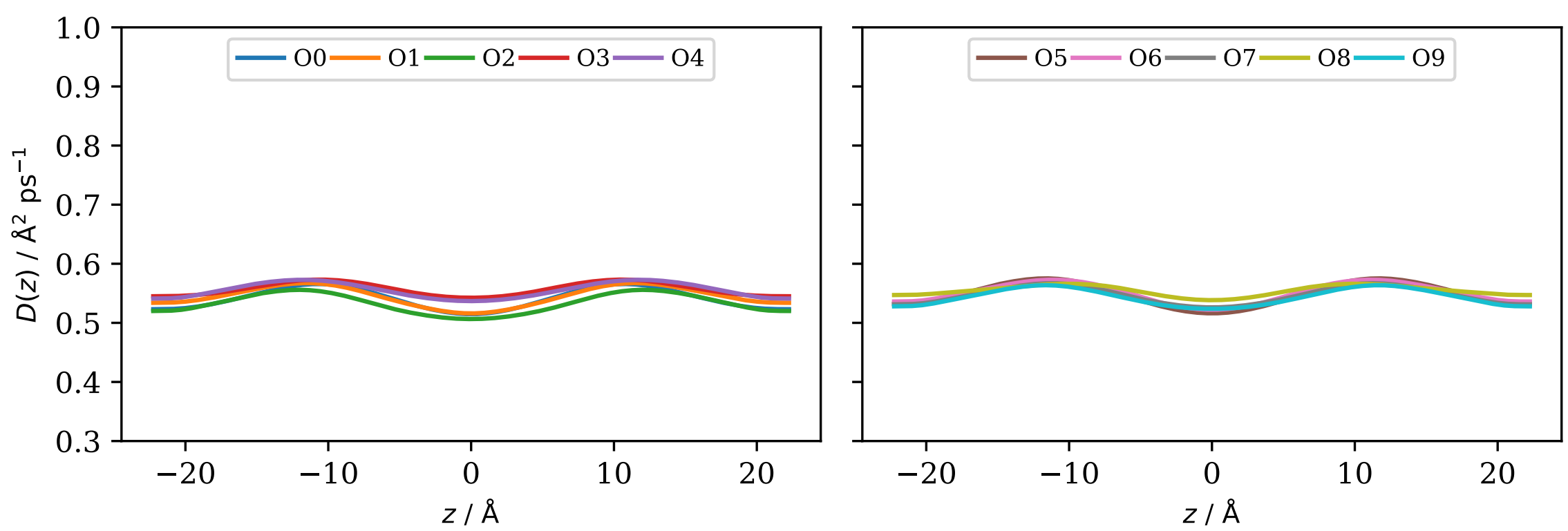

Figure S12: Individual spline-smoothed diffusivity profiles for O_2 permeation across the HS-I hexadecane/water slab.

S1.6 Additional HS-II Profiles

Additional PMF and diffusivity data for HS-II are shown in Figures S13–S15. These plots show the individual profiles obtained for each O_2 molecule and thus complement the averaged results presented in the main text.

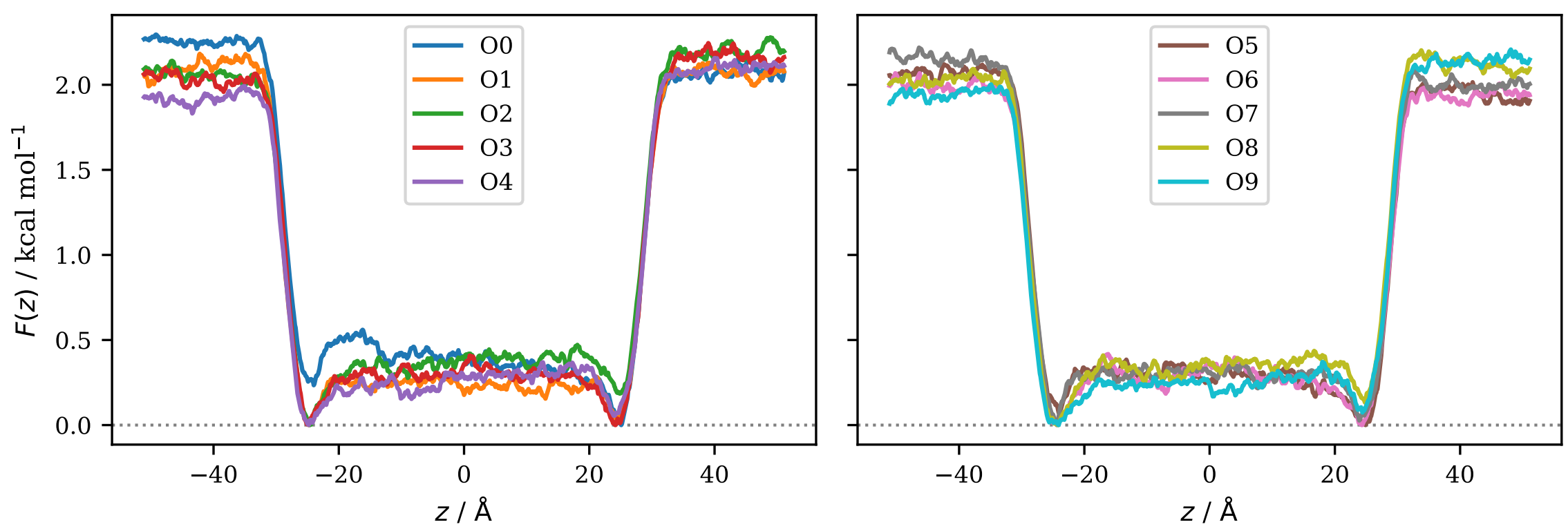

Figure S13: Individual PMFs for O_2 permeation across the HS-II hexadecane/water slab. The corresponding average profile is shown in the main text.

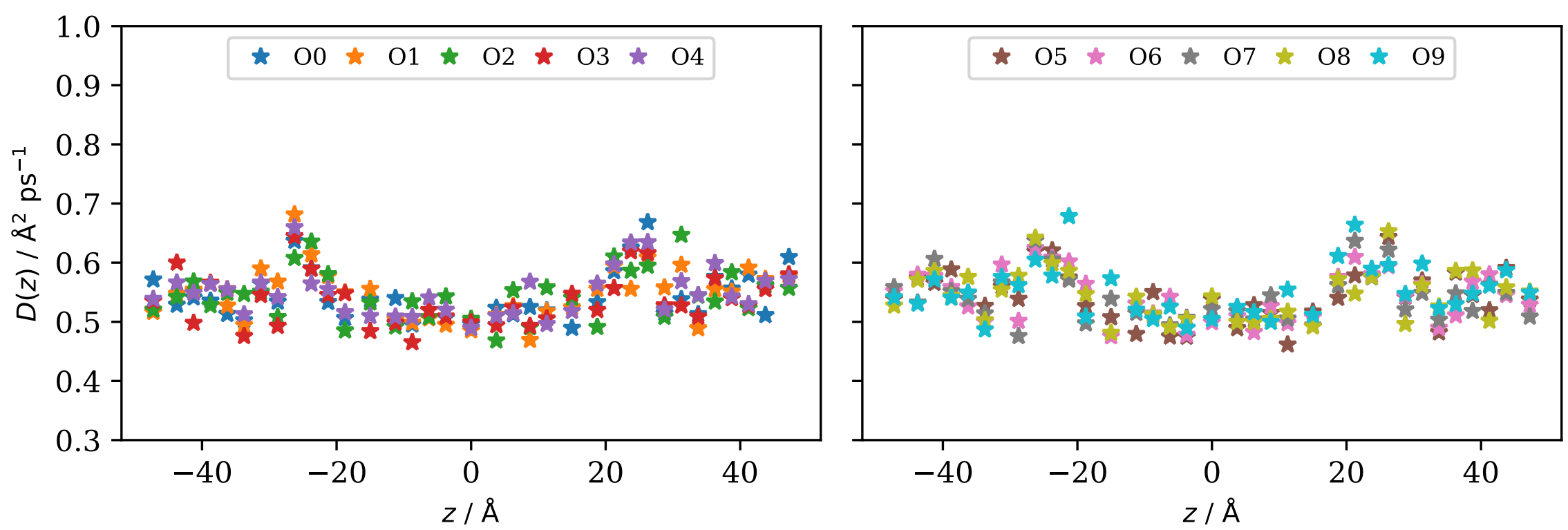

Figure S14: Individual raw diffusivity profiles for O_2 permeation across the HS-II hexadecane/water slab.

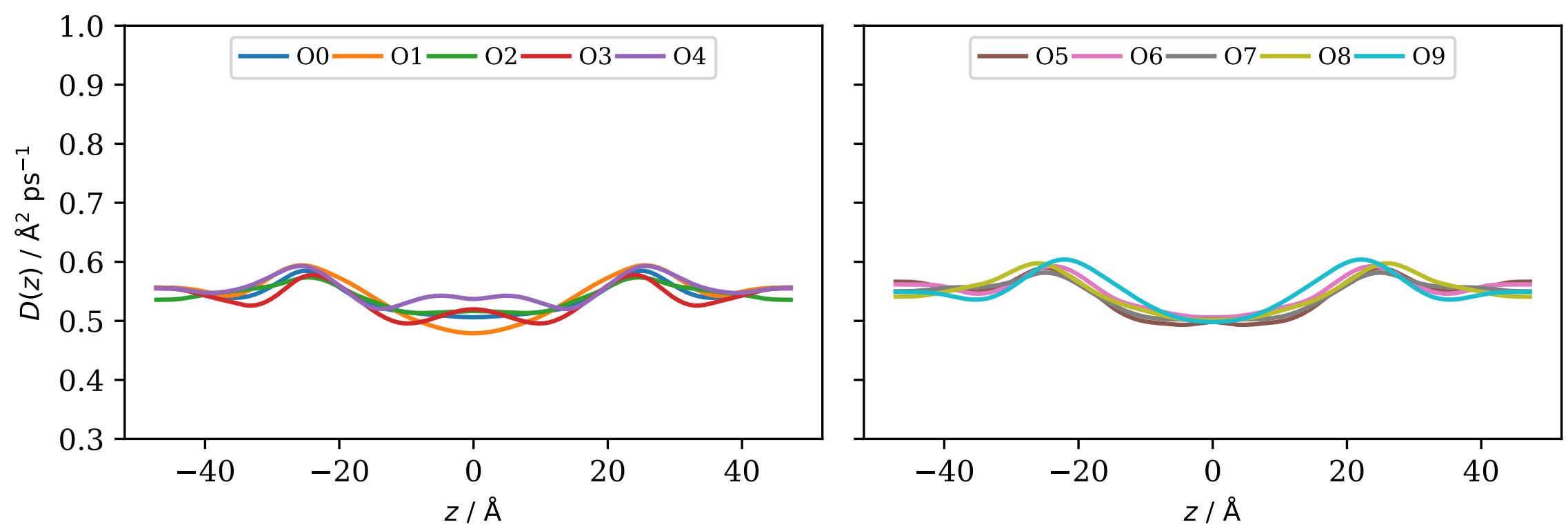

Figure S15: Individual spline-smoothed diffusivity profiles for O_2 permeation across the HS-II hexadecane/water slab. The corresponding average profile is shown in the main text.

S2 Water Permeation through the POPC Bilayer

S2.1 PMF Evolution before Bias Convergence

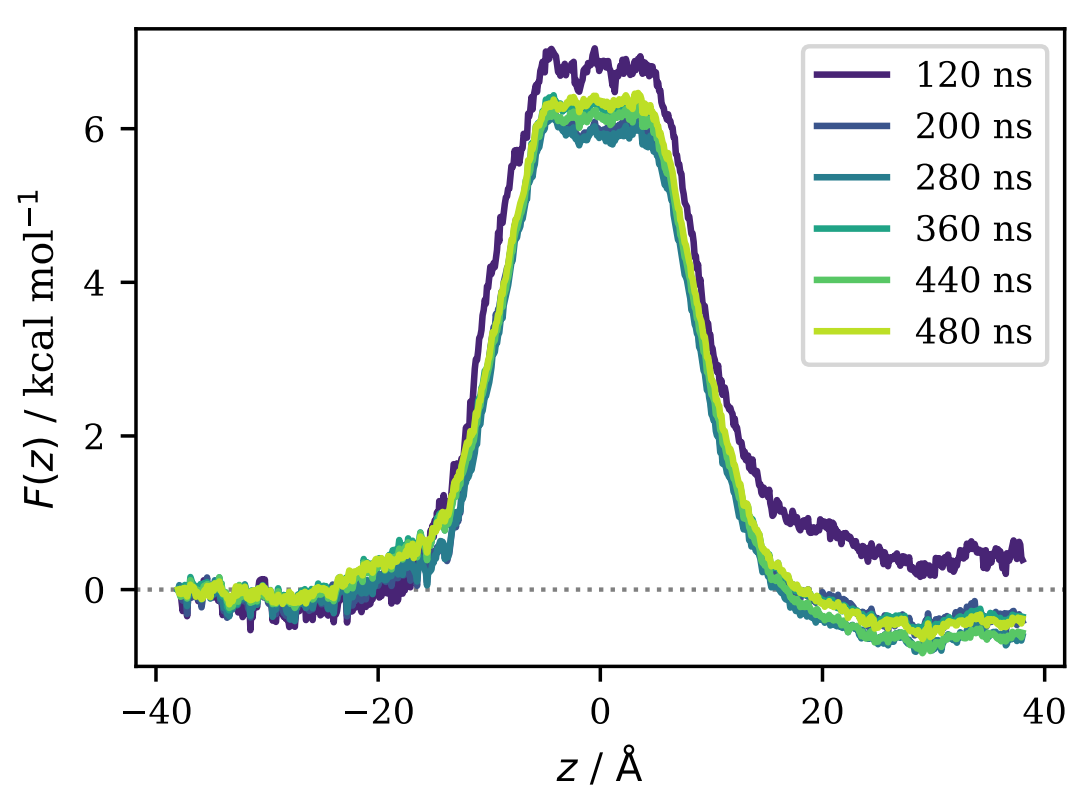

Figure S16: Evolution of the PMF during the initial stage of the ABF simulation, showing asymmetry between the two membrane leaflets. Because the ABF bias is not yet converged during this time period, these trajectory data were not used for the RTA analysis.

S2.2 Comparison of ABF and Umbrella-Sampling PMFs

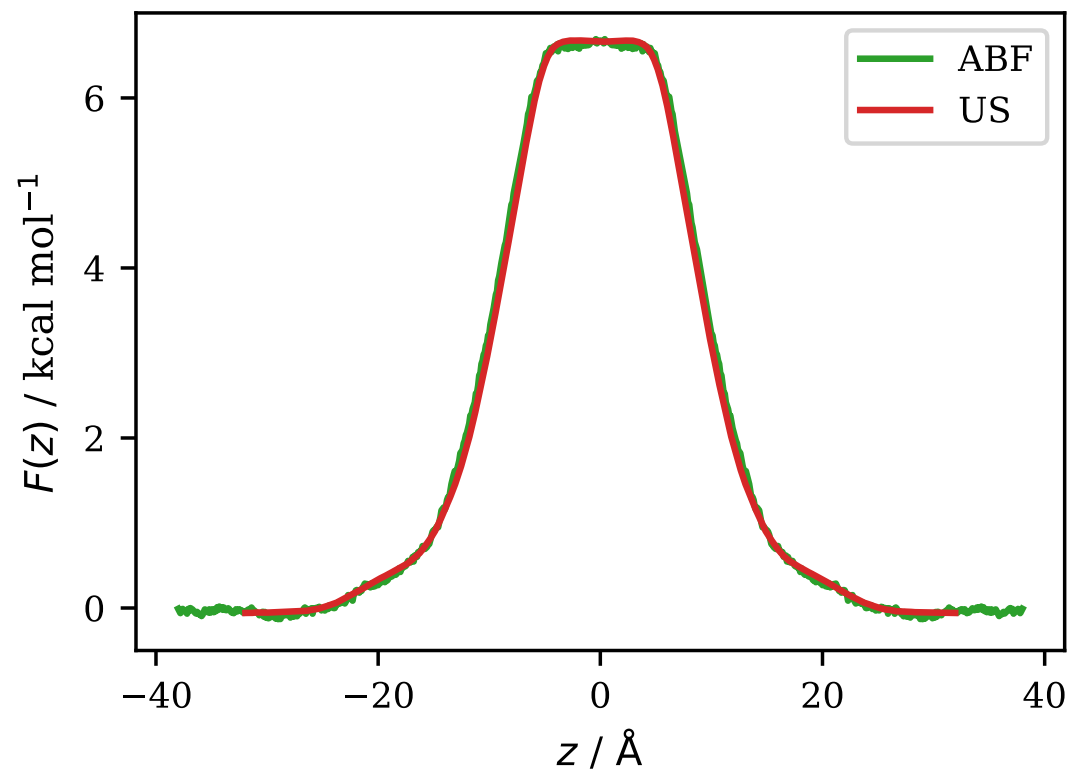

Figure S17: PMFs of water permeation through the POPC lipid bilayer obtained from ABF simulations in this work and from umbrella sampling in our previous study.[2] The two PMF estimates are in close agreement. Both systems contain the same number of lipids, but the present POPC/water system has a bulk-water region that is 12 Å thicker than in the previous study.[2]

S2.3 Extended Propagator Comparisons

To assess sensitivity to the PMF estimate, each RTA-, PACF-, and VACF-derived diffusivity profile was combined separately with the ABF- and US-derived PMF estimates. Figure S18 shows the resulting model predictions over the complete range of lag times. The relative performance of the three diffusivity profiles is unchanged between the two PMF estimates: the VACF model provides the closest agreement at very short lag times, the RTA model performs best over an intermediate range, and the PACF model gives somewhat closer agreement at the longest lag times. Thus, the relative ranking of the diffusivity profiles is unchanged by the choice of PMF estimate.

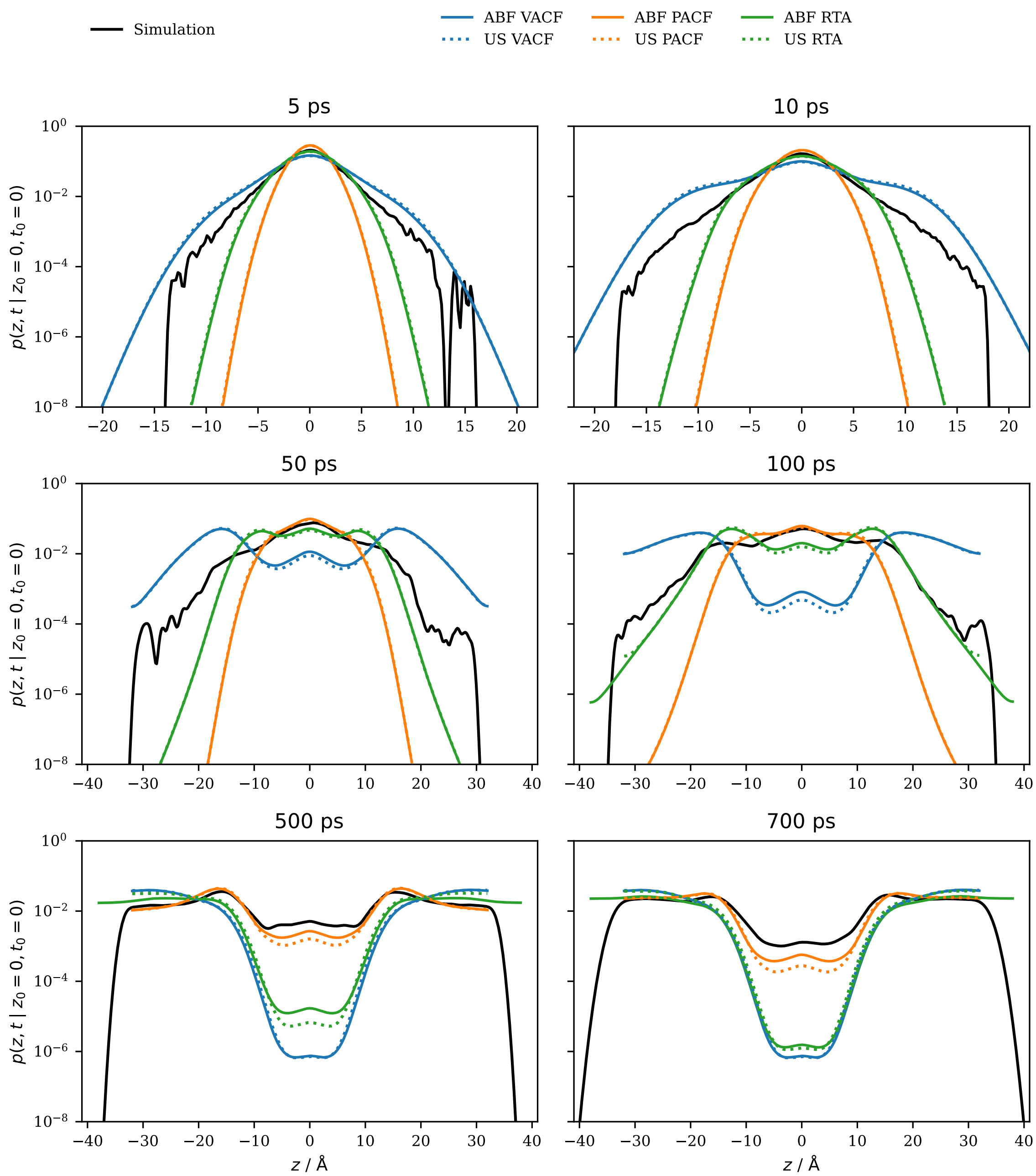

Figure S18: POPC/water propagators, $p(z, t \mid z_0, t_0)$, at lag times ranging from $t = 5\,\mathrm{ps}$ to $t = 700\,\mathrm{ps}$, obtained from simulations and diffusion models using the RTA-, PACF-, and VACF-derived diffusivity profiles. For each diffusivity profile, predictions obtained using the common ABF-derived estimate and the common US-derived estimate are shown.

S3 Solute Permeation through the Stratum Corneum Membrane

S3.1 ABF Window Definitions and Sampled Time Intervals

As shown in our previous study, obtaining well-converged PMFs for the SC system is nontrivial.[2] Accordingly, the CV was divided into 7 sequentially overlapping windows with a spacing of 5 Å, as summarized in Table S4. The initial configurations for each window were extracted from trajectory frames of a preliminary single-window ABF simulation in which the solute occupied the corresponding target position along the CV. The solutes were confined within each window using flat-bottomed harmonic walls with a force constant of 5 kcal mol^{-1} Å^{-2}. The resulting PMFs from the individual windows were aligned in their overlap regions using additive constants and then combined into a single continuous PMF.

Table S4: Window definitions based on the collective-variable spacing and the corresponding data included for the residence-time analysis.

Window	z (Å)	Water (ns)	Acetone (ns)	6-MHO (ns)
1	-45 to -30	320	280	160
2	-35 to -20	320	280	160
3	-25 to -5	320	280	280
4	-10 to 10	280	320	160
5	5 to 25	320	280	280
6	20 to 35	320	280	160
7	30 to 45	320	280	160

S3.2 Window-Wise PMF Convergence

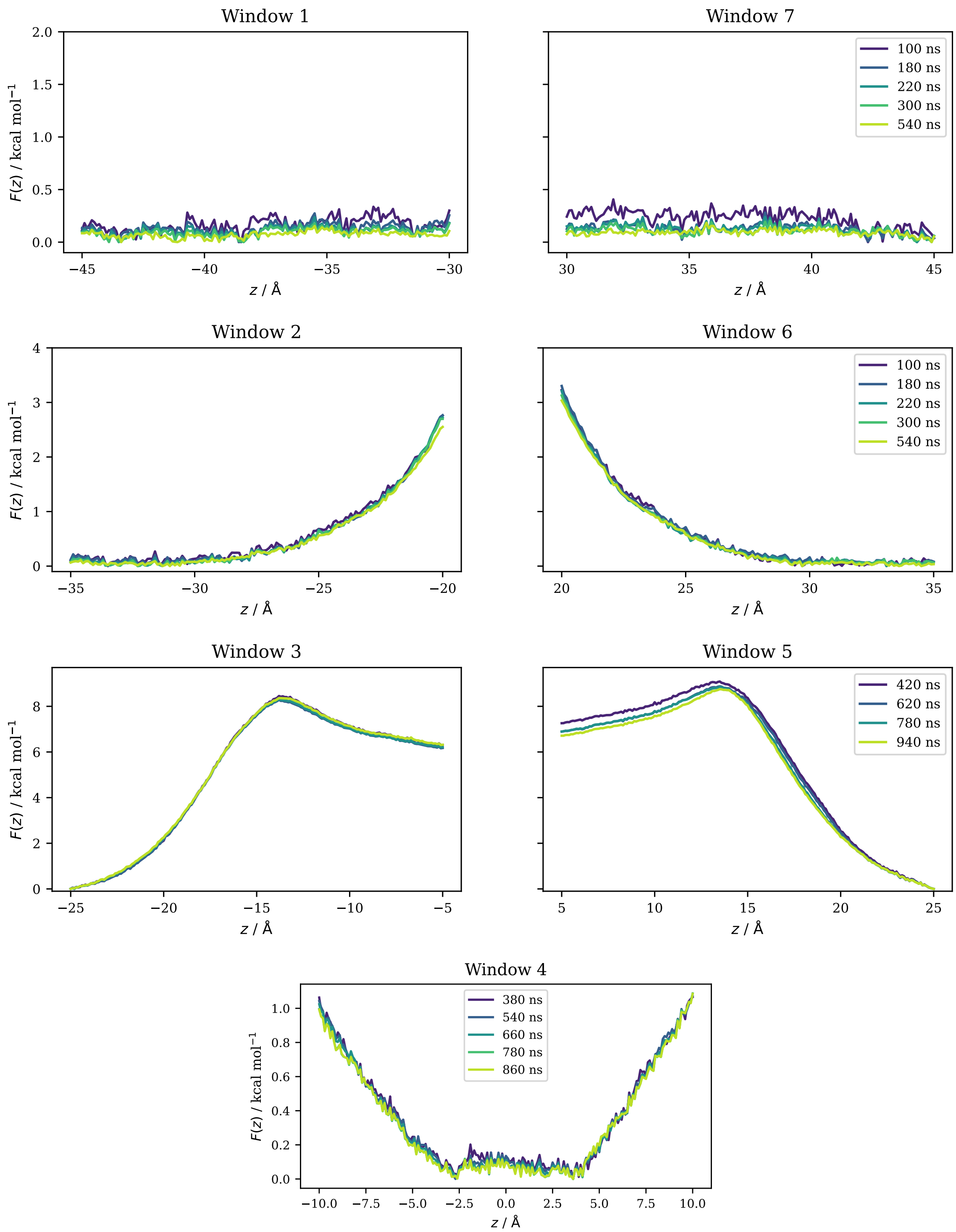

Figure S19: Window-wise convergence of the PMFs for water permeation through the SC membrane.

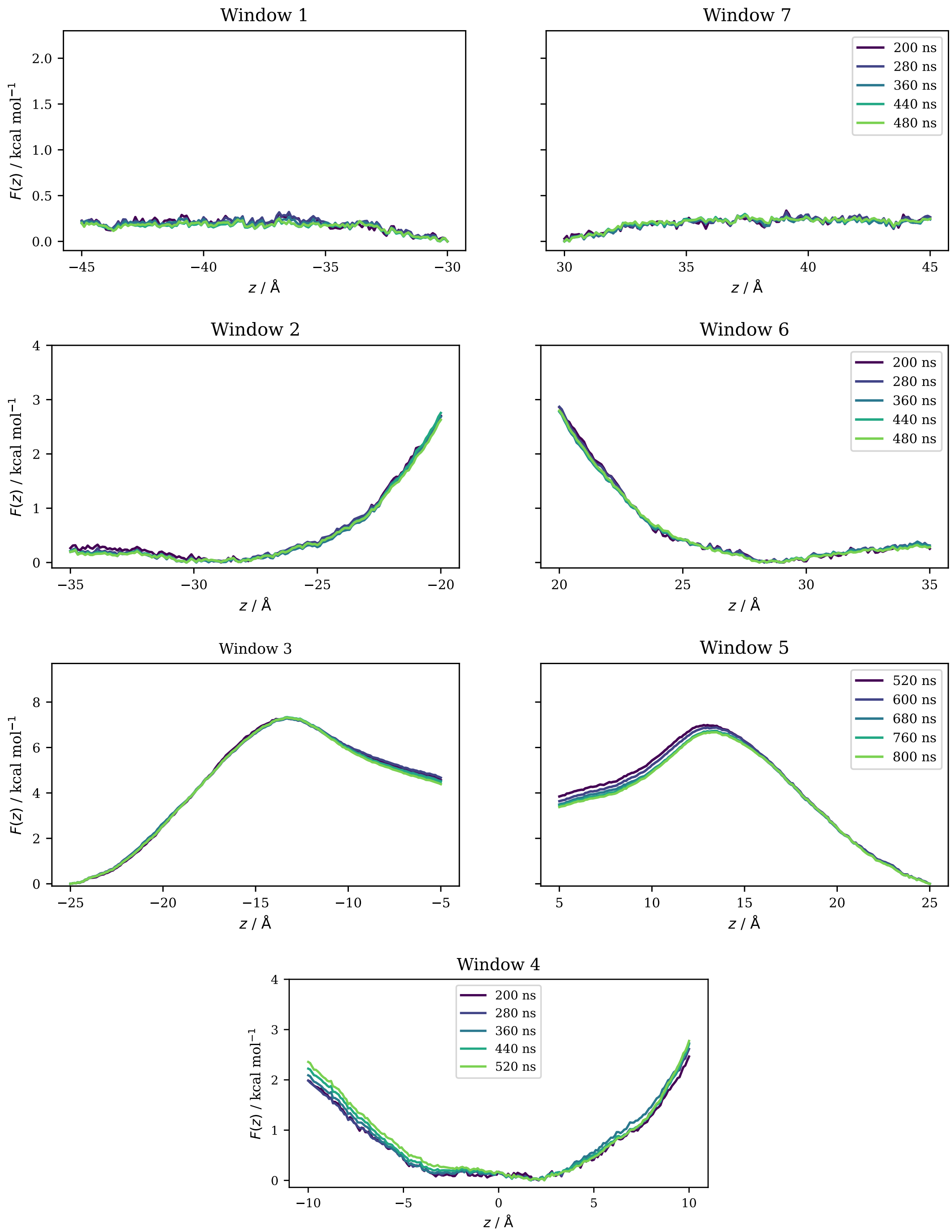

Figure S20: Window-wise convergence of the PMFs for acetone permeation through the SC membrane obtained from ABF simulations. The RTA analysis was carried out using the simulation data specified in the legends.

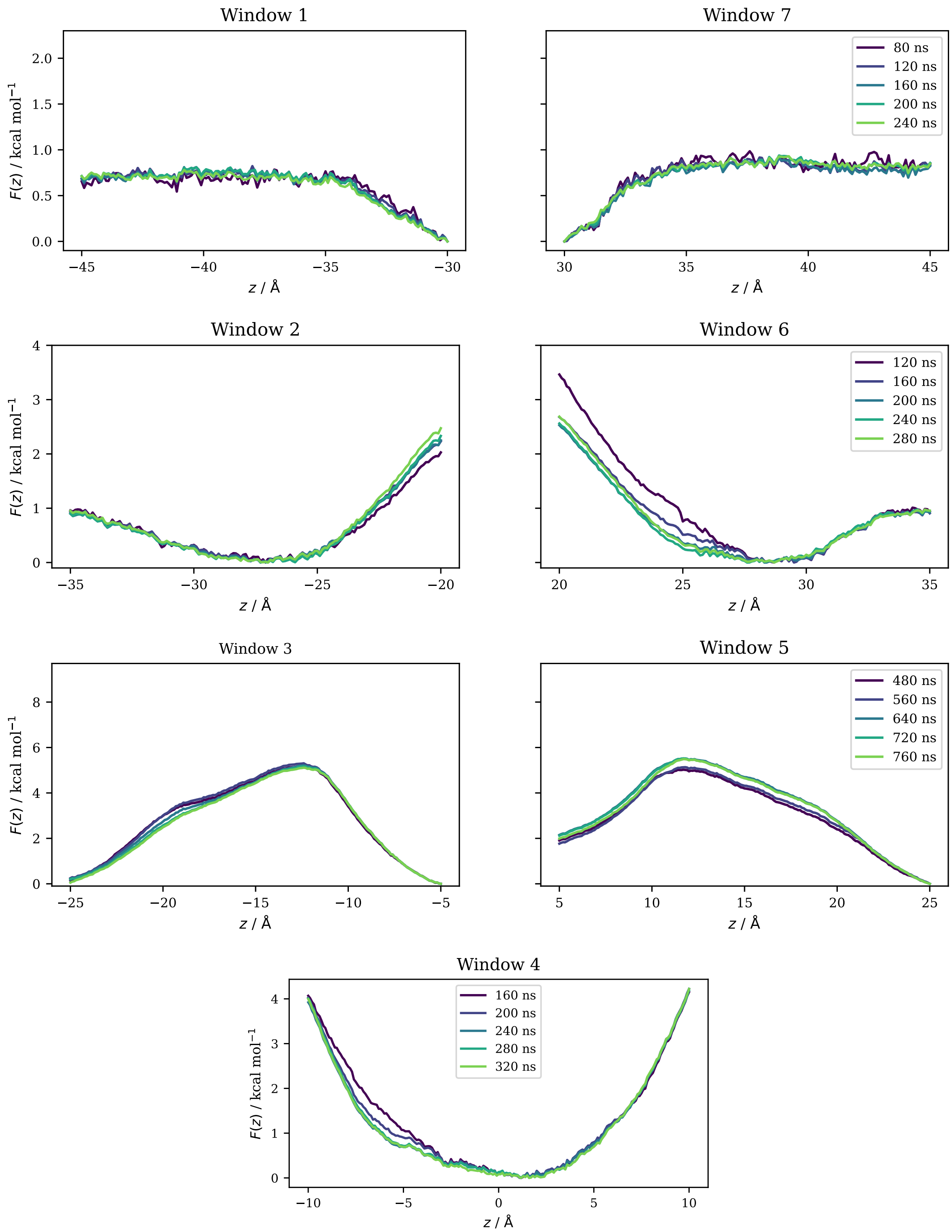

Figure S21: Window-wise convergence of the PMFs for 6-MHO permeation through the SC membrane obtained from ABF simulations. The RTA analysis was carried out using the simulation data specified in the legends.

S3.3 Comparison of PMF Estimates from Different Sampling Methods

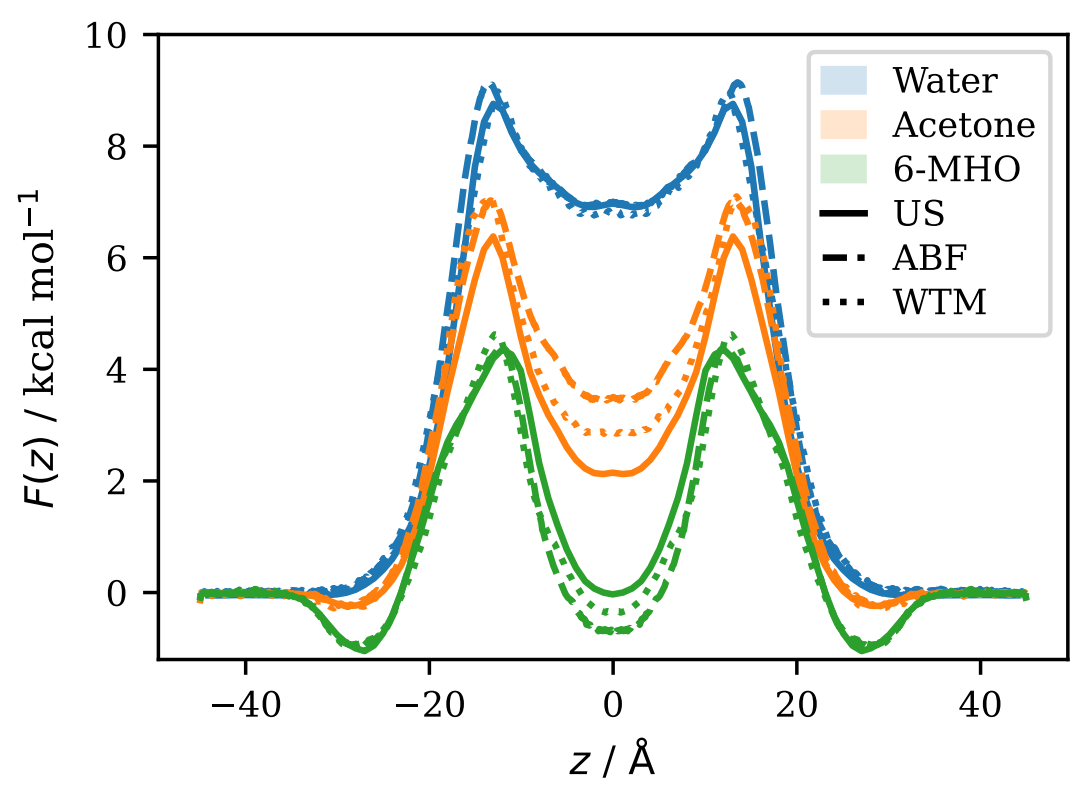

Figure S22: PMFs of solute permeation through the SC membrane obtained from ABF simulations in this work and from umbrella sampling (US) and well-tempered metadynamics (WTM) simulations in our previous study.[2] The PMFs are consistent for water. For acetone and 6-MHO, differences of less than 1 kcal mol^{-1} are observed, primarily near the membrane center.

S3.4 Extended Propagator Comparisons

As for POPC, each SC diffusivity profile was combined separately with the ABF- and US-derived PMF estimates. The resulting propagators are compared with the unbiased MD reference distributions in Figures S23–S25. Substituting the US-derived PMF estimate for the ABF-derived estimate does not change the relative performance of the diffusivity profiles: the RTA model gives the closest agreement for water, the RTA and PACF models perform comparably for acetone and 6-MHO, and the VACF models predict systematically broader spreading.

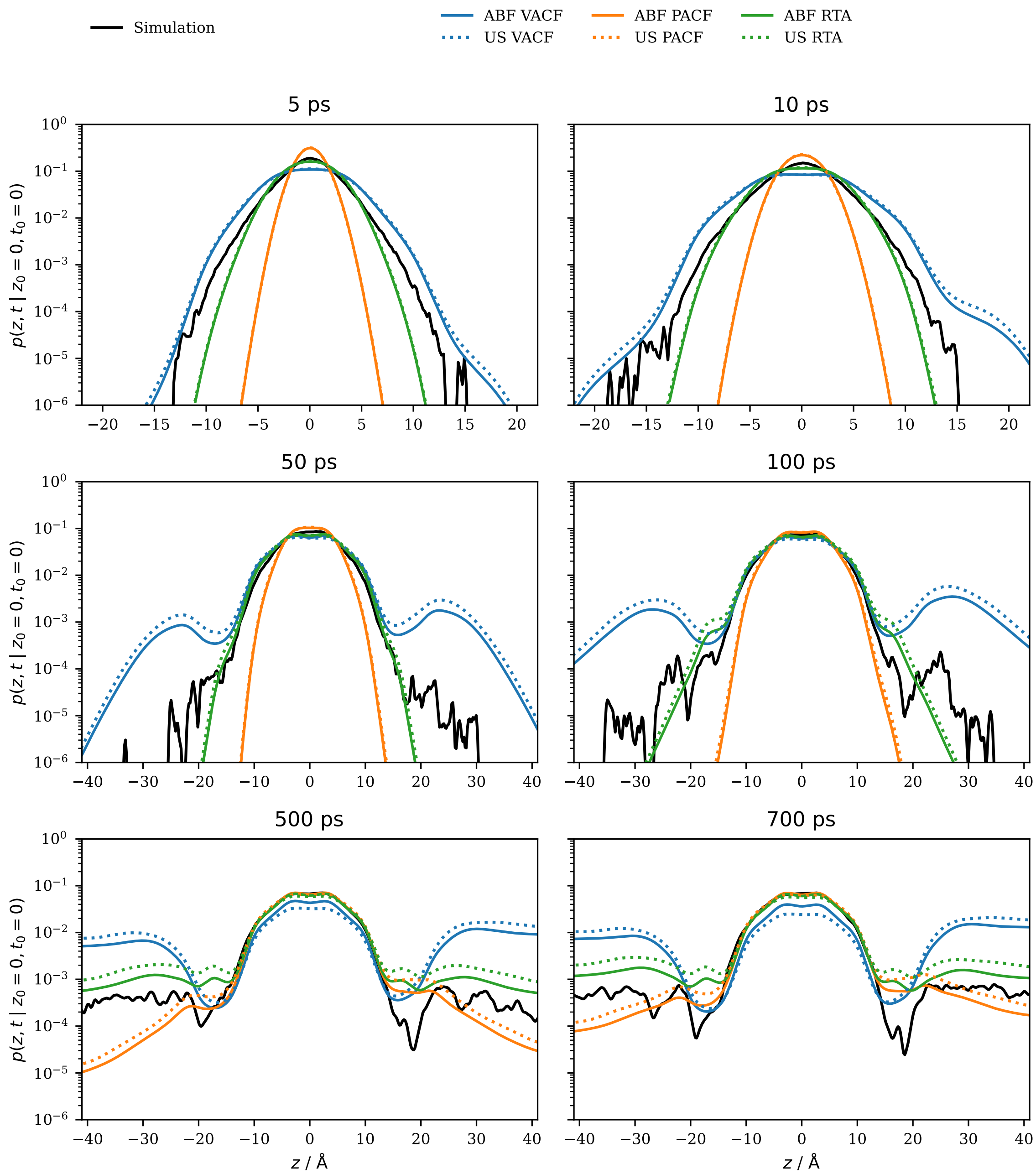

Figure S23: SC/water propagators, $p(z, t \mid z_0, t_0)$, at lag times ranging from $t = 5\,\text{ps}$ to $t = 700\,\text{ps}$, obtained from simulations and diffusion models using the RTA-, PACF-, and VACF-derived diffusivity profiles. For each diffusivity profile, predictions obtained using the common ABF-derived estimate and the common US-derived estimate are shown.

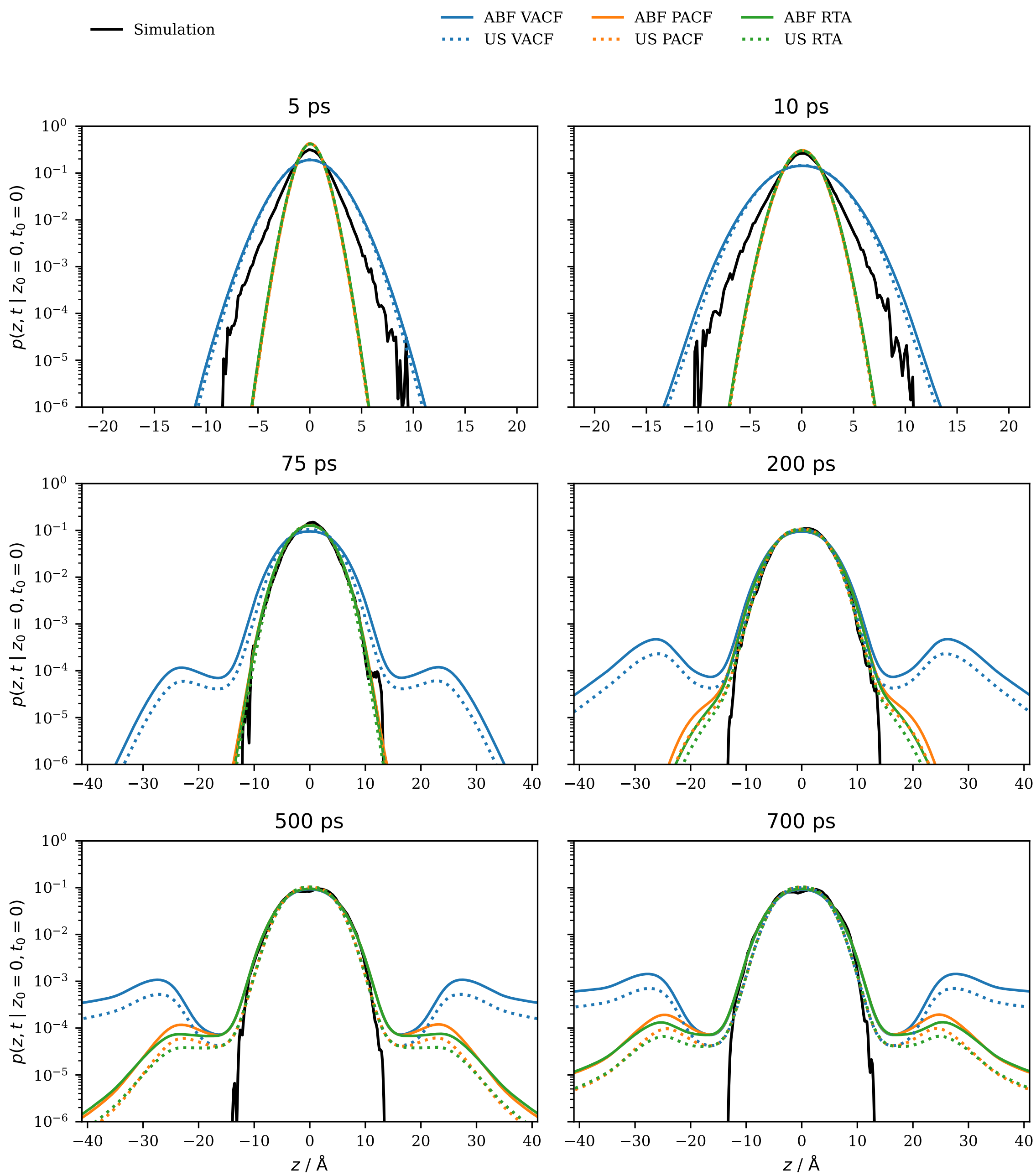

Figure S24: SC/acetone propagators, $p(z, t \mid z_0, t_0)$, at lag times ranging from $t = 5\,\mathrm{ps}$ to $t = 700\,\mathrm{ps}$, obtained from simulations and diffusion models using the RTA-, PACF-, and VACF-derived diffusivity profiles. For each diffusivity profile, predictions obtained using the common ABF-derived estimate and the common US-derived estimate are shown.

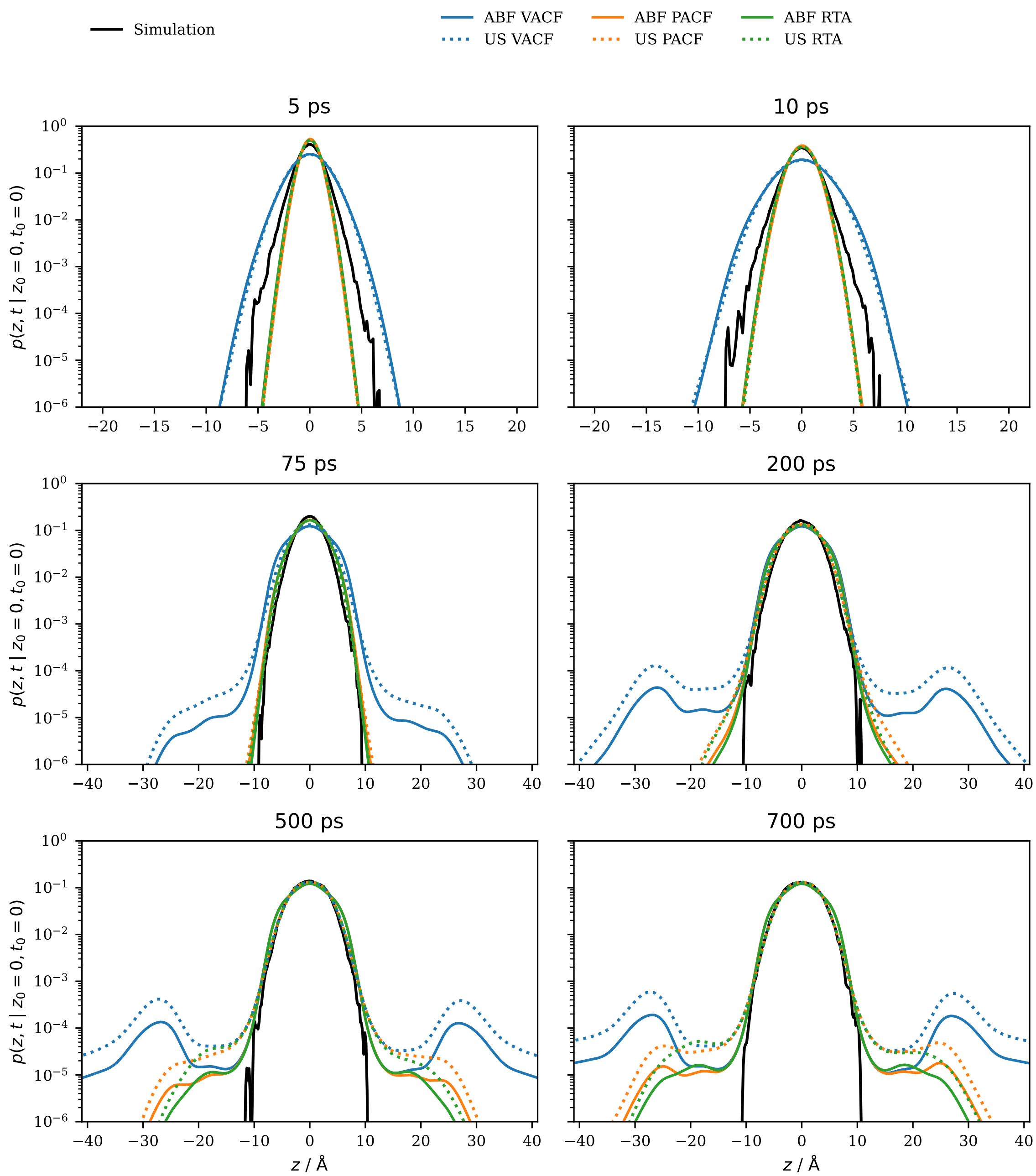

Figure S25: SC/6-MHO propagators, $p(z, t \mid z_0, t_0)$, at lag times ranging from $t = 5\,\mathrm{ps}$ to $t = 700\,\mathrm{ps}$, obtained from simulations and diffusion models using the RTA-, PACF-, and VACF-derived diffusivity profiles. For each diffusivity profile, predictions obtained using the common ABF-derived estimate and the common US-derived estimate are shown.

S4 Permeability Analysis

S4.1 Definition of the Permeability Integration Bounds

To estimate permeabilities for the POPC bilayer and SC membrane within the inhomogeneous solubility–diffusion (ISD) framework, we defined the membrane half-thickness, $h/2$, from the water density profile as the position beyond which the water density becomes negligibly small. Using this criterion, we chose $h/2 = 11.0$ Å for POPC and $h/2 = 17.0$ Å for SC. For POPC, this definition was applied analogously to the procedure used previously for SC.[3] Figure S26 shows the water density profile for the POPC system together with the resulting integration cutoff.

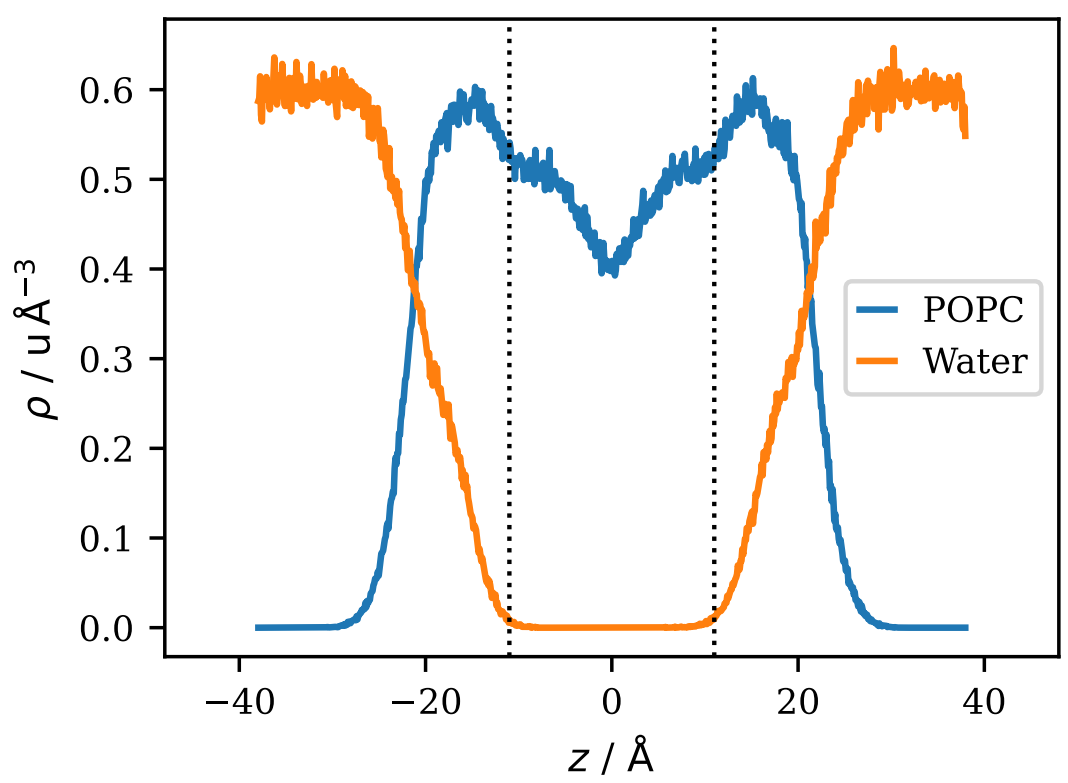

Figure S26: Water density profile of the POPC bilayer. The dotted vertical line indicates the cutoff used to define the integration bound for the permeability calculation.

S4.2 Supplementary Permeability Coefficients

Table S5 reports permeabilities obtained from the full factorial combination of the two PMF estimates and three diffusivity profiles. Comparisons across a row hold the PMF fixed and therefore show the influence of the diffusivity profile. Comparisons between the ABF and US rows for a fixed diffusivity quantify sensitivity to the PMF estimate. The ordering associated with the three diffusivity profiles is unchanged between the two estimates for every system, although the absolute permeability coefficients, particularly for the SC systems, exhibit appreciable PMF sensitivity.

Table S5: Membrane permeabilities, reported in $\mathrm{cm\,s^{-1}}$, obtained using the inhomogeneous solubility–diffusion model. Each RTA-, PACF-, and VACF-derived diffusivity profile was combined separately with the ABF- and US-derived PMF estimates.

System	PMF	RTA	PACF	VACF	Literature
POPC/water[a]	ABF	6.03×10^{-3}	2.51×10^{-3}	1.39×10^{-2}	1.40×10^{-3}
POPC/water	US	6.20×10^{-3}	2.34×10^{-3}	1.30×10^{-2}	—
SC/water[b]	ABF	3.81×10^{-5}	3.28×10^{-5}	2.53×10^{-4}	1.88×10^{-5}
SC/water	US	8.19×10^{-5}	5.65×10^{-5}	4.15×10^{-4}	—
SC/acetone	ABF	6.73×10^{-4}	8.00×10^{-4}	5.37×10^{-3}	—
SC/acetone	US	2.21×10^{-3}	2.66×10^{-3}	1.79×10^{-2}	—
SC/6-MHO	ABF	3.94×10^{-2}	5.02×10^{-2}	3.11×10^{-1}	—
SC/6-MHO	US	3.60×10^{-2}	4.55×10^{-2}	2.82×10^{-1}	—

[a] Literature value from Venable *et al.*[4] Their simulations were performed at 303.15 K.
[b] Literature value from Reuter *et al.*[5] For a broader comparison across force fields and methods, see Thomas *et al.*[3]

S5 Methodological Validation of the Residence-Time Approach

S5.1 Local Dynamical Statistics

To further examine the assumptions underlying the residence-time analysis (RTA), we consider local dynamical statistics computed from trajectory segments confined to spatial intervals of width L. Specifically, we compare the local mean-square displacement (MSD) and the survival probability $S(t)$ for representative bulk and membrane-center regions in the SC/water system.

S5.1.1 Local Mean-Square Displacements

The local MSD was computed from contiguous trajectory segments that remain within the interval throughout the observation time. Because the dynamics are confined to a finite domain, the MSD cannot grow indefinitely and necessarily deviates from linear behavior on timescales comparable to the mean residence time τ_r. Consequently, deviations from linearity on these timescales cannot, by themselves, be interpreted as evidence against an effective Smoluchowski description.

The resulting MSDs are shown in Figure S27. Both the bulk and membrane-center regions exhibit an approximately linear regime at intermediate times, followed by the expected saturation as the particle explores the entire interval. The membrane-center region displays more pronounced deviations from linearity than the bulk. However, because the MSD is strongly influenced by the finite extent of the observation interval, it provides only an indirect test of the assumptions underlying the RTA and does not

determine whether the corresponding first-passage process is adequately described by an effective Markovian diffusion model.

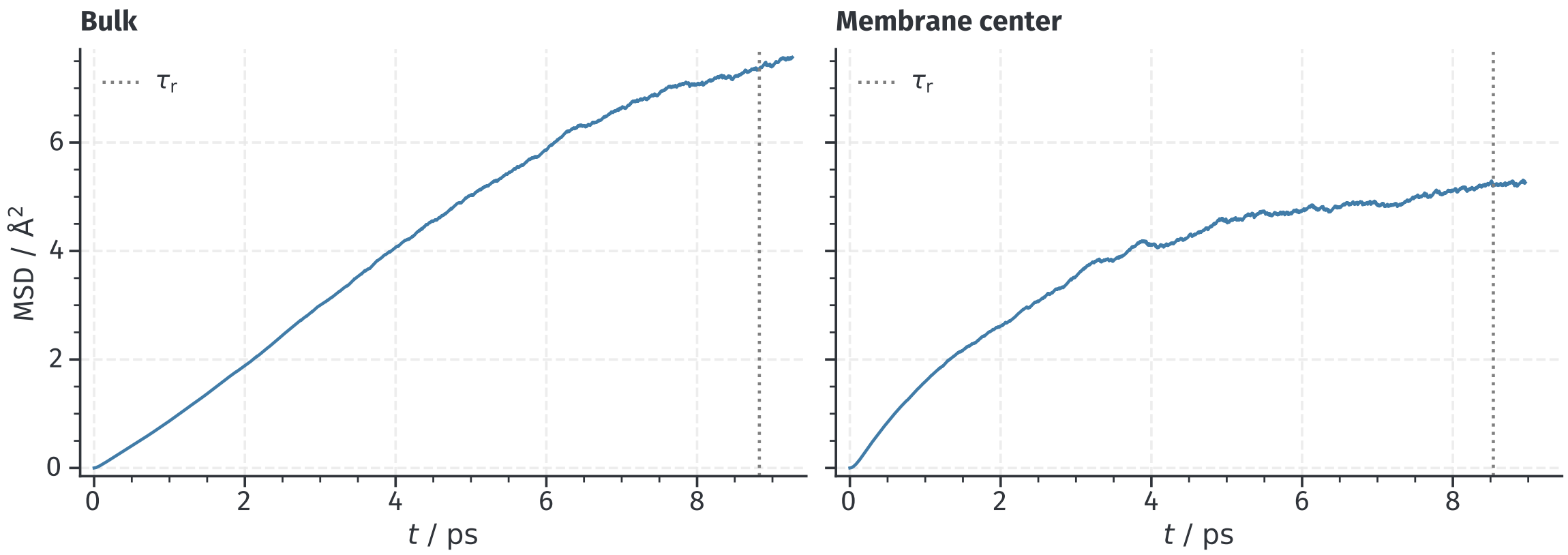

Figure S27: Local mean-square displacements computed from trajectory segments that remain within representative bulk and membrane-center intervals of width L in the SC/water system. The vertical dashed lines indicate the corresponding mean residence times τ_r.

S5.1.2 Survival Probabilities

A more direct test of the assumptions underlying the RTA is provided by the survival probability,

$$S(t) = P(T > t), \tag{S2}$$

where T denotes the remaining time until first exit from the interval.

For diffusion in a finite interval with absorbing boundaries, the survival probability is given by a sum of exponentially decaying eigenmodes,

$$S(t) = \sum_n a_n e^{-\lambda_n t}, \tag{S3}$$

where λ_n are the eigenvalues of the corresponding Smoluchowski operator.[6] At sufficiently long times, the lowest eigenmode dominates, so that the survival probability approaches a mono-exponential decay.

For the present comparison, we evaluate the analytical survival probability for homogeneous one-dimensional Smoluchowski diffusion with constant diffusivity. The diffusivity is fixed by the RTA relation,

$$D = \frac{L^2}{12\tau_r}, \tag{S4}$$

such that the comparison contains no adjustable parameters.

Figure S28 compares the survival probabilities obtained directly from MD with the corresponding Smoluchowski predictions. In the bulk region, close agreement is observed

over the entire time range, indicating that the first-passage statistics are well described by an effective diffusive process.

In contrast, the membrane-center region exhibits systematic deviations from the homogeneous Smoluchowski prediction. The MD survival probability decays more rapidly at short times but more slowly at long times, indicating a broader distribution of residence times than predicted by homogeneous diffusion while preserving the same mean residence time. Since the analytical prediction is constructed from the RTA diffusivity, both distributions necessarily yield the same average residence time, but differ in the detailed distribution of first-passage events.

The approximately mono-exponential long-time decay observed for the MD survival probability is consistent with the dominance of a single slow relaxation mode expected for diffusion in a finite interval. However, such behavior is not unique to Markovian dynamics and may also arise from spatially heterogeneous diffusivity or free-energy variations within the interval. Consequently, the observed deviations from the homogeneous Smoluchowski prediction cannot be uniquely attributed to memory effects.

Overall, these results suggest that the RTA diffusivity should be interpreted as an effective diffusivity characterizing transport over the length scale defined by the residence interval, rather than as a unique local transport coefficient governing all dynamical observables.

Finally, we emphasize that both the local MSD and the survival probability probe only selected aspects of the projected dynamics. The most stringent validation of the reduced Smoluchowski description is provided by comparison of the full propagator with unbiased MD simulations, as presented in the main text. Because the propagator determines the complete time-dependent transition probability, it provides a more global characterization of diffusive transport than local dynamical statistics.

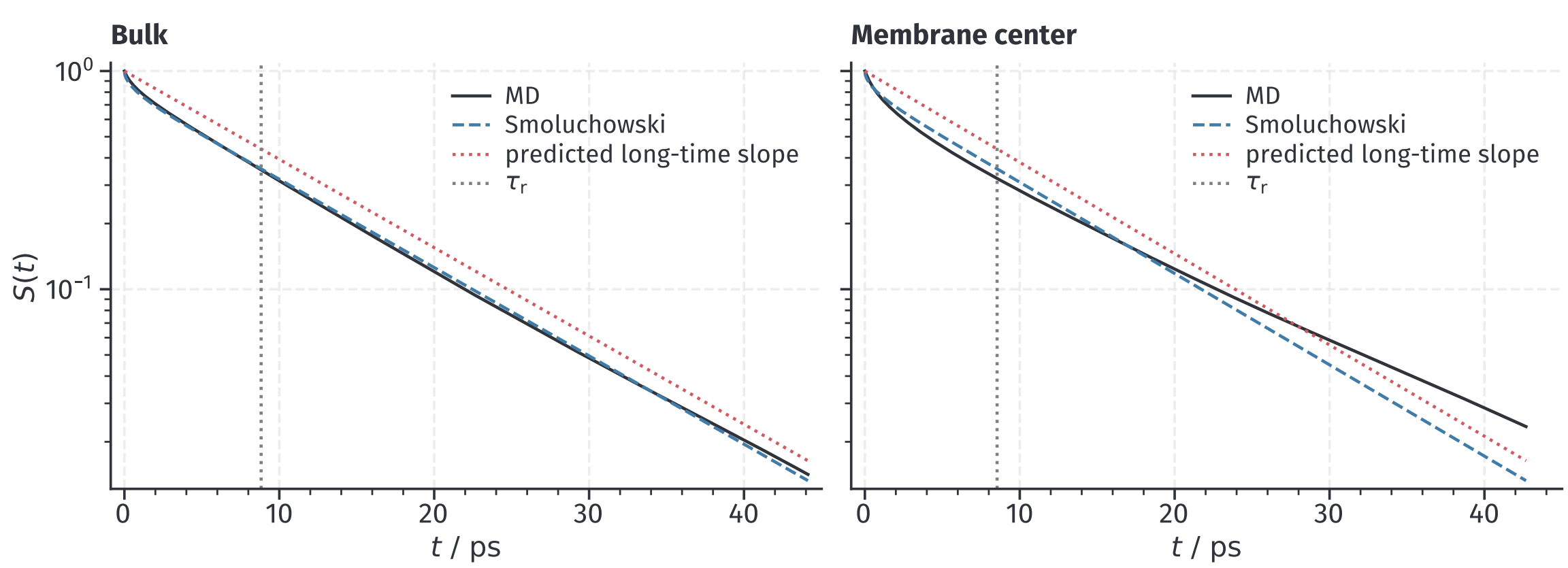

Figure S28: Survival probabilities obtained from MD for representative bulk and membrane-center intervals in the SC/water system compared with the analytical prediction for homogeneous one-dimensional Smoluchowski diffusion using the RTA diffusivity, $D = L^2/(12\tau_r)$, without additional fitting.

S5.1.3 Additional systems

Equivalent analyses for the remaining membrane systems considered in this work are shown in Figures S29, S30, and S31. In all cases, the local MSD exhibits the expected saturation on timescales comparable to the mean residence time, confirming that deviations from linearity are a generic consequence of confinement within the residence interval. Likewise, the survival probability is generally well described by the homogeneous Smoluchowski prediction in bulk-like regions, while systematic deviations are observed within the membrane interior. Despite these deviations, the RTA correctly reproduces the mean residence time by construction and should therefore be interpreted as providing an effective diffusivity on the scale of the residence interval. Since the full propagator comparison presented in the main text constitutes a strictly stronger validation of the reduced Smoluchowski model, we do not analyze these local statistics further.

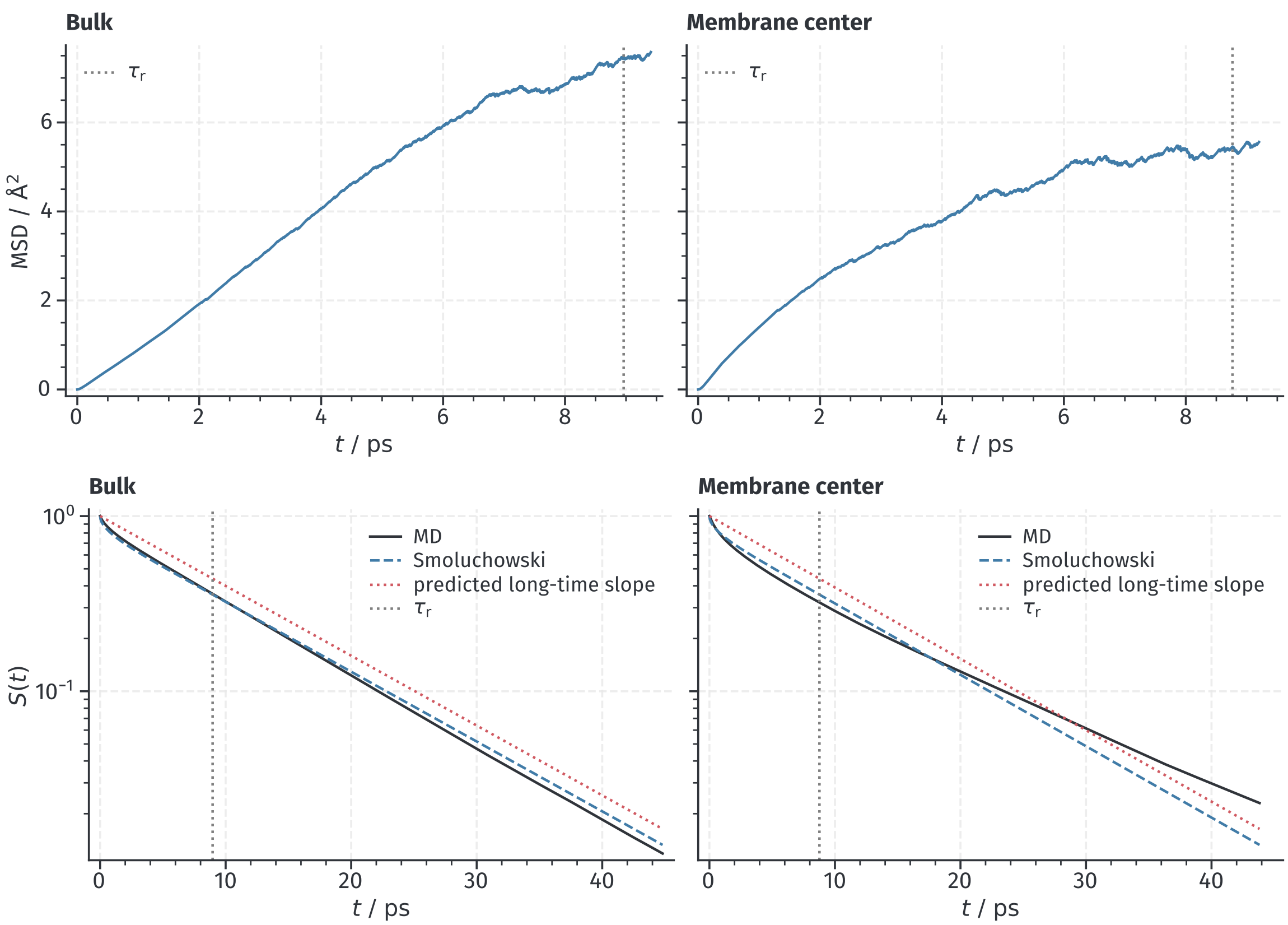

Figure S29: Local dynamical statistics for water permeation across the POPC membrane. Top: mean-square displacements computed from trajectory segments confined to representative bulk and membrane-center intervals. Bottom: corresponding survival probabilities obtained from MD compared with the analytical prediction for homogeneous Smoluchowski diffusion using the RTA diffusivity, $D = L^2/(12\tau_r)$.

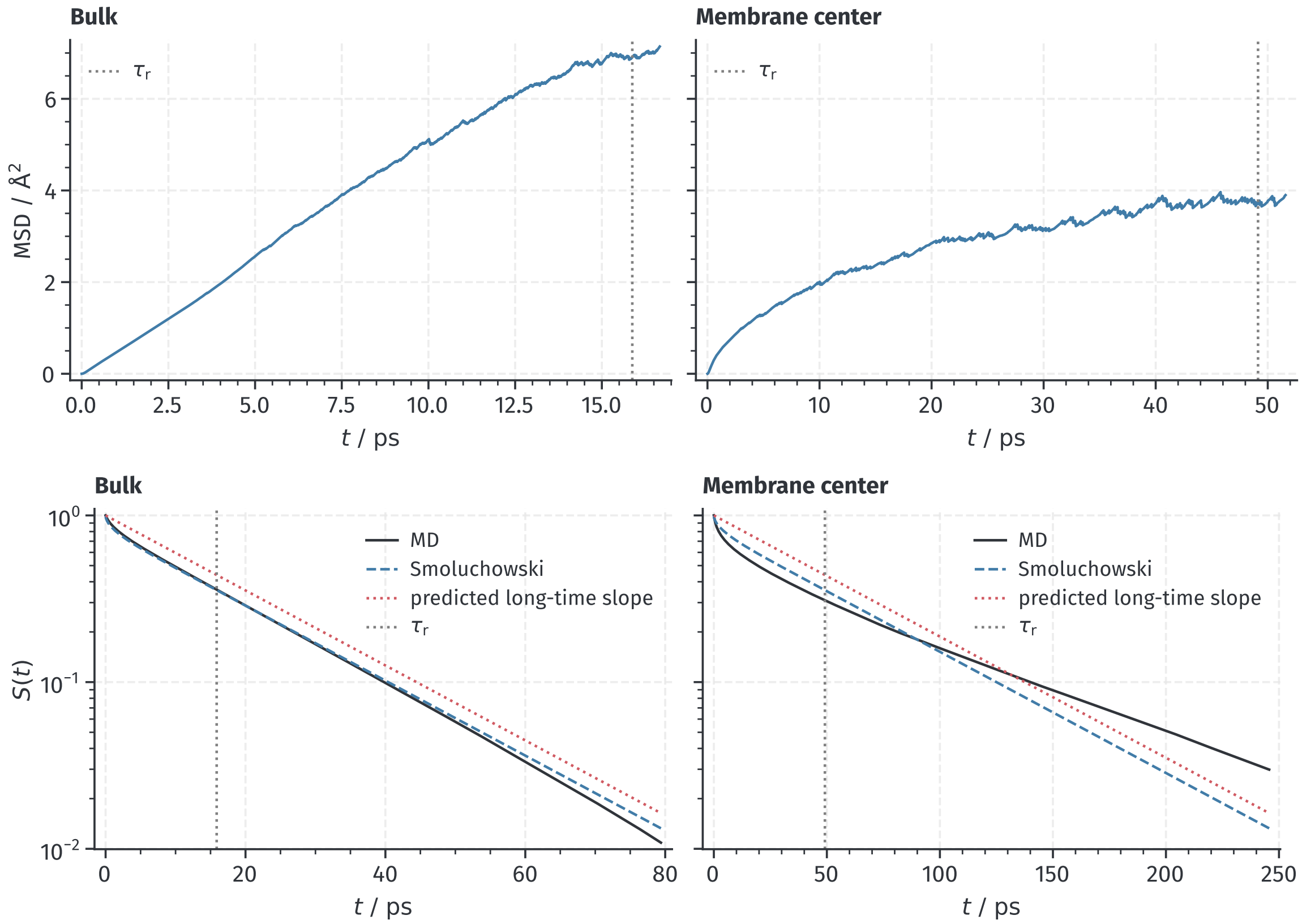

Figure S30: Same as Figure S29, but for acetone permeation across the SC membrane.

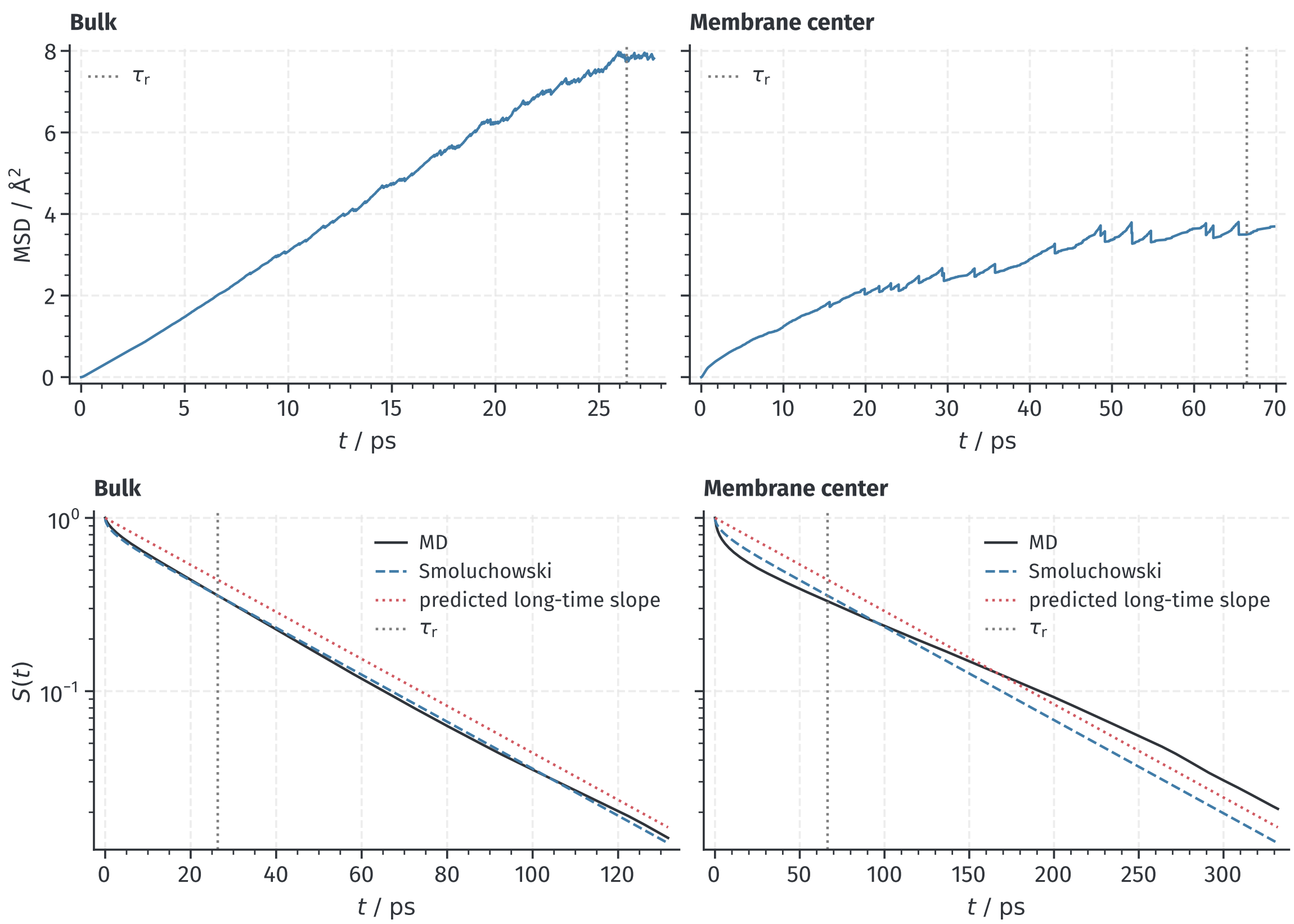

Figure S31: Same as Figure S29, but for 6-MHO permeation across the SC membrane.

S5.2 Uncertainty Quantification for Correlated Residence-Time Data

Reliable estimation of statistical uncertainties from correlated simulation data is a long-standing challenge. In the main text, uncertainties in the mean residence times were estimated using the automated blocking procedure of Jonsson,[7] which builds upon the blocking transformation of Flyvbjerg and Petersen.[8] This approach estimates the asymptotic variance directly from the data without requiring an explicit model for the temporal correlation structure.

Recently, McCluskey *et al.*[9] proposed an efficient framework for estimating bulk diffusion coefficients and their statistical uncertainties from a single simulation trajectory. In addition to their main estimator, they discuss a variance-normalization strategy in which the variance of the mean is obtained by dividing the variance of the underlying observable by the effective number of statistically independent samples. For mean-squared displacement (MSD) analyses, this procedure is particularly effective because the correlation structure arising from overlapping displacement windows is known analytically, allowing the effective number of independent observations to be estimated directly.

Residence-time data differ fundamentally from MSD data in this respect. Although successive residence times extracted from a trajectory are correlated, the corresponding

correlation structure is generally not known *a priori*. Correlations arise both from the use of overlapping starting points within a residence event and from slow dynamical processes in the underlying molecular system, such as persistent membrane fluctuations and hidden degrees of freedom. Consequently, a direct variance-normalization procedure analogous to that proposed for MSD data is not readily available.

To assess the robustness of the uncertainty estimates employed in the present work, we therefore compared the automated blocking procedure with two independent analyses based on the same residence-time data. First, variance estimates were computed after successive Flyvbjerg–Petersen blocking transformations without applying the automated stopping criterion. In each blocking transformation, neighboring observations are averaged pairwise, thereby increasing the effective block size while preserving the sample mean. The variance of the mean residence time is estimated from the variance of the blocked data divided by the remaining number of observations. As short-range correlations are progressively removed, the estimated variance increases toward an asymptotic plateau corresponding to effectively independent blocks.

Second, the residence-time series was partitioned into increasing numbers of non-overlapping contiguous batches. The variance of the mean residence time was estimated from the variance of the corresponding batch means divided by the number of batches. Increasing the number of batches reduces the batch size and therefore moves the estimator toward the result obtained by treating individual residence times as independent. Consequently, the estimated variance decreases with increasing batching level and approaches the asymptotic value only while the batches remain sufficiently large to average over the dominant temporal correlations.

The resulting variance estimates for a representative bulk interval and for the membrane center are shown in Figure S32. In both cases, the blocking transformation approaches the asymptotic variance from below, whereas the batch-means estimator approaches the same limiting value from above before decreasing toward the independent-sample limit for very small batch sizes. The automated Jonsson estimate lies within the plateau defined by these two independent analyses and is in excellent agreement with their common asymptotic value.

The close agreement between these conceptually different approaches indicates that the uncertainty estimates reported in the present work are not sensitive to the particular method used to account for temporal correlations. Although the variance-normalization framework of McCluskey *et al.* cannot be transferred directly to residence-time statistics because of the unknown correlation structure, the comparison presented here provides additional evidence that the automated blocking procedure yields reliable estimates of the asymptotic variance for the residence-time data considered in this work.

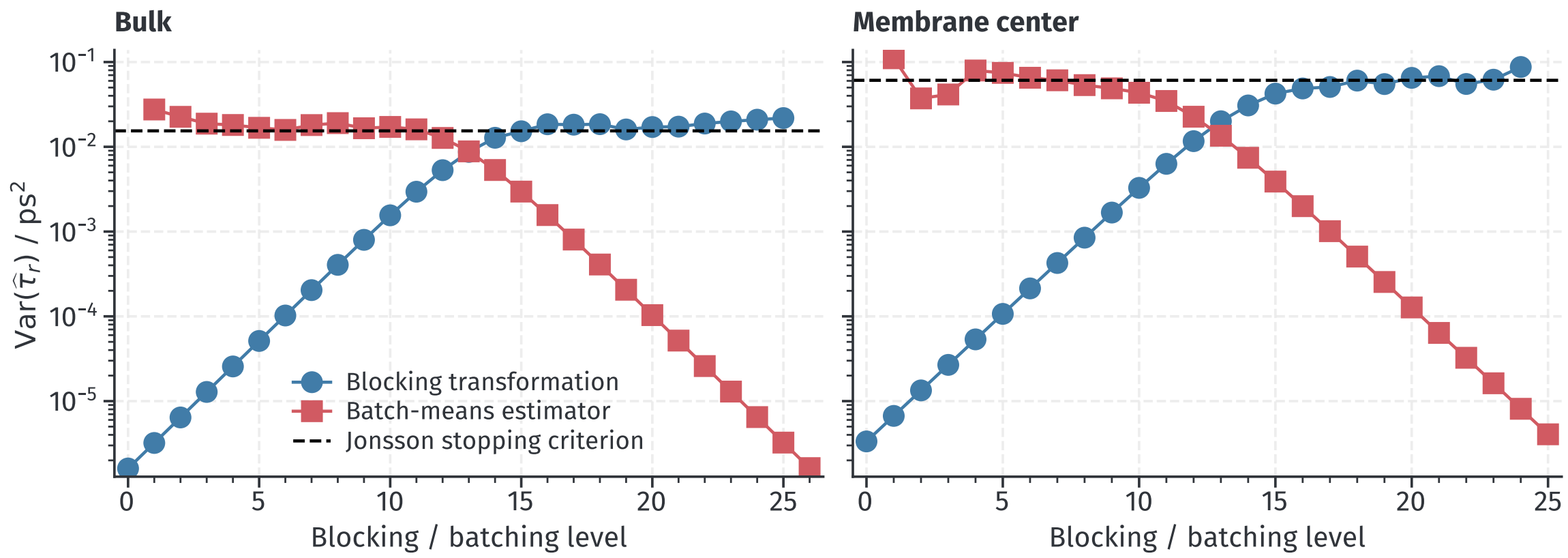

Figure S32: Comparison of variance estimates for the mean residence time obtained from successive Flyvbjerg–Petersen blocking transformations (circles), a non-overlapping batch-means estimator (squares), and the automated blocking procedure of Jonsson (horizontal dashed line). Results are shown for a representative bulk interval (left) and for the membrane center (right). The blocking estimate increases with successive blocking transformations as short-range correlations are removed, whereas the batch-means estimate decreases as the batch size is reduced. Both approaches converge to closely similar asymptotic values, in agreement with the automated blocking estimate.

S5.3 Dependence on the Residence Interval Width

The residence interval width, L, defines the spatial coarse-graining scale of the residence-time approach and therefore influences the effective diffusivity extracted from the first-passage statistics. If L is chosen too small, the residence times become increasingly sensitive to short-time non-diffusive dynamics and statistical noise. Conversely, if L is too large, spatial variations of the PMF and diffusivity within the interval may violate the assumption of locally constant transport properties underlying the RTA.

To examine the dependence of the estimated diffusivity on L, we performed the residence-time analysis using interval widths ranging from 2 Å to 10 Å for three representative regions of the SC/water system: bulk water ($z = -39$ Å), the membrane center ($z = 0$ Å), and the PMF maximum ($z = -14$ Å). The resulting diffusivities are shown in Figure S33.

For all three regions, the estimated diffusivity exhibits a plateau within statistical uncertainty over the interval-width range from 7.5 Å to 10 Å. This behavior indicates that the estimated diffusivity depends only weakly on the precise choice of L within this coarse-graining regime. The interval width adopted throughout the present work, $L = 7.5$ Å, lies within this plateau and therefore provides a robust estimate of the effective diffusivity. This conclusion is further supported by the propagator analysis presented in the main text, which demonstrates that diffusivities obtained with this choice of L yield a reduced Smoluchowski description over the relevant range of lag times.

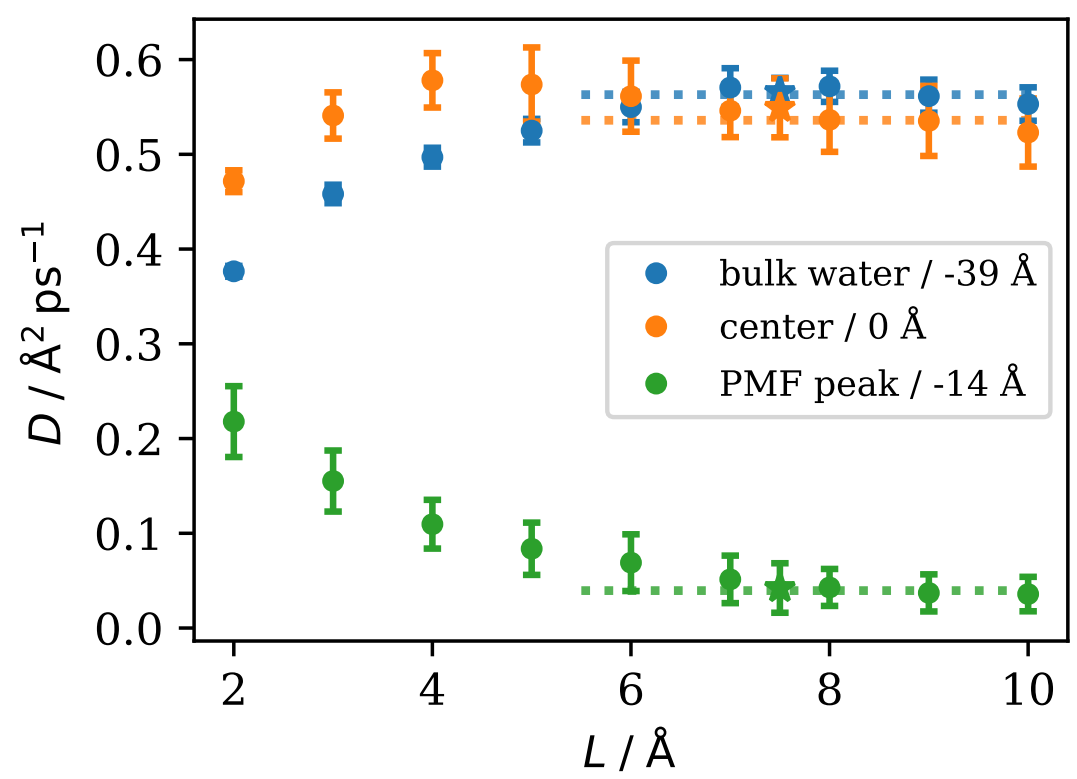

Figure S33: Dependence of the RTA diffusivity on the residence interval width L for three representative regions of the SC/water system: bulk water, the membrane center, and the PMF maximum. The plateau observed at larger interval widths indicates that the estimated diffusivities are only weakly sensitive to the precise choice of L within this coarse-graining regime.

References

[1] Ghysels, A.; Venable, R. M.; Pastor, R. W.; Hummer, G. Position-dependent diffusion tensors in anisotropic media from simulation: Oxygen transport in and through membranes. *J. Chem. Theory Comput.* **2017**, *13*, 2962–2976.

[2] Thomas, R.; Prabhakar, P. R.; Tobias, D. J.; von Domaros, M. Insights into dermal permeation of skin oil oxidation products from enhanced sampling molecular dynamics simulation. *J. Phys. Chem. B* **2025**, *129*, 1784–1794.

[3] Thomas, R.; Prabhakar, P. R.; Tobias, D. J.; von Domaros, M. Modeling diffusion and permeation across the stratum corneum lipid barrier. *ACS ES&T Air* **2026**, *3*, 580–589.

[4] Venable, R. M.; Krämer, A.; Pastor, R. W. Molecular dynamics simulations of membrane permeability. *Chem. Rev.* **2019**, *119*, 5954–5997.

[5] Reuter, M.; Joseph, E.; Lian, G.; Lunter, D. J. Presence of different ceramide species modulates barrier function and structure of stratum corneum lipid membranes: Insights from molecular dynamics simulations. *Mol. Pharm.* **2025**, *22*, 4280–4292.

[6] Redner, S. *A Guide to First-Passage Processes*; Cambridge University Press, 2001.

[7] Jonsson, M. Standard error estimation by an automated blocking method. *Phys. Rev. E* **2018**, *98*, 043304.

[8] Flyvbjerg, H.; Petersen, H. G. Error estimates on averages of correlated data. *J. Chem. Phys.* **1989**, *91*, 461–466.

[9] McCluskey, A. R.; Coles, S. W.; Morgan, B. J. Accurate Estimation of Diffusion Coefficients and Their Uncertainties from Computer Simulation. *J. Chem. Theory Comput.* **2025**, *21*, 79–87.
